# Raider of the lost *N-*glycans – Localizing rare and frequently overlooked IgG *N-*glycans with sulfation or bisecting LacNAc

**DOI:** 10.3389/fmolb.2025.1593708

**Published:** 2025-07-09

**Authors:** Robert Burock, Léa Chuzel, Thilo Kähne, Udo Reichl, Erdmann Rapp, René Hennig

**Affiliations:** ^1^ glyXera GmbH, Magdeburg, Germany; ^2^ New England Biolabs, Ipswich, MA, United States; ^3^ Institute of Experimental Internal Medicine, Medical School, Otto-von-Guericke-University Magdeburg, Magdeburg, Germany; ^4^ Bioprocess Engineering, Max-Planck-Institute for Dynamics of Complex Technical Systems, Magdeburg, Germany; ^5^ Bioprocess Engineering, Otto-von-Guericke-University Magdeburg, Magdeburg, Germany

**Keywords:** IgG *N-*glycosylation, Fab *N-*glycosylation, Fc *N-*glycosylation, bisecting LacNAc, sulfated *N-*glycans, xCGE-LIF, sulfation, sulfatase

## Abstract

Immunoglobulin G (IgG) is the most abundant immunoglobulin in human blood. Here it plays a central role in the immune system by recognizing antigens and mediating effector functions of the humoral immune defense. The role of IgG *N-*glycosylation in many of these processes is well known. However, low-abundant *N-*glycans with special features, like sulfation or galactosylated bisecting *N-*acetylglucosamine (GlcNAc), are rarely accounted for due to their challenging detection. These structures are frequently overlooked and their presence on IgG is disputed mainly because specialized enrichment and analysis strategies are required for their detection. Consequently, they are disregarded in studies of IgG *N-*glycosylation, which in general is well understood. But functional knowledge is mainly based on *N-*glycans found in IgGs Fc region that contains a conserved *N-*glycosylation site. In contrast, the influence of *N-*glycosylation within the Fab region is less well understood, partly because it is present at non-conserved glycosylation sites found on only 10%–25% of IgG. Here, we performed an in-depth analysis of released *N*-glycans derived from intact IgG, its Fab and its Fc regions. For this we combined proteolytic fragmentation of IgG obtained by affinity chromatography and exoglycosidase sequencing based on multiplexed capillary gel electrophoresis with laser-induced fluorescence detection (xCGE-LIF). By using these simple and readily available methods, we localized *N-*glycans bearing sulfation or galactosylated bisecting GlcNAc on IgG, and found them on IgA, too. Further, we proved sulfation of *N-*glycans using an apo-sulfatase in an epitope-directed glycan enrichment (EDGE-) profiling workflow. With our novel findings, we provide insights into hypothetical biological implications of these low-abundant *N-*glycan features and advocate for their inclusion in future studies of IgG *N-*glycosylation.

## 1 Introduction

Since the first discoveries of immune mechanisms, which led to the concept of antitoxins in blood, tremendous knowledge in the field of immunology has been gained at a rapid pace (Rees, 2014). Hence, nowadays we know that these antitoxins, among others, comprise of antibodies, and their structure and development are well characterized. Of these, immunoglobulin G (IgG) is the highest abundant antibody class in human blood (Vidarsson et al., 2014). IgG plays a pivotal role by binding antigens, such as viruses or bacteria, and mediating various immune responses. These effector functions include complement-dependent cytotoxicity, antibody-dependent cell-mediated cytotoxicity, phagocytosis, and anti-inflammatory responses (Shkunnikova et al., 2023). This central role made IgG an extremely interesting target for research since its discovery less than one hundred years ago (Black, 1997; Rees, 2014, pp. 1–46). Immense efforts have since been put into its structural and functional characterization, and the gained knowledge was rapidly adapted to make use of IgG. Hence, it is so well understood nowadays that it represents the leading class of biopharmaceuticals (Walsh and Walsh, 2022).

**FIGURE 1:**
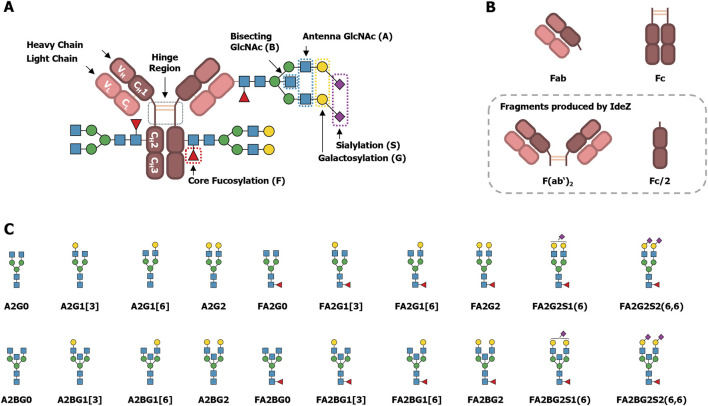
Schematic drawings of IgG’s structure, fragments and *N-*glycans. **(A)** IgG is assembled from two heavy and two light chains. Disulfide bridges link the heavy chains in the hinge region, and the light chains to the heavy chains between the C_H_1 and C_L_ domains. In the C_H_2 domain, a conserved *N-*glycosylation site is present. In contrast, non-conserved *N-*glycosylation sites might be found, usually in the V_H_ and V_L_ domains. **(B)** The variable domains together with the C_H_1 and C_L_ domains constitute two antigen binding fragments (Fabs). The C_H_2 and C_H_3 domains constitute the fragment crystallizable (Fc). Proteolytic cleavage by IdeZ below the hinge region results in a dimeric Fab fragment (F(ab’)_2_) and two halves of the Fc (Fc/2). These fragments will be referred to as Fab and Fc, respectively, because their exact composition is not important for the analysis of their *N-*glycosylation. **(C)** A representative selection of common IgG-derived *N-*glycans is shown. Glycan names follow the Oxford notation, with slight modifications, as explained by Cajic et al. (2023). Graphic representations are based on the symbol nomenclature for glycans (SNFG) (Neelamegham et al., 2019) with: Blue squares: GlcNAc. Red triangles: fucose. Green circles: mannose. Yellow circles: galactose. Purple diamonds: *N*-acetylneuraminic acid (Neu5Ac, α2,6-linked when tilted to the right).

Briefly summarized, human IgG is a glycoprotein with a molecular weight of approximately (∼) 150 kDa. It is composed of two light chains (LC) and two heavy chains (HC) linked by disulfide bonds within the hinge region (Figure 1A) (Damelang et al., 2024). Each heavy chain consists of one *N-*terminal variable (V_H_) domain and three constant (C_H_) domains. The light chains consist of one *N-*terminal variable (V_L_) domain and one constant (C_L_) domain. The diversity of amino acid sequences of the constant domains is limited by the HC variants that determine the IgG isotype (IgG1, IgG2, IgG3 or IgG4), and by the light chain type (λ or κ) (Murphy and Weaver, 2018, pp. 222–237). The C_H_2 and C_H_3 domains of the HC constitute the so-called Fc region (fragment crystallizable, Figure 1B), that mediates the effector functions. The Fc contains consensus sequons for *N-*glycosylation, Asn-X-Ser/Thr, where X may be any amino acid except proline. These conserved *N-*glycosylation sites at Asn297 in the C_H_2 domains are almost fully occupied (Stavenhagen et al., 2015; Chandler et al., 2019).

Together, the C_H_1, C_L_, V_H_ and V_L_ domains constitute the two Fab regions (antigen binding fragments, Figure 1B). The Fab usually does not contain a consensus sequon for *N-*glycosylation. But unlike the constant domains, the amino acid sequences of variable domains are highly diverse. This diversity is due to genetic rearrangements in the developing antibody-producing B cells, and it enables the binding of various antigens (Murphy and Weaver, 2018). Additionally, upon an initial antigen contact the antibodies undergo affinity maturation by a process called somatic hypermutation. Herein, the sequences encoding the V_H_ and V_L_ domains are even further mutated to enhance antigen binding. As a result, around 10%–25% of IgG acquire consensus sequons for *N*-glycosylation in the Fab region (van de Bovenkamp et al., 2016; Nimmerjahn et al., 2023), and these non-conserved glycosylation sites are not always fully occupied (Kissel et al., 2022).

In general, the *N-*glycosylation of IgG is a very well understood post-translational modification, and a plethora of reviews is available that cover different aspects, including the influence on the protein structure, regulation of effector functions, or correlations with various diseases (Arnold et al., 2007; Seeling et al., 2017; Cymer et al., 2018; Cobb, 2020; Nimmerjahn et al., 2023; Shkunnikova et al., 2023). Yet, most of this understanding is focused on the conserved *N-*glycosylation site Asn297 within the Fc region. The typically reported *N-*glycans at this site are diantennary glycans with a high degree of core fucosylation, varying degrees of galactosylation, as well as low levels of bisecting *N-*acetylglucosamine (GlcNAc) and sialylation, which are known modulators of the effector functions. Some representative examples of these structures are shown in Figure 1C, along with the nomenclature used in this manuscript (Cajic et al., 2023). In contrast to the Fc, the investigation of Fab *N-*glycosylation has only gained track in the last few years. The *N-*glycans found on the Fab are structurally similar to those found on the Fc but have a lower degree of fucosylation and higher degrees of bisection, galactosylation, and sialylation (Bondt et al., 2014). Known influences of Fab *N*-glycosylation include mostly effects on serum half-life (van de Bovenkamp et al., 2016), antigen binding (Leibiger et al., 1999; Xu et al., 2021; Kissel et al., 2022), as well as its role in autoimmune diseases and in B cell malignancies (Bondt et al., 2016; Rombouts et al., 2016; Vletter et al., 2020; Koers et al., 2023a; 2023b). Albeit, in some cases observations are limited to the presence or level of Fab glycosylation. For these reasons, functional understanding of individual Fab *N-*glycan features is still lagging behind that of Fc *N-*glycans (Vletter et al., 2020).

In addition to the established and frequently observed IgG *N-*glycans mentioned above, *N-*glycans on IgG can be modified by a sulfate group (Wang et al., 2017) or with an additional galactose on the bisecting GlcNAc (i.e., a bisecting *N-*acetyllactosamine, LacNAc) (Takegawa et al., 2005; Harvey et al., 2008). These structural features are challenging to recognize and therefore require dedicated analyses or enrichment strategies to compensate for their low abundance. In consequence, these structures are often unnoticed, disregarded, and in part actively challenged by the scientific community (Lauc et al., 2018). Therefore, they are not generally known or acknowledged modifications of IgG-derived *N-*glycans, even though they were discovered years ago. This is paradoxical given the significant interest in IgG and the adaptation of knowledge about it. Presumably, these two structural features are omitted in regular investigations of IgG *N-*glycosylation due to their challenging detection and identification by many commonly used and available analytical methods.

To solve these challenges, we used multiplexed capillary gel electrophoresis with laser-induced fluorescence detection (xCGE-LIF), which is a powerful tool for the analysis of antibody *N-*glycosylation (Cajic et al., 2021). In xCGE-LIF, glycans are labeled with a charged fluorescent dye and then electrophoretically separated inside a polymer-filled capillary, which leads to a high sensitivity and a high resolution of isomeric glycans (Hennig et al., 2016; Cajic et al., 2021; Burock et al., 2023). Due to the electrophoretic separation, the number of charges a glycan has by itself will considerably influence its migration time. Therefore, xCGE-LIF is particularly useful to separate glycans with additional charges, like those containing a sulfate group, from those lacking one. Hence, we used this analytical technique to identify IgG-derived sulfated *N-*glycans without the need for dedicated enrichment. Furthermore, we proteolytically separated the IgG Fab and Fc by using the IgG-specific protease IdeZ (Figure 1B) (Lannergård and Guss, 2006), and performed an in-depth *N-*glycan analysis of these fragments. As the sulfation of *N-*glycans can be difficult to verify, we ultimately proved the sulfation of the identified *N-*glycans by using a sulfatase that we have recently described (Chuzel, 2021; Chuzel et al., 2021). As shown during its characterization using IgA-derived *N-*glycans, this sulfatase is highly specific for 6-*O*-sulfated GlcNAc. We also meticulously evaluated the exoglycosidase digests, performed to expose 6-*O*-sulfated GlcNAc, in great detail. Thereby, we also re-discovered and localized long neglected, IgG-derived *N-*glycans with a bisecting LacNAc (Takegawa et al., 2005; Harvey et al., 2008), and identified them on IgA, too.

## 2 Materials and methods

### 2.1 Materials

Human IgA, IgG (batches IG1802-R22 and IG2017-01), Fab and F(ab’)_2_ were purchased from Athens Research & Technology (Georgia, USA).

Individual donor plasma samples were taken from a VisuCon™ normal donor set (Lot: NDS-0013) purchased from Affinity Biologicals Inc. (Ancaster, Canada). Three individual samples of the set were randomly selected and are referenced as follows: donor 1 (ID: ND105; LOT: 673760816), donor 2 (ID: ND96; LOT: 667210816), and donor 3 (ID: ND116; LOT: 667600816). VisuCon™-F frozen normal control plasma pools (batches 0009-52FCP and 0012-52FCP) were also purchased from Affinity Biologicals Inc. (Ancaster, Canada).

Buffers and solutions were prepared using ultrapure water with a conductivity of 1 S that was produced in-lab by a MilliQ® Reference A+ water purification system from Merck KGaA (Darmstadt, Germany).

Other chemicals were purchased from Merck KGaA (Darmstadt, Germany), unless specified otherwise.

### 2.2 Capture of intact IgG from blood plasma

Intact IgG was captured from blood plasma samples as reported before (Plomp et al., 2018; Beimdiek et al., 2022). Briefly, diluted blood plasma was applied to CaptureSelect™ IgG Fc affinity matrix (ThermoFisher Scientific, Darmstadt, Germany) placed inside a frit plate well and subsequently washed with phosphate buffered saline and water. Intact IgG was eluted from the affinity matrix with 100 mM formic acid and eluates were neutralized with ammonium bicarbonate solution.

### 2.3 On-bead IdeZ digest and fractionation of intact IgG into Fc and Fab

Based on a previously reported protocol (Bondt et al., 2014), intact IgG was captured from human plasma on CaptureSelect™ IgG Fc affinity matrix and washed with phosphate buffered saline and water. Then 240 U of the IgG-specific protease IdeZ (New England Biolabs Inc., Ipswich, USA) in 50 µL 50 mM ammonium bicarbonate buffer at pH 7.5 were added and incubated at 37°C in a humidified atmosphere overnight (∼19 h). The Fab containing enzyme solution was collected via centrifugation. The remaining Fab was washed off twice with 50 mM ammonium bicarbonate buffer and pooled with the collected enzyme solution as the Fab fraction. Affinity matrix-bound Fc was washed twice with water, eluted with 100 mM formic acid, and subsequently neutralized with ammonium bicarbonate solution.

### 2.4 *N-*glycan analysis using xCGE-LIF

*N-*glycans from blood plasma, IgA, intact IgG, Fab and Fc were prepared for xCGE-LIF-based *N-*glycan analysis by using the glyXprep™ kit (glyXera GmbH) as described before (Beimdiek et al., 2022). Briefly, *N-*glycans were released from denatured glycoproteins with PNGase F, labeled with 8-aminopyrene-1,3,6-trisulfonic acid (APTS), and purified via hydrophilic interaction chromatography in solid-phase extraction mode (HILIC-SPE), carefully following the kit instructions. Analyses of labeled *N-*glycans were conducted on a glyXboxCE™ system (glyXera, Magdeburg, Germany), based on a modified (“glyconeered”) 16 capillary 3130xl Genetic Analyzer equipped with a 50 cm capillary array filled with POP-7™ polymer. The samples were electrokinetically injected and analyzed with a running voltage of 15 kV for 40 min. Generated glycan data were analyzed with the glycoanalysis software glyXtoolCE™ (glyXera, Magdeburg, Germany), performing migration time alignment to double aligned migration time units (MTU”) for peak annotation and a relative quantification by normalization to total peak height (i.e., %TPH). Glycan peak annotations were additionally confirmed by xCGE-LIF-based re-analysis after exoglycosidase digestions using α2-3,6,8,9 neuraminidase A (SiaA), β1-3,4 galactosidase (GALase), α1-2,4,6 fucosidase O (FucO), α1-3,4 fucosidase, β-*N-*acetylglucosaminidase S (GlcNAcase), or α1-2,3,6 mannosidase (MANase), following the enzyme supplier’s instructions (all New England Biolabs Inc., Ipswich, USA).

### 2.5 EDGE-profiling of sulfated *N-*glycans

To prove the sulfation of *N*-glycans, an approach termed epitope-directed glycan enrichment (EDGE) was pursued. Usually, the used sulfatase F1-ORF13 (provided by New England Biolabs Inc., Ipswich, USA) cleaves off a sulfate group from 6-*O*-sulfated GlcNAc (Chuzel, 2021; Chuzel et al., 2021). But this enzyme can also bind terminal 6-*O*-sulfated GlcNAc as an apo-sulfatase, lacking Ca^2+^ ions as a cofactor needed for enzymatic activity (Chuzel, 2021). Here, this property was used in an EDGE-profiling workflow, in which sulfated *N-*glycans are bound by the enzyme and separated from non-sulfated *N-*glycans, as described before (Chuzel, 2021). Briefly, to enable enzyme binding, APTS-labeled *N-*glycans were first desialylated using SiaA and degalactosylated using GALase. Subsequently, *N-*glycans were incubated for 1 h at room temperature with 10 µg sulfatase in 50 mM MES buffer at pH 6 containing 1 mM EDTA for chelation of residual background Ca^2+^ ions. After incubation, the mixture was transferred into pre-washed NanoSep™ 30 kDa filters (Pall, Dreieich, Germany) to separate sulfated from non-sulfated *N-*glycans. Unbound, non-sulfated *N-*glycans were collected by centrifugation and pooled with two subsequent 120 µL water washing steps. To elute sulfated *N-*glycans from the apo-sulfatase, their binding was disrupted by incubation in 120 µL enzyme denaturing elution buffer (0.2% SDS in 0.1 M dithiothreitol) for 20 min at 60°C. The released sulfated *N-*glycans were collected by centrifugation and pooled with a single 200 µL water washing step. Both fractions (non-sulfated and sulfated *N-*glycans) were subjected to HILIC-SPE clean up using the glyXprep™ kit and analyzed on a glyXboxCE™ system as described before.

### 2.6 SDS-PAGE and *N-*glycan isolation from separated proteins

To ensure that the identified, low-abundant *N-*glycans originate only from IgG, the sample purity was initially assessed by non-reducing sodium dodecyl sulfate–polyacrylamide gel electrophoresis (SDS-PAGE) as described by Hennig et al. (2015). Therefore, blood plasma, intact IgG, Fab and Fc fractions were dissolved in NuPAGE™ LDS sample buffer, loaded onto a precast NuPAGE™ 4%–12% Bis-Tris gel (both ThermoFisher Scientific, Darmstadt, Germany) and electrophoretically separated. The protein size was evaluated using the PageRuler™ Plus pre-stained protein ladder (ThermoFisher Scientific, Darmstadt, Germany). During electrophoresis, intact IgG (∼150 kDa), Fab (∼100 kDa) and Fc (∼25 kDa) were separated from potential glycoprotein impurities with different molecular weights. Visible protein bands were then excised, reduced with dithiothreitol, alkylated with iodoacetamide, and de-*N-*glycosylated with PNGase F as described before (Hennig et al., 2015). *N-*glycans isolated from bands corresponding to intact IgG, Fab and Fc were prepared for xCGE-LIF based *N-*glycan analysis by using the glyXprep™ kit (glyXera GmbH, Magdeburg, Germany) as described above.

### 2.7 Identification of protein impurities with mass spectrometry

Proteomic analyses of the samples were performed as described before by Kolodziej et al. (2016) with two objectives. On the one hand, impurities should be recognized that might be present at very low levels and therefore might not appear as protein bands in SDS-PAGE. Therefore, the in-solution samples of intact IgG, Fab and Fc were reduced, alkylated and digested with trypsin for 12 h at 20°C. On the other hand, protein bands that do not correspond to intact IgG, Fab and Fc should be identified. Therefore, the cut-out and de-*N-*glycosylated protein bands from SDS-PAGE were digested with trypsin overnight at 37°C, and tryptic peptides were extracted.

Subsequent liquid chromatography–tandem mass spectrometry (LC-MS/MS) was performed on a hybrid dual pressure linear ion trap/orbitrap mass spectrometer (LTQ Orbitrap Velos Pro, Thermo Scientific, San Jose, CA, USA) equipped with an Ultimate 3000-nLC Ultra HPLC (Thermo Scientific, San Jose, CA, USA). Dried peptide fractions were dissolved in 10 μL 0.1% trifluoroacetic acid and subjected to a 200 cm µPAC™ RP C18-csA column (PharmaFluidics, Ghent, Belgium). Separation was achieved by applying a gradient from 2% to 35% acetonitrile in 0.1% formic acid over 180 min at a flow rate of 1.6 μL/min. The LTQ Orbitrap Velos Pro MS exclusively used collision-induced dissociation (CID) fragmentation when acquiring MS/MS spectra, consisting of an orbitrap full MS scan followed by up to 20 LTQ MS/MS experiments (TOP20) on the most abundant ions detected in the full MS scan. The essential MS settings were as follows: full MS (FTMS; resolution 60,000; m/z range 400–2,000); MS/MS (Linear Trap; minimum signal threshold 500; isolation width 2 Da; dynamic exclusion time setting 30 s; singly charged ions were excluded from selection). Normalized collision energy was set to 35%, and the activation time was set to 10 ms.

Raw data processing and protein identification of the high resolution orbitrap datasets were performed with *de novo* sequencing algorithms of PEAKS Studio 8.0 (Bioinformatics Solutions Inc., Waterloo, Canada) using the human SwissProt database. The false discovery rate was set to 0.1%.

## 3 Results

**FIGURE 2:**
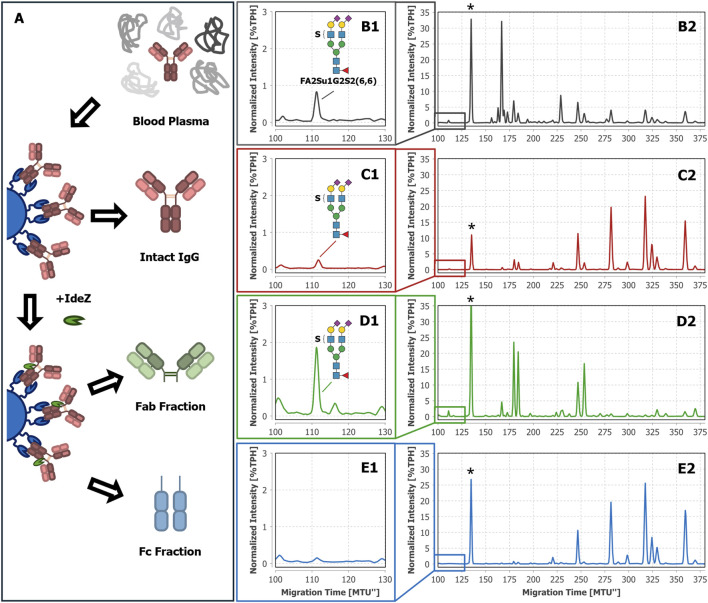
Tracking FA2Su1G2S2(6,6) in *N-*glycan fingerprints of blood plasma, intact IgG, Fab, and Fc of donor 1. The workflow starts with blood plasma as a complex glycoprotein mixture **(A)**. From this mixture, either intact IgG was selectively captured by affinity chromatography and eluted, or IgG was selectively captured, cleaved on-bead by IdeZ, and separated into Fab and Fc containing fractions. In the xCGE-LIF-based *N-*glycan fingerprints of blood plasma **(B1, B2)**, intact IgG **(C1, C2)**, and the Fab fraction **(D1, D2)** a FA2Su1G2S2(6,6) peak is found at ∼112 MTU”, but not in the fingerprint of the Fc fraction **(E1, E2)**. * Migration time alignment standard. Blue squares: GlcNAc. Red triangles: fucose. Green circles: mannose. Yellow circles: galactose. Purple diamonds: Neu5Ac (α2,6-linked when tilted to the right). S indicates a sulfate group.

Using MS-based techniques, we have recently been able to identify sulfated *N-*glycans on IgA (Cajic et al., 2023; Zuniga-Banuelos et al., 2025b). Regrettably, the glycoanalytical toolbox lacked suitable enzymes to directly prove the sulfation of *N-*glycans in complex mixtures by separation-based methods like xCGE-LIF. To provide such a missing tool, we previously have isolated and characterized a sulfatase (termed F1-ORF13) in a collaborative effort published by Chuzel et al. (2021). Its specificity and performance were determined using IgA-derived *N-*glycans, and more specifically, a core fucosylated, diantennary *N-*glycan with two galactoses, two α2-6 linked *N-*acetylneuraminic acids and one sulfate group attached to an antenna GlcNAc–FA2Su1G2S2(6,6). We could detect the same sulfated *N-*glycan in complex blood plasma, as shown in Figures 2B1, B2. While investigating whether other plasma proteins besides blood-derived IgA also carry FA2Su1G2S2(6,6), we have developed a particular interest in blood-derived IgG. Hence, we were encouraged to use the sulfatase to specifically prove *N-*glycan sulfation on this protein. Thus, as a first step we captured intact IgG from human blood plasma by affinity purification (Figure 2A) and subsequently subjected it to xCGE-LIF-based *N-*glycan analysis. Indeed, we were able to identify a peak in the fingerprint of the intact IgG-derived *N-*glycans with ∼0.24%TPH at a migration time of ∼112MTU”, matching FA2Su1G2S2(6,6) (Figures 2C1, C2).

As mentioned in the introduction, the two regions of IgG (Fab and Fc) mediate different functions. For this reason, the attachment site of the sulfated *N-*glycans can provide helpful hints to their biological role. To the best of our knowledge, neither the region of IgG to which the sulfated *N-*glycans are attached, nor details about the function of sulfated *N-*glycans on IgG are known yet. To localize the sulfated *N-*glycans, we separated the captured intact IgG into Fab and Fc by proteolytic on-bead cleavage using IdeZ (Figure 2A). In the Fab fraction we detected a peak with ∼1.82%TPH at the corresponding migration time of FA2Su1G2S2(6,6), too (Figures 2D1, D2). In contrast, we could not detect this peak in the Fc fraction (Figures 2E1, E2), implying that FA2Su1G2S2(6,6) is selectively localized on the Fab.

To exclude individual, donor-dependent bias of this observation, we analyzed additional samples from different sources: IgG isolated from two more individual donor-derived plasma samples (Supplementary Figures 1, 2), IgG isolated from two batches frozen normal control plasma pools (VisuCon™-F, Supplementary Figures 3, 4), two commercial IgG samples (Athens Research & Technology, Supplementary Figures 5, 6), as well as commercial Fab and F(ab’)_2_ samples (Athens Research & Technology, Supplementary Figure 7). We could identify FA2Su1G2S2(6,6) in all blood plasma, IgG, and Fab samples, but not in the associated Fc samples. This confirms that the seemingly exclusive localization of sulfated *N-*glycans on the Fab is not only a donor-specific observation but could be a general feature of human blood plasma-derived IgG.

The FA2Su1G2S2(6,6) peak also allows us to infer the Fab glycosylation frequency from the generated data. By correlating the normalized intensities of this peak in the *N-*glycan fingerprints of the Fab and intact IgG, a Fab glycosylation frequency of ∼15.2% can be estimated for donor 1. For all analyzed IgG samples, the Fab glycosylation frequency ranged from 14.2% to 25.8% (Supplementary Chapter 2; Supplementary Table 1).

**FIGURE 3:**
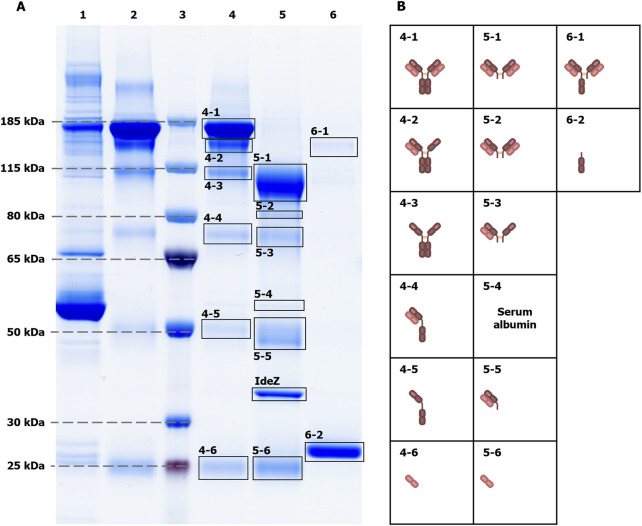
Non-reducing SDS-PAGE of intact IgG, Fab and Fc fractions to assess sample purity. The sample purity was assessed by non-reducing SDS-PAGE **(A)**. As references, blood plasma from donor 1 as the initial sample (lane 1), and an IgG control (Athens Research & Technology, 95% purity, lane 2) were loaded onto the gel. The apparent molecular weights of proteins were evaluated with protein size marker (lane 3). The electrophoretic separation of the intact IgG sample (lane 4), the Fab fraction (lane 5) and the Fc fraction (lane 6) showed additional protein bands. LC-MS/MS-based proteomics of cut-out protein bands **(B)**, in combination with apparent molecular weights, could identify them as unspecific degradation products of the main sample components that are intact IgG, Fab, Fc and IdeZ, respectively. For instance, band 4-3 at ∼100 kDa contains IgG that lost both light chains due to disrupted disulfide bonds that linked them to the heavy chains, resulting in a ∼50 kDa lower molecular weight. Exceptions are the serum albumin band 5-4, as the only visible impurity band that is not related to IgG, and the incompletely digested IgG band 6-1 in the Fc fraction.

**FIGURE 4:**
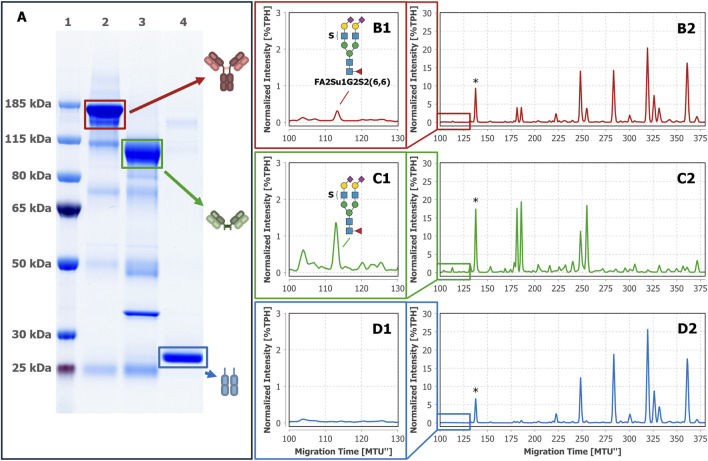
*N-*glycan analysis of intact IgG, Fab and Fc isolated from gel bands confirms the attachment of FA2Su1G2S2(6,6) to the Fab. After non-reducing SDS-PAGE **(A)** gel bands of intact IgG (lane 2), the separated Fab (lane 3) and the separated Fc (lane 4) from donor 1 were excised and subjected to in-gel *N-*glycan analysis by xCGE-LIF. A FA2Su1G2S2(6,6) peak at ∼112 MTU” was found on intact IgG (**B1, B2**) and on the separated Fab (**C1, C2**), but not on the separated Fc (**D1, D2**). Lane 1: protein size standards. * Migration time alignment standard. Blue squares: GlcNAc. Red triangles: fucose. Green circles: mannose. Yellow circles: galactose. Purple diamonds: Neu5Ac (α2,6-linked when tilted to the right). S indicates a sulfate group.

Hitherto, intact IgG, Fab and Fc were obtained by a single-step affinity purification, which in general already yields a high sample purity. However, especially in the case of extremely low-abundant target analytes like the sulfated *N*-glycans (see Figure 2C1 with FA2Su1G2S2(6,6) at 0.24%TPH), it is of special importance to ensure the absence of remaining glycoprotein contaminants. Even the smallest impurities might result in misinterpretation of the analysis results. To verify that the assignment of the sulfated *N-*glycan structures to IgG, and respectively its Fab, is accurate, we subjected the intact IgG, the Fab fraction and the Fc fraction shown in Figures 2C–E to an LC-MS/MS-based proteomic analysis. Notably, this approach identified only a few low-abundant glycoprotein impurities, as shown in Supplementary Tables 2–4. While major impurities like the serum albumin are not *N-*glycosylated and consequently do not impede the analysis, other co-immunoprecipitated proteins like components of the complement system could have influenced the analysis results. To further assess and improve the purity, intact IgG, the Fab fraction and the Fc fraction were subjected to non-reducing SDS-PAGE (Figure 3A). Thereby, glycoprotein impurities are separated from the target proteins in separate bands. Besides the bands corresponding to intact IgG at ∼150 kDa, Fab at ∼100 kDa, Fc at ∼25 kDa, and IdeZ at ∼36 kDa, several other bands were detected. We analyzed the proteins that constitute these bands by LC-MS/MS-based proteomics and considered both, the apparent molecular weights of the identified protein bands and the detected tryptic peptides. Thereby, these bands were identified as unspecific degradation products of IgG and Fab (Figure 3B; Supplementary Tables 5–18). Additionally, insufficiently depleted albumin was identified as a faint band in the Fab fraction (lane 5, band 5-4), and traces of incompletely digested IgG in the Fc fraction (lane 6, band 6-1). To entirely ensure that the traces of glycoprotein impurities did not impede our results and can be neglected, bands of intact IgG, Fab and Fc (Figure 4A) were subjected to in-gel *N-*glycan analysis, as shown in Figure 4B2–D2. This eliminated the impact of glycoprotein impurities with molecular weights different from intact IgG, Fab, or Fc. The analysis of *N-*glycans isolated from the protein bands corroborates the results obtained from samples in solution (Figure 2). A peak at the migration time of FA2Su1G2S2(6,6) can be found on intact IgG (Figure 4B1) and Fab (Figure 4C1), but not on Fc (Figure 4D1). Therefore, the impact of glycoprotein impurities on the analyses of samples in solution is minimal and can be neglected, as their abundance is most likely lower than the relative amount of FA2Su1G2S2(6,6).

**FIGURE 5:**
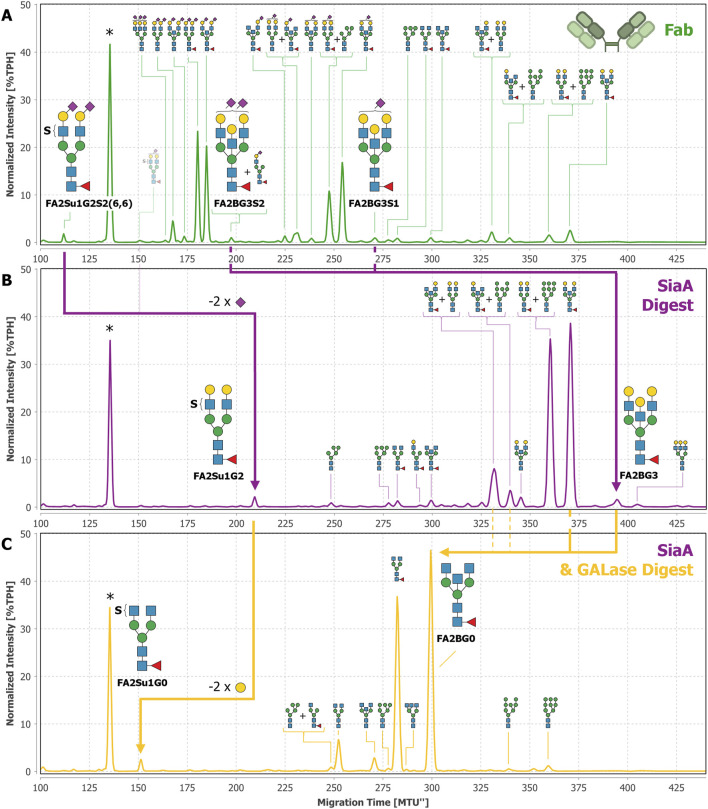
Exoglycosidase digests of Fab-derived *N-*glycans with SiaA and GALase confirm the identification of FA2Su1G2S2(6,6). *N-*glycans derived from the Fab of donor 1 **(A)** were digested with SiaA **(B)**. Thereby, FA2Su1G2S2(6,6) was digested to FA2Su1G2, shifting the peak from ∼112 MTU” to ∼209 MTU” (purple arrow). Trace amounts of the monosialylated FA2Su1G2S1(6) at ∼151 MTU” are indicated with a faded glycan depiction. At this position also an unknown compound appears, that is not reacting to any exoglycosidase digest. SiaA also hydrolyzed sialic acids from FA2BG3S2 at ∼197 MTU” and FA2BG3S1 at ∼271 MTU”, and thereby produced the FA2BG3 peak at ∼395 MTU”. By evaluating the amounts of FA2BG3 and FA1[3]G1 (peak at ∼294 MTU”) after the sialidase digest, most of the peak at ∼197 MTU” could be attributed to FA2BG3S2, which is indicated by the larger *N*-glycan picture. Upon digestion with GALase **(C)** FA2Su1G2 lost two galactoses, producing FA2Su1G0 and shifting the peak to ∼152 MTU” (yellow arrow). Furthermore, because three galactoses were hydrolyzed from FA2BG3 at ∼395 MTU”, and two from FA2BG2 at ∼371 MTU”, the FA2BG0 peak at ∼300MTU increased substantially (yellow arrow). Additionally, one galactose is hydrolyzed from FA2BG1[6] at ∼331 MTU” and FA2BG1[3] at ∼340 MTU”, thereby also contributing to the increase of the FA2BG0 peak at ∼300 MTU” (dashed yellow lines). * Migration time alignment standard. Blue squares: GlcNAc. Red triangles: fucose. Green circles: mannose. Yellow circles: galactose. Purple diamonds: Neu5Ac (α2,6-linked when tilted to the right, α2,3-linked when tilted to the left). S indicates a sulfate group. *N*-glycans that comigrate in a single peak are indicated by brackets and “+”.

So far, FA2Su1G2S2(6,6) has been exclusively identified by migration time matching to our database. To verify the identification of this *N*-glycan, we performed exoglycosidase digests using SiaA and GALase (Figure 5). The SiaA digest of Fab-derived *N-*glycans caused the loss of two sialic acids from FA2Su1G2S2(6,6), producing FA2Su1G2 that appears at ∼209 MTU” (Figure 5B). From this glycan two galactoses were hydrolyzed by a GALase digest, forming FA2Su1G0 that appears at ∼152 MTU” (Figure 5C). We were also able to identify these sulfated structures after exoglycosidase digests of blood plasma- and intact IgG-derived *N-*glycans (Supplementary Figures 8, 9), but not after exoglycosidase digests of Fc-derived *N-*glycans (Supplementary Figure 10). Furthermore, we had a closer look at the exoglycosidase digests of *N-*glycans derived from IgA, on which we initially detected the sulfated *N-*glycan FA2Su1G2S2(6,6) (Chuzel et al., 2021; Cajic et al., 2023). These digests revealed several additional sulfated glycans, like the disulfated diantennary FA2Su2G2S2(6,6) at ∼67 MTU”, as well as the triantennary FA3[2,4]Su1G3S2 at ∼151 MTU”, and their respective digestion products (Supplementary Figure 11).

After desialylation of the Fab-derived *N-*glycans we also observed a peak at ∼395 MTU” (Figure 5B). This peak also appeared after desialylation of intact IgG- and IgA-derived *N-*glycans (Supplementary Figures 9B, 11B) and in the commercial F(ab’)_2_ sample (Supplementary Figure 7B). To this peak we could not assign any existing database entry based on SiaA and GALase digests alone. Therefore, this peak was thoroughly investigated using additional exoglycosidases, namely GlcNAcase, α1-3,4 fucosidase and FucO, as shown in Supplementary Figure 13. Because the peak did not shift in the GlcNAcase digest (Supplementary Figure 13B) or in the α1-3,4 fucosidase digest (Supplementary Figure 13C), we excluded terminal GlcNAc residues or antenna fucosylation. In the FucO digest (Supplementary Figure 13D) the peak disappeared and shifted from ∼395 MTU” to ∼369 MTU”, thereby verifying the core fucosylation of the constituting *N-*glycan. In the GALase digest this *N-*glycan lost three galactose residues and shifted to FA2BG0 at ∼300 MTU” (Figure 5C), together with other core fucosylated, bisected *N-*glycans. Hence, we identified this glycan as a core fucosylated, fully galactosylated, diantennary *N-*glycan, containing a galactosylated bisecting GlcNAc, i.e., a bisecting LacNAc. Since this structure has not yet been described in the nomenclature system used here, this *N-*glycan was named FA2BG3. By considering characteristic migration time shifts caused by one or two sialic acids, respectively, we located the mono- and disialylated counterparts of this *N-*glycan in the untreated Fab sample. These are FA2BG3S1 at ∼271MTU” and FA2BG3S2 at ∼197 MTU” (Figure 5A). Albeit the peak at ∼197 MTU” is a multi-structure peak constituted by FA2BG3S2 and FA1[3]G1S1(6), we could evaluate its composition after the sialidase digest, forming FA2BG3 and FA1[3]G1. The careful evaluation of all normalized peak intensities revealed that the majority of the peak at ∼197 MTU” is constituted by FA2BG3S2.

Theoretically, the migration time of FA2BG3 (∼395 MTU”) could also correspond to large hybrid-type *N-*glycans with a core fucose. To exclude this possibility, and to be completely sure of the identification of FA2BG3, we also performed MANase and GlcNAcase digests after the GALase digest (Supplementary Figure 14). Both digests showed insufficient amounts of hybrid-type *N*-glycans present to account for the peak at ∼395 MTU”. Interestingly, through this meticulous analysis we also identified traces of triantennary *N-*glycans on the Fab (Figure 5). A comprehensive overview of all Fab-derived *N*-glycans can be found in Supplementary Table 19. Neither triantennary *N-*glycans nor *N-*glycans with a bisecting LacNAc could be identified on the Fc (Supplementary Figure 10). Using a similar approach, we also found peaks in HILIC-UPLC-FLD (hydrophilic interaction liquid chromatography - ultra-high performance liquid chromatography with fluorescence detection) to which we could assign FA2BG3S1, FA2BG3S2 and FA2Su1G2S2(6,6) (Supplementary Figure 15).

**FIGURE 6:**
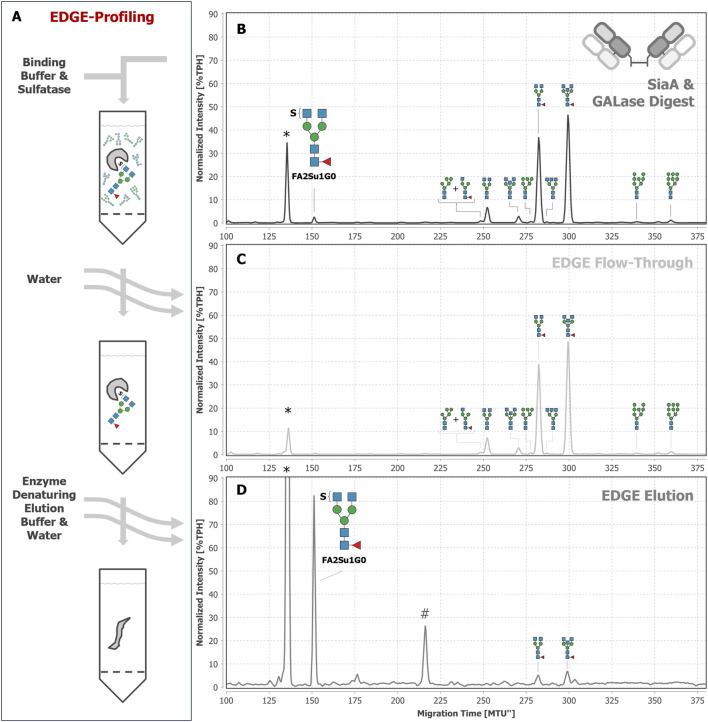
EDGE-profiling of Fab-derived *N-*glycans as final proof of *N-*glycan sulfation. Epitope directed glycan enrichment (EDGE-) profiling was performed to separate sulfated from non-sulfated *N-*glycans. Fab-derived *N-*glycans after SiaA and GALase treatment **(B)** were mixed with the sulfatase in binding buffer (50 mM MES buffer at pH 6 containing 1 mM EDTA) in the absence of available Ca^2+^ ions **(A)**. In this apo-sulfatase state, the enzyme only binds to 6-*O*-sulfated GlcNAc without hydrolyzing the sulfate and releasing the *N-*glycan. Unbound, non-sulfated *N-*glycans were collected in the flow-through together with subsequent water washing steps **(C)**. *N-*glycans with terminal 6-*O-*sulfated GlcNAc were eluted by denaturing the apo-sulfatase with enzyme denaturing elution buffer (0.2% SDS in 0.1 M dithiothreitol) and can be found in the elution fraction **(D)**. For instance, FA2SuG0 is found as a peak at ∼152 MTU”. * Migration time alignment standard. # unknown impurity introduced by enzyme solution (see explanation in Supplementary Figure 12). Blue squares: GlcNAc. Red triangles: fucose. Green circles: mannose. S indicates a sulfate group. *N*-glycans that comigrate in a single peak are indicated by brackets and “+“.

To ultimately prove the sulfation of the observed *N-*glycans, we made use of the previously described sulfatase in its apo-state in an EDGE-profiling workflow (Chuzel, 2021; Chuzel et al., 2021). This EDGE-profiling was performed on released *N*-glycans (its potential use for peptide- or protein-bound *N*-glycans is discussed in Supplementary Chapter 9). Lacking Ca^2+^ ions as a cofactor, the apo-sulfatase binds to terminal, 6-*O*-sulfated GlcNAc (Figures 6A, B), but does not release the *N-*glycan by hydrolysis of the sulfate group. Thereby, sulfated *N-*glycans are highly enriched in the elution fraction (Figure 6D), while non-sulfated *N-*glycans are collected in the flow-through (Figure 6C). By using this property, we ultimately proved the presence of the diantennary, sulfated *N*-glycan FA2Su1G0 on the Fab (Figure 6D) and on the intact IgG (Supplementary Figure 9E), while it could not be detected on the Fc (Supplementary Figure 10E). In addition to FA2Su1G0, we could prove the presence of the double sulfated FA2Su2G0 (peak at ∼76 MTU”) and the sulfated triantennary FA3[2,4]Su1G0 (peak at ∼176 MTU”) on IgA (Supplementary Figure 11E). In blood plasma, we additionally found the non-fucosylated sulfated A2Su1G0 (peak at ∼131 MTU”), and FA3[2,4]Su1G0, too (Supplementary Figure 8D).

## 4 Discussion

Sulfation is recognized as a biologically relevant modification of glycans (Bowman and Bertozzi, 1999) that is found for example in the nervous system (Scott and Panin, 2014; Yoshimura et al., 2017; Muñoz et al., 2019). It is also known to participate in various processes like degradation of pituitary gland hormones (Fiete et al., 1991), growth of neural cells (Martini et al., 1992), viral binding and reproduction (She et al., 2017; 2019), or lymphocyte homing (Rosen, 1999; Toyoda et al., 2009). But in many instances, like that of IgG, sulfated glycans are present in small quantities only, making their analysis a challenging endeavor that often requires targeted enrichment (Yagi et al., 2005; Lei et al., 2010; Wang et al., 2017; Yamada et al., 2020). Additionally, certain properties of glycan sulfation can further impede their analysis by state-of-the-art methods that are often used for IgG *N*-glycan profiling. E.g., it is crucial to account for suppressed ionization efficiency (Toyoda et al., 2009; Thomsson et al., 2010; Wang et al., 2017) and neutral in-source loss (Khoo and Yu, 2010; Chen et al., 2018) in mass spectrometry (MS) with the commonly used positive ion mode. Therefore, the negative ion mode is better suited for sulfated *N-*glycans (Yagi et al., 2005; Khoo and Yu, 2010; Wedepohl et al., 2010; A Balog et al., 2012; Hafkenscheid et al., 2017). Unfortunately, it suffers from lower overall sensitivity (Ni et al., 2013; Šoić et al., 2022) and collision-induced migration of sulfate groups (Kenny et al., 2011). Furthermore, adaptation of analysis workflows to the negative mode is not straightforward. E.g., when MS is combined with prior chromatography-based separations, the most commonly used glycan labels are optimized for maximum sensitivity in the positive mode (Šoić et al., 2022).

Alternatively, one can use separation-based techniques as stand-alone methods for the analysis of sulfated *N-*glycans (Yagi et al., 2005; Muñoz et al., 2019; Chuzel et al., 2021; Šoić et al., 2022; Cajic et al., 2023). (x)CGE-LIF is particularly well suited for this purpose due to its high sensitivity (Hennig et al., 2016; Cajic et al., 2021) and the pronounced migration time difference of sulfated glycans from their non-sulfated counterparts due to the additional negative charge (see, e.g., FA2G2S2(6,6) and FA2Su1G2S2(6,6) in Figure 5A). In contrast, the retention time difference in liquid chromatography is influenced by the column used and can be comparatively low. Yagi et al. (2005), e.g., reported a difference of 0.3 glucose units on an octadecyl silica phase, and a difference of one glucose unit on an amide phase for A2Su1G0 (1S1-200.1 according to their nomenclature). These small differences pose the risk that sulfated *N-*glycans comigrate with or are not well resolved from non-sulfated *N-*glycans (Supplementary Fig. 15), especially in highly complex samples, so that they are not recognized. Hence, xCGE-LIF is our method of choice to analyze sulfated *N-*glycans. But until recently, the available analytical toolbox to investigate sulfated *N-*glycans by separation-based techniques was quite limited. On the one hand, acidic hydrolysis of sulfate esters requires comparatively harsh conditions, and desulfation is hardly quantitative. E.g., Yagi et al. (2005) could only hydrolyze 50%–65% of sulfate esters after 4 h in 1 M HCl at 37°C. Such conditions might lead to a considerable loss of sialic acids, or at even higher temperatures and acid concentrations, ultimately degrade the whole glycan (Fan et al., 1994; He et al., 2018). On the other hand, enzymatic approaches, which have been rarely reported in the context of *N-*glycan analysis so far (Wedepohl et al., 2010; Chuzel et al., 2021), require milder conditions for quantitative desulfation. For this reason, an enzymatic approach was preferred here. Hence, we have used the previouslyisolated and characterized sulfatase to prove the presence of sulfate groups on a glycan by bindingin the absence of Ca^2+^ ions (Chuzel, 2021; Chuzel et al., 2021). In particular, we also analyzed the total *N*-glycome of IgA, that was used before as a generous source to isolate the sulfated *N-*glycan FA2Su1G2S2(6,6).

All immunoglobulins are secreted by B cells and therefore share a similar potential of enzymatic *N-*glycan processing, including sulfotransferases. Thus, it is a reasonable expectation to find *N-*glycan sulfation also on other immunoglobulins. Equipped with the suitable analytical capabilities for a straightforward identification, we set out to find sulfated *N-*glycans on other plasma proteins, and on immunoglobulins in particular. Notably, we were motivated by the study of Wang et al. (2017) to put a spotlight on IgG. The authors were able to detect sulfated *N-*glycans from IgG that was purified from human blood serum. They created a customized microchip device that makes use of titanium dioxide to enrich acidic *N-*glycans, including the sulfated structures. The results of Wang et al. were vigorously debated, with significant doubts being raised that low-abundant glycoprotein impurities might be the source of sulfated *N-*glycans (Lauc et al., 2018). This suspicion was successfully countered, and IgG as the origin of sulfated *N-*glycans was ultimately proven (Wang et al., 2018). Nonetheless, we also wanted to ensure that no low-abundant glycoproteins interfered with our analyses. By using SDS-PAGE as an additional purification step, we ruled out any considerable interference from other glycoproteins. Having ruled out such interferences, we have shown that xCGE-LIF can easily identify sulfated IgG *N-*glycans without the need for dedicated enrichment. Furthermore, for the first time we were able to localize the sulfated *N-*glycans and assign them to the Fab. Thus, further evidence and insight for the sulfation of IgG *N-*glycans is provided.

As stated before, the attachment site of *N-*glycans on IgG is biologically relevant, since the Fab and Fc regions are involved in different interactions of IgG. Thus, the evaluation of their origin is crucial to reveal their biological function. In contrast to the Fab, which is glycosylated in 15%–25% of IgG (see, e.g., Supplementary Table 1), the Fc is almost completely *N-*glycosylated and consequently accounts for the majority of the IgG *N-*glycome. Therefore, it is often implicitly conveyed as the predominant attachment site of all analyzed *N-*glycans, even when the total IgG *N-*glycome is analyzed. Typically, glycoproteomics is the method of choice to unequivocally assign a *N-*glycan to a specific glycosylation site of a protein. And indeed, glycoproteomics is a viable method for the site-specific identification of sulfated *N-*glycans (Toyoda et al., 2009; She et al., 2017; She et al., 2019; Zuniga-Banuelos et al., 2025a; Zuniga-Banuelos et al., 2025b). However, the glycoproteomic analysis of polyclonal antibodies becomes particularly difficult as soon as non-conserved glycosylation sites are to be considered. Complementary to the already high structural diversity of glycans, glycoproteomic analyses introduce another layer of complexity through the amino acid sequence of the glycopeptide. This usually poses no great challenge for glycosylation sites within known peptide sequences. But this critical prerequisite is not given for the Fabs of polyclonal antibodies, because most *N-*glycosylation sites of this region are introduced to hypervariable regions of the V_H_ or V_L_ domains during affinity maturation (van de Bovenkamp et al., 2016; Corsiero et al., 2020), and only five gene loci encode *N-*glycosylation consensus sequons within the variable Fab domains (Lefranc, 2011). Thus, the sheer variability of unknown amino acid sequences makes the glycoproteomic analysis of polyclonal Fabs practically impossible (Bondt et al., 2014; Shkunnikova et al., 2023). For this reason, we decided to separate the Fab from the Fc part by proteolytic digestion of blood plasma-derived polyclonal IgG to localize the sulfated *N-*glycans. Using this widely adopted strategy for the separate study of Fab and Fc *N-*glycosylation (Holland et al., 2006; Anumula, 2012; Bondt et al., 2014; Bondt et al., 2016; Mahan et al., 2015; Rombouts et al., 2016; Hafkenscheid et al., 2017) we could find *N-*glycan sulfation in all our analyzed samples on the Fab, but not on the Fc. It is therefore plausible that these structures were so far not found in glycoproteomic analyses of polyclonal IgG, which focus on the conserved *N-*glycosylation site of the Fc region.

The biological function of the sulfated *N-*glycans on the Fab has yet to be determined in dedicated experiments, and to this point we can only hypothesize about possible functional or molecular implications of this modification. Recently, Huang et al. (2022) chemoenzymatically produced sulfated Fc glycoforms of the monoclonal antibody rituximab and showed that the sulfation of the Fc *N-*glycans did not significantly affect the binding to Fcγ receptor FcγRIIIa-V158. In contrast to this approach, our investigation has shown that sulfated *N-*glycans of blood plasma-derived IgG are located on the Fab and not on the Fc. Therefore, functional studies should focus on sulfated Fab glycoforms to elucidate their *in vivo* purpose.

As already mentioned, antibodies are secreted by B cells and circulate in the bloodstream. There, they are predominantly present in two forms: cell-bound via Fc-FcγR interactions or freely circulating. Thus, sulfated *N-*glycans could modulate the biological functions of the secreted IgG in different ways. E.g., it is known that the Fab can also interact with FcγRs, and that the mere presence of Fab *N-*glycans can influence the binding of IgG to FcγRs due to steric, electrostatic or thermodynamic effects (Brinkhaus et al., 2022; Kosuge et al., 2022; Volkov et al., 2023). In this interaction, the additional, negatively charged sulfate on *N-*glycans might counteract a positive charge of the Fab (Kosuge et al., 2022). Furthermore, sulfated Fab *N-*glycans of secreted, freely circulating IgG might act as ligands for adhesion molecules on the cell surface, thereby influencing cell-cell interactions that are involved in lymphocyte homing or migration (Rosen, 1999; Walker and Smith, 2008; Nitschke, 2009). IgG might be mediating these processes by recruiting cells via Fc-FcγR binding. Additionally, a possible involvement of the sulfated *N*-glycans in B cell malignancies is conceivable, in which Fab glycosylation is known to play a role (Vletter et al., 2020).

However, B cells not only secrete IgG, but they also express membrane-bound analogues of antibodies as part of the antigen-specific B cell receptor (BCR). Therefore, it might be possible that the sulfated *N-*glycans we found originate from the BCR and its Fab, respectively. On the B cell membrane, the sulfated *N*-glycans might influence the interaction of the BCR with other membrane receptors of the B cell. In particular, CD22 (Siglec-2) is a considerable inhibitory receptor, because the α2-6-sialylated 6-sulfo-LacNAc motif present on FA2Su1G2S2(6,6) is one of its known ligands (Blixt et al., 2004; Kimura et al., 2007; MacAuley et al., 2014). Recently, Kissel et al. (2022) published a thorough investigation on the influence of Fab *N-*glycosylation in the interaction of the BCR and CD22. Their work focused on anti-citrullinated protein antibodies (ACPAs) and their respective BCRs cloned from patients and expressed by HEK or Ramos B cells. The authors showed that *N-*glycosylation of the Fab, including an abundance of α2-6-sialylated complex type *N-*glycans, led to decreased antigen binding capacity and lower downregulation of B cell activation. By knocking out CD22, a similar increase in BCR signaling capacity was observed for both, glycosylated and non-glycosylated ACPA variants, indicating a regulation of B cell activity that is independent of CD22. But compared to its non-sulfated counterpart, the α2-6-sialylated 6-sulfo-LacNAc motif has a higher affinity to CD22 (Blixt et al., 2004; Kimura et al., 2007; MacAuley et al., 2014). Therefore, sulfation of Fab *N-*glycans might act as a trigger for interaction with CD22, thereby modulating the threshold for B cell activation (Walker and Smith, 2008; Nitschke, 2009; MacAuley et al., 2014). Thus, sulfation of Fab *N-*glycans might modulate disease severity or progression through regulating the B cell activity. However, the exact mechanism and effect of this interaction has to be elucidated in future studies.

By a meticulous evaluation of exoglycosidase digests, we also identified *N-*glycans with bisecting LacNAc, i.e., galactosylated bisecting GlcNAc, and localized them on the IgG Fab as well. Like sulfated *N*-glycans, this special *N-*glycan motif is not easily identified with MS-based methods, because its mass is identical to a third *N*-glycan antenna. As described by Harvey et al. (2008), such isomers can be difficult to distinguish in complex samples when using the positive detection mode, because the types and abundances of different fragment ions can lead to ambiguous structural conclusions. For this reason, signals are often assigned to the more plausible seeming, triantennary *N-*glycan structures. In contrast to the considerable knowledge about the function and influence of bisecting GlcNAc in the *N-*glycosylation of the IgG Fc (Shkunnikova et al., 2023), not much is known about bisecting LacNAc on *N-*glycans in general. So far, the presence of this glycan motif on IgG is known from complex type glycans (Takegawa et al., 2005; Harvey et al., 2008). Apart from that, it is mostly known from hybrid-type *N-*glycans. Such structures were observed in a mouse model of congenital disorder of glycosylation type IIa, characterized by a deficiency of GlcNAc transferase II that is necessary for the synthesis of complex type *N-*glycans (Wang et al., 2001). More recently, the bisecting LacNAc motif was also found on hybrid type *N-*glycans in human brain and on IgA, where it can be modified even further by sialic acid or fucose (Helm et al., 2021; 2022). Here, we also report the identification of the two complex type *N-*glycans FA2BG3S1 and FA2BG3S2 on IgA (Supplementary Figure 11A), thereby expanding the known diversity of *N-*glycans with a bisecting LacNAc motif found on this antibody class (Helm et al., 2021). Given these rare occasions of identification, much more research about the functional implication of a bisecting LacNAc is necessary, and such studies could be facilitated by xCGE-LIF-based glycan analysis.

Considering the increasing power of analytical methods and the option to simply chemoenzymatically produce such low-abundant *N-*glycans (Weiss et al., 2020; Huang et al., 2022), we strongly advocate for the inclusion of *N-*glycans with sulfation, or bisecting LacNAc, in future glycosylation-related studies to elucidate functional relationships or in searches of glycan-based biomarkers.

## 5 Conclusion and outlook

In this paper we combined simple and readily available methods of affinity chromatography-based capture, proteolytic cleavage, and sophisticated xCGE-LIF-based glycan analysis to study the *N*-glycosylation of blood-derived IgG. Using this approach, we could assign rarely reported, low-abundant *N-*glycans to the Fab region. These included both, *N-*glycans bearing a bisecting LacNAc or 6-*O*-sulfation of GlcNAc, that was proven with a sulfatase in an EDGE-profiling workflow. These features could be easily identified or distinguished from isomeric structures by xCGE-LIF without dedicated enrichment or analysis strategies. Being harnessed with this straightforward, xCGE-LIF-based analytical approach, we anticipate a rapid increase in knowledge about the role of these peculiar, Fab-borne *N-*glycans and an advancing of the ever-growing insights into antibody-mediated immune regulation.

## Data Availability

Raw data files of the proteomic LC-MS/MS analyses have been deposited in the ProteomeXchange Consortium via the PRIDE repository (dataset identifier: PXD065180). The original, xCGE-LIF-based glycomic data presented in the study are available in the article/supplementary material. Further inquiries can be directed to the corresponding author.

## References

[B1] A Balog C. I. Stavenhagen K. J Fung W. L. Koeleman C. A. McDonnell L. A. Verhoeven A. (2012). N-glycosylation of colorectal cancer tissues: a liquid chromatography and mass spectrometry-based investigation. Mol. Cell. Proteomics 11, 571–585. 10.1074/mcp.M111.011601 22573871 PMC3434767

[B2] Anumula K. R. (2012). Quantitative glycan profiling of normal human plasma derived immunoglobulin and its fragments Fab and Fc. J. Immunol. Methods 382, 167–176. 10.1016/j.jim.2012.05.022 22683540

[B3] Arnold J. N. Wormald M. R. Sim R. B. Rudd P. M. Dwek R. A. (2007). The impact of glycosylation on the biological function and structure of human immunoglobulins. Annu. Rev. Immunol. 25, 21–50. 10.1146/annurev.immunol.25.022106.141702 17029568

[B4] Beimdiek J. Hennig R. Burock R. Puk O. Biskup S. Rapp E. (2022). Serum *N* -glycomics of a novel CDG-IIb patient reveals aberrant IgG glycosylation. Glycobiology 32, 380–390. 10.1093/glycob/cwac003 35137040

[B5] Black C. A. (1997). A brief history of the discovery of the immunoglobulins and the origin of the modern immunoglobulin nomenclature. Immunol. Cell Biol. 75, 65–68. 10.1038/icb.1997.10 9046436

[B6] Blixt O. Head S. Mondala T. Scanlan C. Huflejt M. E. Alvarez R. (2004). Printed covalent glycan array for ligand profiling of diverse glycan binding proteins. Proc. Natl. Acad. Sci. 101, 17033–17038. 10.1073/pnas.0407902101 15563589 PMC534418

[B7] Bondt A. Rombouts Y. Selman M. H. J. Hensbergen P. J. Reiding K. R. Hazes J. M. W. (2014). Immunoglobulin G (IgG) fab glycosylation analysis using a new mass spectrometric high-throughput profiling method reveals pregnancy-associated changes. Mol. Cell. Proteomics 13, 3029–3039. 10.1074/mcp.M114.039537 25004930 PMC4223489

[B8] Bondt A. Wuhrer M. Kuijper T. M. Hazes J. M. W. Dolhain R. J. E. M. (2016). Fab glycosylation of immunoglobulin G does not associate with improvement of rheumatoid arthritis during pregnancy. Arthritis Res. Ther. 18, 274. 10.1186/s13075-016-1172-1 27887659 PMC5123206

[B9] Bowman K. G. Bertozzi C. R. (1999). Carbohydrate sulfotransferases: mediators of extracellular communication. Chem. Biol. 6, R9–R22. 10.1016/S1074-5521(99)80014-3 9889154

[B10] Brinkhaus M. Pannecoucke E. van der Kooi E. J. Bentlage A. E. H. Derksen N. I. L. Andries J. (2022). The Fab region of IgG impairs the internalization pathway of FcRn upon Fc engagement. Nat. Commun. 13, 6073. 10.1038/s41467-022-33764-1 36241613 PMC9568614

[B11] Burock R. Cajic S. Hennig R. Buettner F. F. R. Reichl U. Rapp E. (2023). Reliable N-glycan analysis–removal of frequently occurring oligosaccharide impurities by enzymatic degradation. Molecules 28, 1843. 10.3390/molecules28041843 36838829 PMC9967028

[B12] Cajic S. Hennig R. Burock R. Rapp E. (2021). Capillary (gel) electrophoresis-based methods for immunoglobulin (G) glycosylation analysis. Exp. Suppl. 112, 137–172. 10.1007/978-3-030-76912-3_4 34687009

[B13] Cajic S. Hennig R. Grote V. Reichl U. Rapp E. (2023). Removable dyes—the missing link for in-depth N-glycan analysis via multi-method approaches. Engineering 26, 132–150. 10.1016/j.eng.2023.02.016

[B14] Chandler K. B. Mehta N. Leon D. R. Suscovich T. J. Alter G. Costello C. E. (2019). Multi-isotype glycoproteomic characterization of serum antibody heavy chains reveals isotype-and subclass-specific n-glycosylation profiles. Mol. Cell. Proteomics 18, 686–703. 10.1074/mcp.RA118.001185 30659065 PMC6442369

[B15] Chen J. Y. Huang H. H. Yu S. Y. Wu S. J. Kannagi R. Khoo K. H. (2018). Concerted mass spectrometry-based glycomic approach for precision mapping of sulfo sialylated N-glycans on human peripheral blood mononuclear cells and lymphocytes. Glycobiology 28, 9–20. 10.1093/glycob/cwx091 29087466

[B17] Chuzel L. (2021). Application of functional metagenomics to the field of glycobiology. [PhD thesis]. Magdeburg: Otto-von-Guericke-Universität Magdeburg. Available online at: https://d-nb.info/1249017475/04.

[B16] Chuzel L. Fossa S. L. Boisvert M. L. Cajic S. Hennig R. Ganatra M. B. (2021). Combining functional metagenomics and glycoanalytics to identify enzymes that facilitate structural characterization of sulfated N-glycans. Microb. Cell Fact. 20, 162. 10.1186/s12934-021-01652-w 34419057 PMC8379841

[B18] Cobb B. A. (2020). The history of IgG glycosylation and where we are now. Glycobiology 30, 202–213. 10.1093/glycob/cwz065 31504525 PMC7109348

[B19] Corsiero E. Carlotti E. Jagemann L. Perretti M. Pitzalis C. Bombardieri M. (2020). H and L Chain affinity maturation and/or fab N -glycosylation influence immunoreactivity toward neutrophil extracellular trap antigens in rheumatoid arthritis synovial B cell clones. J. Immunol. 204, 2374–2379. 10.4049/jimmunol.1901457 32221039 PMC7167462

[B20] Cymer F. Beck H. Rohde A. Reusch D. (2018). Therapeutic monoclonal antibody N-glycosylation – structure, function and therapeutic potential. Biologicals 52, 1–11. 10.1016/j.biologicals.2017.11.001 29239840

[B21] Damelang T. Brinkhaus M. van Osch T. L. J. Schuurman J. Labrijn A. F. Rispens T. (2024). Impact of structural modifications of IgG antibodies on effector functions. Front. Immunol. 14, 1304365. 10.3389/fimmu.2023.1304365 38259472 PMC10800522

[B22] Fan J. Q. Namiki Y. Matsuoka K. Lee Y. C. (1994). Comparison of acid hydrolytic conditions for asn-linked oligosaccharides. Anal. Biochem. 219, 375–378. 10.1006/abio.1994.1280 8080096

[B23] Fiete D. Srivastava V. Hindsgaul O. Baenziger J. U. (1991). A hepatic reticuloendothelial cell receptor specific for SO4-4GalNAcβ1, 4GlcNAcβ1,2Manα that mediates rapid clearance of lutropin. Cell 67, 1103–1110. 10.1016/0092-8674(91)90287-9 1662117

[B24] Hafkenscheid L. Bondt A. Scherer H. U. Huizinga T. W. J. Wuhrer M. Toes R. E. M. (2017). Structural analysis of variable domain glycosylation of anti-citrullinated protein antibodies in rheumatoid arthritis reveals the presence of highly sialylated glycans. Mol. Cell. Proteomics 16, 278–287. 10.1074/mcp.M116.062919 27956708 PMC5294214

[B25] Harvey D. J. Crispin M. Scanlan C. Singer B. B. Lucka L. Chang V. T. (2008). Differentiation between isomeric triantennary *N* ‐linked glycans by negative ion tandem mass spectrometry and confirmation of glycans containing galactose attached to the bisecting (*β* 1‐4‐GlcNAc) residue in *N* ‐glycans from IgG. Rapid Commun. Mass Spectrom. 22, 1047–1052. 10.1002/rcm.3470 18327885

[B26] He Y. Zhang M. Shan M. Zeng P. Li X. Hao C. (2018). Optimizing microwave-assisted hydrolysis conditions for monosaccharide composition analyses of different polysaccharides. Int. J. Biol. Macromol. 118, 327–332. 10.1016/j.ijbiomac.2018.06.077 29933001

[B27] Helm J. Grünwald-Gruber C. Thader A. Urteil J. Führer J. Stenitzer D. (2021). Bisecting lewis X in hybrid-type *N* -glycans of human brain revealed by deep structural glycomics. Anal. Chem. 93, 15175–15182. 10.1021/acs.analchem.1c03793 34723506 PMC8600501

[B28] Helm J. Hirtler L. Altmann F. (2022). Towards mapping of the human brain N-glycome with standardized graphitic carbon chromatography. Biomolecules 12, 85. 10.3390/biom12010085 35053234 PMC8774104

[B29] Hennig R. Cajic S. Borowiak M. Hoffmann M. Kottler R. Reichl U. (2016). Towards personalized diagnostics via longitudinal study of the human plasma N-glycome. Biochimica Biophysica Acta (BBA) - General Subj. 1860, 1728–1738. 10.1016/j.bbagen.2016.03.035 27038647

[B30] Hennig R. Rapp E. Kottler R. Cajic S. Borowiak M. Reichl U. (2015). N-glycosylation fingerprinting of viral glycoproteins by xCGE-LIF. Methods Mol. Biol. 1331, 123–143. 10.1007/978-1-4939-2874-3_8 26169738

[B31] Holland M. Yagi H. Takahashi N. Kato K. Savage C. O. S. Goodall D. M. (2006). Differential glycosylation of polyclonal IgG, IgG-Fc and IgG-Fab isolated from the sera of patients with ANCA-associated systemic vasculitis. Biochim. Biophys. Acta Gen. Subj. 1760, 669–677. 10.1016/j.bbagen.2005.11.021 16413679

[B32] Huang K. Li C. Zong G. Prabhu S. K. Chapla D. G. Moremen K. W. (2022). Site-selective sulfation of N-glycans by human GlcNAc-6-O-sulfotransferase 1 (CHST2) and chemoenzymatic synthesis of sulfated antibody glycoforms. Bioorg Chem. 128, 106070. 10.1016/j.bioorg.2022.106070 35939855 PMC9552261

[B33] Kenny D. T. Issa S. M. A. Karlsson N. G. (2011). Sulfate migration in oligosaccharides induced by negative ion mode ion trap collision‐induced dissociation. Rapid Commun. Mass Spectrom. 25, 2611–2618. 10.1002/rcm.5157 23657955

[B34] Khoo K. H. Yu S. Y. (2010). “Mass spectrometric analysis of sulfated N- and O-glycans,” in Methods in enzymology (Academic Press Inc.), 3–26. 10.1016/S0076-6879(10)78001-0 20816473

[B35] Kimura N. Ohmori K. Miyazaki K. Izawa M. Matsuzaki Y. Yasuda Y. (2007). Human B-lymphocytes express alpha2-6-sialylated 6-sulfo-N-acetyllactosamine serving as a preferred ligand for CD22/Siglec-2. J. Biol. Chem. 282, 32200–32207. 10.1074/jbc.M702341200 17728258

[B36] Kissel T. Ge C. Hafkenscheid L. Kwekkeboom J. C. Slot L. M. Cavallari M. (2022). Surface Ig variable domain glycosylation affects autoantigen binding and acts as threshold for human autoreactive B cell activation. Sci. Adv. 8, 1759. 10.1126/sciadv.abm1759 PMC8827743 35138894

[B37] Koers J. Derksen N. I. L. Falkenburg W. J. J. Ooijevaar-de Heer P. Nurmohamed M. T. Wolbink G. J. (2023a). Elevated Fab glycosylation of anti-hinge antibodies. Scand. J. Rheumatol. 52, 25–32. 10.1080/03009742.2021.1986959 34726124

[B38] Koers J. Sciarrillo R. Derksen N. I. L. Vletter E. M. Fillié-Grijpma Y. E. Raveling-Eelsing E. (2023b). Differences in IgG autoantibody Fab glycosylation across autoimmune diseases. J. Allergy Clin. Immunol. 151, 1646–1654. 10.1016/j.jaci.2022.10.035 36716825

[B39] Kolodziej A. Smalla K.-H. Richter S. Engler A. Pielot R. Dieterich D. C. (2016). High resolution quantitative synaptic proteome profiling of mouse brain regions after auditory discrimination learning. J. Vis. Exp., 54992. 10.3791/54992 28060347 PMC5226410

[B40] Kosuge H. Nagatoishi S. Kiyoshi M. Ishii-Watabe A. Terao Y. Ide T. (2022). Biophysical characterization of the contribution of the fab region to the IgG-FcγRIIIa interaction. Biochemistry 62, 262–269. 10.1021/acs.biochem.1c00832 35605982 PMC9850916

[B41] Lannergård J. Guss B. (2006). IdeE, an IgG-endopeptidase of *Streptococcus equi* ssp. *equi* . FEMS Microbiol. Lett. 262, 230–235. 10.1111/j.1574-6968.2006.00404.x 16923080

[B42] Lauc G. Vučković F. Bondt A. Pezer M. Wuhrer M. (2018). Trace N-glycans including sulphated species may originate from various plasma glycoproteins and not necessarily IgG. Nat. Commun. 9, 2916. 10.1038/s41467-018-05173-w 30046098 PMC6060135

[B43] Lefranc M.-P. (2011). IMGT, the international ImMunoGeneTics information system. Cold Spring Harb. Protoc. 2011, 595–603. 10.1101/pdb.top115 21632786

[B44] Lei M. Novotny M. V. Mechref Y. (2010). Sequential enrichment of sulfated glycans by strong anion-exchange chromatography prior to mass spectrometric measurements. J. Am. Soc. Mass Spectrom. 21, 348–357. 10.1016/j.jasms.2009.09.017 20022260

[B45] Leibiger H. Stner D. W. Stigler R.-D. Marx U. (1999). Variable domain-linked oligosaccharides of a human monoclonal IgG: structure and influence on antigen binding. Biochem. J. 338, 529–538. 10.1042/bj3380529 10024532 PMC1220082

[B46] MacAuley M. S. Crocker P. R. Paulson J. C. (2014). Siglec-mediated regulation of immune cell function in disease. Nat. Rev. Immunol. 14, 653–666. 10.1038/nri3737 25234143 PMC4191907

[B47] Mahan A. E. Tedesco J. Dionne K. Baruah K. Cheng H. D. De Jager P. L. (2015). A method for high-throughput, sensitive analysis of IgG Fc and Fab glycosylation by capillary electrophoresis. J. Immunol. Methods 417, 34–44. 10.1016/j.jim.2014.12.004 25523925 PMC5054724

[B48] Martini R. Xin Y. Schmitz B. Schachner M. (1992). The L2/HNK‐1 carbohydrate epitope is involved in the preferential outgrowth of motor neurons on ventral roots and motor nerves. Eur. J. Neurosci. 4, 628–639. 10.1111/j.1460-9568.1992.tb00171.x 12106326

[B49] Muñoz R. I. Kähne T. Herrera H. Rodríguez S. Guerra Ma. M. Vío K. (2019). The subcommissural organ and the Reissner fiber: old friends revisited. Cell Tissue Res. 375, 507–529. 10.1007/s00441-018-2917-8 30259139

[B50] Murphy K. Weaver C. (2018). Janeway immunologie. Berlin Heidelberg: Springer. 10.1007/978-3-662-56004-4

[B51] Neelamegham S. Aoki-Kinoshita K. Bolton E. Frank M. Lisacek F. Lütteke T. (2019). Updates to the symbol nomenclature for glycans guidelines. Glycobiology 29, 620–624. 10.1093/glycob/cwz045 31184695 PMC7335484

[B52] Ni W. Bones J. Karger B. L. (2013). In-depth characterization of N-linked oligosaccharides using fluoride-mediated negative ion microfluidic chip LC–MS. Anal. Chem. 85, 3127–3135. 10.1021/ac3031898 23398125 PMC3604099

[B53] Nimmerjahn F. Vidarsson G. Cragg M. S. (2023). Effect of posttranslational modifications and subclass on IgG activity: from immunity to immunotherapy. Nat. Immunol. 24, 1244–1255. 10.1038/s41590-023-01544-8 37414906

[B54] Nitschke L. (2009). CD22 and Siglec‐G: B‐cell inhibitory receptors with distinct functions. Immunol. Rev. 230, 128–143. 10.1111/j.1600-065X.2009.00801.x 19594633

[B55] Plomp R. de Haan N. Bondt A. Murli J. Dotz V. Wuhrer M. (2018). Comparative glycomics of immunoglobulin A and G from saliva and plasma reveals biomarker potential. Front. Immunol. 9, 2436. 10.3389/fimmu.2018.02436 30405629 PMC6206042

[B56] Rees A. R. (2014). The antibody molecule. Oxford: Oxford University Press. Available online at: http://gbv.eblib.com/patron/FullRecord.aspx?p=1817918.

[B57] Rombouts Y. Willemze A. Van Beers J. J. B. C. Shi J. Kerkman P. F. Van Toorn L. (2016). Extensive glycosylation of ACPA-IgG variable domains modulates binding to citrullinated antigens in rheumatoid arthritis. Ann. Rheum. Dis. 75, 578–585. 10.1136/annrheumdis-2014-206598 25587188

[B58] Rosen S. D. (1999). Endothelial ligands for L-selectin: from lymphocyte recirculation to allograft rejection. Am. J. Pathol. 155, 1013–1020. 10.1016/S0002-9440(10)65201-7 10514381 PMC1867022

[B59] Scott H. Panin V. M. (2014). N-glycosylation in regulation of the nervous system. Adv. Neurobiol. 9, 367–394. 10.1007/978-1-4939-1154-7_17 25151388 PMC4476505

[B60] Seeling M. Brückner C. Nimmerjahn F. (2017). Differential antibody glycosylation in autoimmunity: sweet biomarker or modulator of disease activity? Nat. Rev. Rheumatol. 13, 621–630. 10.1038/nrrheum.2017.146 28905852

[B61] She Y. M. Farnsworth A. Li X. Cyr T. D. (2017). Topological N-glycosylation and site-specific N-glycan sulfation of influenza proteins in the highly expressed H1N1 candidate vaccines. Sci. Rep. 7, 10232. 10.1038/s41598-017-10714-2 28860626 PMC5579265

[B62] She Y. M. Li X. Cyr T. D. (2019). Remarkable structural diversity of N-glycan sulfation on influenza vaccines. Anal. Chem. 91, 5083–5090. 10.1021/acs.analchem.8b05372 30908021

[B63] Shkunnikova S. Mijakovac A. Sironic L. Hanic M. Lauc G. Kavur M. M. (2023). IgG glycans in health and disease: prediction, intervention, prognosis, and therapy. Biotechnol. Adv. 67, 108169. 10.1016/j.biotechadv.2023.108169 37207876

[B64] Šoić D. Mlinarić Z. Lauc G. Gornik O. Novokmet M. Keser T. (2022). In a pursuit of optimal glycan fluorescent label for negative MS mode for high-throughput N-glycan analysis. Front. Chem. 10, 999770. 10.3389/fchem.2022.999770 36262345 PMC9574008

[B65] Stavenhagen K. Plomp R. Wuhrer M. (2015). Site-specific protein N- and O-glycosylation analysis by a C18-porous graphitized carbon-liquid chromatography-electrospray ionization mass spectrometry approach using pronase treated glycopeptides. Anal. Chem. 87, 11691–11699. 10.1021/acs.analchem.5b02366 26536155

[B66] Takegawa Y. Deguchi K. Nakagawa H. Nishimura S.-I. (2005). Structural analysis of an N-glycan with “β1−4 bisecting branch” from human serum IgG by negative-ion MS ^n^ spectral matching and exoglycosidase digestion. Anal. Chem. 77, 6062–6068. 10.1021/ac050843e 16159142

[B67] Thomsson K. A. Bäckström M. Holmén Larsson J. M. Hansson G. C. Karlsson H. (2010). Enhanced detection of sialylated and sulfated glycans with negative Ion mode nanoliquid chromatography/mass spectrometry at high pH. Anal. Chem. 82, 1470–1477. 10.1021/ac902602e 20092260

[B68] Toyoda M. Narimatsu H. Kameyama A. (2009). Enrichment method of sulfated glycopeptides by a sulfate emerging and ion exchange chromatography. Anal. Chem. 81, 6140–6147. 10.1021/ac900592t 19572564

[B69] van de Bovenkamp F. S. Hafkenscheid L. Rispens T. Rombouts Y. (2016). The emerging importance of IgG fab glycosylation in immunity. J. Immunol. 196, 1435–1441. 10.4049/jimmunol.1502136 26851295

[B70] Vidarsson G. Dekkers G. Rispens T. (2014). IgG subclasses and allotypes: from structure to effector functions. Front. Immunol. 5, 520. 10.3389/fimmu.2014.00520 25368619 PMC4202688

[B71] Vletter E. M. Koning M. T. Scherer H. U. Veelken H. Toes R. E. M. (2020). A comparison of immunoglobulin variable region N-linked glycosylation in healthy donors, autoimmune disease and lymphoma. Front. Immunol. 11, 241. 10.3389/fimmu.2020.00241 32133009 PMC7040075

[B72] Volkov M. Brinkhaus M. van Schie K. A. Bondt A. Kissel T. van der Kooi E. J. (2023). IgG fab glycans hinder FcRn-mediated placental transport. J. Immunol. 210, 158–167. 10.4049/jimmunol.2200438 36480251

[B73] Walker J. A. Smith K. G. C. (2008). CD22: an inhibitory enigma. Immunology 123, 314–325. 10.1111/j.1365-2567.2007.02752.x 18067554 PMC2433339

[B74] Walsh G. Walsh E. (2022). Biopharmaceutical benchmarks 2022. Nat. Biotechnol. 40, 1722–1760. 10.1038/s41587-022-01582-x 36471135 PMC9735008

[B75] Wang J. R. Gao W. N. Grimm R. Jiang S. Liang Y. Ye H. (2017). A method to identify trace sulfated IgG N-glycans as biomarkers for rheumatoid arthritis. Nat. Commun. 8, 631. 10.1038/s41467-017-00662-w 28931878 PMC5606999

[B76] Wang J. R. Gao W. N. Grimm R. Jiang S. Liang Y. Ye H. (2018). Reply to ‘Trace N-glycans including sulphated species may originate from various plasma glycoproteins and not necessarily IgG. Nat. Commun. 9, 2915. 10.1038/s41467-018-05082-y 30046037 PMC6060093

[B77] Wang Y. Tan J. Sutton-Smith M. Ditto D. Panico M. Campbell R. M. (2001). Modeling human congenital disorder of glycosylation type IIa in the mouse: conservation of asparagine-linked glycan-dependent functions in mammalian physiology and insights into disease pathogenesis. Glycobiology 11, 1051–1070. 10.1093/glycob/11.12.1051 11805078

[B78] Wedepohl S. Kaup M. Riese S. B. Berger M. Dernedde J. Tauber R. (2010). N-glycan analysis of recombinant l-selectin reveals sulfated GalNAc and GalNAc-GalNAc motifs. J. Proteome Res. 9, 3403–3411. 10.1021/pr100170c 20469932

[B79] Weiss M. Ott D. Karagiannis T. Weishaupt M. Niemietz M. Eller S. (2020). Efficient chemoenzymatic synthesis of N-glycans with a β1,4-galactosylated bisecting GlcNAc motif. ChemBioChem 21, 3212–3215. 10.1002/cbic.202000268 32597008 PMC7723014

[B80] Xu Q. Deng X. Zhang B. Zhao C. Huang T. Zhang Y. (2021). A study of the possible role of Fab-glycosylated IgG in tumor immunity. Cancer Immunol. Immunother. 70, 1841–1851. 10.1007/s00262-020-02809-z 33388997 PMC10992005

[B81] Yagi H. Takahashi N. Yamaguchi Y. Kimura N. Uchimura K. Kannagi R. (2005). Development of structural analysis of sulfated N-glycans by multidimensional high performance liquid chromatography mapping methods. Glycobiology 15, 1051–1060. 10.1093/glycob/cwi092 15958418

[B82] Yamada K. Suzuki K. Hirohata Y. Kinoshita M. (2020). Analysis of minor acidic N-glycans in human serum. J. Proteome Res. 19, 3033–3043. 10.1021/acs.jproteome.0c00079 32436713

[B83] Yoshimura T. Hayashi A. Handa-Narumi M. Yagi H. Ohno N. Koike T. (2017). GlcNAc6ST-1 regulates sulfation of N-glycans and myelination in the peripheral nervous system. Sci. Rep. 7, 42257. 10.1038/srep42257 28186137 PMC5301494

[B84] Zuniga-Banuelos F. J. Hoffmann M. Reichl U. Rapp E. (2025a). New avenues for human blood plasma biomarker discovery via improved in-depth analysis of the low-abundant N–glycoproteome. Engineering. 10.1016/j.eng.2024.11.039

[B85] Zuniga-Banuelos F. J. Lemke G. Hoffmann M. Reichl U. Rapp E. (2025b). Immunoglobulin A carries sulfated and O-acetylated N-glycans primarily at the tailpiece site -strategies for site-specific N-glycan identification. Front. Mol. Biosci. 12, 1595173. 10.3389/fmolb.2025.1595173 PMC12353717 40821702

