# Supplementary material for: Raider of the lost N-glycans – Localizing rare and frequently overlooked IgG N-glycans with sulfation or bisecting LacNAc
Source: Front Mol Biosci. 2025 Jul 9;12:1593708. doi: 10.3389/fmolb.2025.1593708 (PMC12283335; doi:10.3389/fmolb.2025.1593708)
Supplement: Supplementary file 1 [file DataSheet1.pdf]

—*Supplementary Material*—

Raider of the lost *N*-glycans – Localizing rare and frequently overlooked IgG *N*-glycans with sulfation or bisecting LacNAc

Robert Burock[1†], Léa Chuzel[2†], Thilo Kähne[3†], Udo Reichl[4,5†], Erdmann Rapp[1,4†], René Hennig[1†*]

[1] glyXera GmbH, Magdeburg, Germany
[2] New England Biolabs, Ipswich, MA, United States
[3] Institute of Experimental Internal Medicine, Medical School, Otto-von-Guericke-University Magdeburg, Magdeburg, Germany
[4] Bioprocess Engineering, Max-Planck-Institute for Dynamics of Complex Technical Systems, Magdeburg, Germany
[5] Bioprocess Engineering, Otto-von-Guericke-University Magdeburg, Magdeburg, Germany

[†] ORCID°IDs

Robert Burock	0000-0001-6372-2770
Léa Chuzel	0000-0001-7185-5872
Thilo Kähne	0000-0001-7250-621X
Udo Reichl	0000-0001-6538-1332
Erdmann Rapp	0000-0001-6618-2626
René Hennig	0000-0001-9172-7982

Table of Contents

1 *N*-glycan analysis of additional blood plasma, IgG and Fab samples 4

Supplementary Figure 1: Tracking FA2Su1G2S2(6,6) in *N*-glycan fingerprints of blood plasma, intact IgG, Fab, and Fc from donor 2. 5

Supplementary Figure 2: Tracking FA2Su1G2S2(6,6) in *N*-glycan fingerprints of blood plasma, intact IgG, Fab, and Fc from donor 3. 6

Supplementary Figure 3: Tracking FA2Su1G2S2(6,6) in *N*-glycan fingerprints of frozen normal control plasma batch 0009-52FCP and intact IgG, Fab, and Fc derived thereof. 7

Supplementary Figure 4: Tracking FA2Su1G2S2(6,6) in *N*-glycan fingerprints of frozen normal control plasma batch 0012-52FCP and intact IgG, Fab, and Fc derived thereof. 8

Supplementary Figure 5: Tracking FA2Su1G2S2(6,6) in the *N*-glycan fingerprints of commercial IgG batch IG1802-R22 and Fab and Fc derived thereof. 9

Supplementary Figure 6: Tracking FA2Su1G2S2(6,6) in the *N*-glycan fingerprints of commercial IgG batch IG2017-01 and Fab and Fc derived thereof. 10

Supplementary Figure 7: Sulfated *N*-glycans in the *N*-glycan fingerprints of commercial Fab and $F(ab')_2$ samples. 11

2 Estimating the Fab glycosylation frequency from the FA2Su1G2S2(6,6) peak 12

Supplementary Table 1: Fab glycosylation frequencies of all samples analyzed during this work. 13

3 Identification of glycoprotein impurities in intact IgG, the Fab and the Fc fraction 14

Supplementary Table 2: Top 10 protein hits from the intact IgG obtained from blood plasma of donor 1 14

Supplementary Table 3: Top 10 protein hits from the Fab fraction obtained from blood plasma of donor 1 15

Supplementary Table 4: Top 10 protein hits from the Fc fraction obtained from blood plasma of donor 1 15

4 Identification of proteins in bands from non-reducing SDS-PAGE 16

Supplementary Table 5: Top 10 protein hits from band 4-1 16

Supplementary Table 6: Top 10 protein hits from band 4-2 17

Supplementary Table 7: Top 10 protein hits from band 4-3 17

Supplementary Table 8: Top 10 protein hits from band 4-4 18

Supplementary Table 9: Top 10 protein hits from band 4-5 18

Supplementary Table 10: Top 10 protein hits from band 4-6 19

Supplementary Table 11: Top 10 protein hits from band 5-1 20

Supplementary Table 12: Top 10 protein hits from band 5-2 20

Supplementary Table 13: Top 10 protein hits from band 5-3 21
Supplementary Table 14: Top 10 protein hits from band 5-4 21
Supplementary Table 15: Top 10 protein hits from band 5-5 22
Supplementary Table 16: Top 10 protein hits from band 5-6 22
Supplementary Table 17: Top 10 protein hits from band 6-1 23
Supplementary Table 18: Seven protein hits from band 6-2 23

5 Exoglycosidase digests and EDGE-profiling of blood plasma, intact IgG, Fc and IgA 25

Supplementary Figure 8: Exoglycosidase digests and EDGE-profiling of blood plasma-derived *N*-glycans. 27
Supplementary Figure 9: Exoglycosidase digests and EDGE-profiling of intact IgG-derived *N*-glycans. 29
Supplementary Figure 10: Exoglycosidase digests and EDGE-profiling of Fc-derived *N*-glycans. 31
Supplementary Figure 11: Exoglycosidase digests and EDGE-profiling of IgA-derived *N*-glycans. 33
Supplementary Figure 12: Identification of an unknown peak appearing in EDGE-profiling as a non-glycan impurity. 34

6 Exoglycosidase digests to verify the identification of FA2BG3 37

Supplementary Figure 13: Additional exoglycosidase digests of desialylated Fab-derived *N*-glycans. 39
Supplementary Figure 14: Additional MANase and GlcNAcase digests of Fab-derived *N*-glycans after SiaA and GALase digests. 40

7 Relative quantification of Fab-derived *N*-glycans 41

Supplementary Table 19: Relative quantification data of Fab-derived *N*-glycans. 41

8 HILIC-UPLC-FLD analysis of human IgG-derived *N*-glycans 48

Supplementary Figure 15: HILIC-UPLC-FLD chromatogram of IgG-derived *N*-glycans 48

9 Remarks on the applicability of EDGE-profiling to unreleased *N*-glycans 49

10 Supplementary references 49

1 *N*-glycan analysis of additional blood plasma, IgG and Fab samples

We wanted to make sure that the observation made can be generalized. Therefore, we analyzed a number of samples of different origins. In total, we looked at:

- IgG isolated from three blood plasma samples of individual donors that were part of a VisuCon™ normal donor set (Lot: NDS-0013, Affinity Biologicals Inc., Ancaster, Canada), including plasma of donor 1 (ID: ND105; LOT: 673760816), donor 2 (ID: ND96; LOT: 667210816), and donor 3 (ID: ND116; LOT: 667600816)
- IgG isolated from two batches frozen normal control plasma pools (VisuCon™-F frozen normal control plasma, batches 0009-52FCP and 0012-52FCP, Affinity Biologicals Inc., Ancaster, Canada)
- Two commercial IgG samples (batches IG1802-R22 and IG2017-01, Athens Research & Technology, Georgia, USA)
- Commercial Fab and $F(ab')_2$ preparations (Athens Research & Technology, Georgia, USA).

Intact IgG was captured by affinity chromatography using CaptureSelect™ Fc affinity matrix (ThermoFisher Scientific, Darmstadt, Germany), or captured and proteolytically separated into Fab and Fc using IdeZ (New England Biolabs Inc., Ipswich, USA). *N*-glycans of all samples were released with PNGase F, fluorescently labeled with APTS, and cleaned up via HILIC-SPE using the glyXprep™ kit. Labeled *N*-glycans were analyzed on a glyXboxCE™ system and glycan data were analyzed using the glycoanalysis software glyXtoolCE™ (all glyXera GmbH, Magdeburg, Germany). Throughout the main manuscript, data of donor 1 is shown. Here, xCGE-LIF-based fingerprints (i.e., migration time aligned electropherograms) of the other samples are shown.

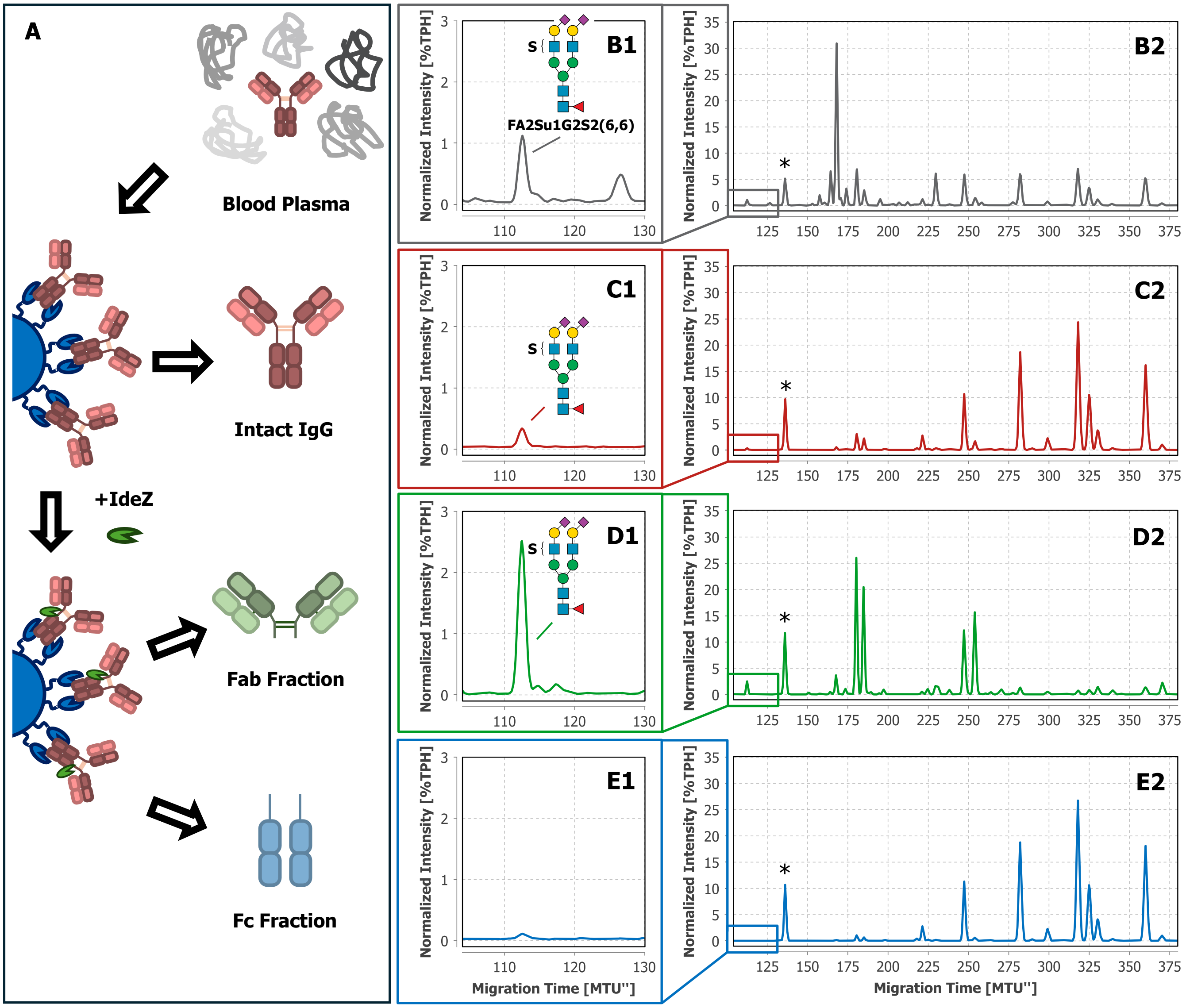

Supplementary Figure 1: Tracking FA2Su1G2S2(6,6) in *N*-glycan fingerprints of blood plasma, intact IgG, Fab, and Fc from donor 2. The workflow starts with blood plasma as a complex glycoprotein mixture (**A**). From this mixture, either intact IgG was selectively captured by affinity chromatography and eluted, or IgG was selectively captured, cleaved on-bead by IdeZ, and separated into Fab and Fc containing fractions. In the xCGE-LIF-based *N*-glycan fingerprints of blood plasma (**B1, B2**), intact IgG (**C1, C2**), and the Fab fraction (**D1, D2**) a FA2Su1G2S2(6,6) peak is found at ~112 MTU", but not in the fingerprint of the Fc fraction (**E1, E2**). * Migration time alignment standard. Blue squares: GlcNAc. Red triangles: fucose. Green circles: mannose. Yellow circles: galactose. Purple diamonds: *N*-acetyl neuraminic acid (Neu5Ac, α2,6-linked when tilted to the right). S indicates a sulfate group.

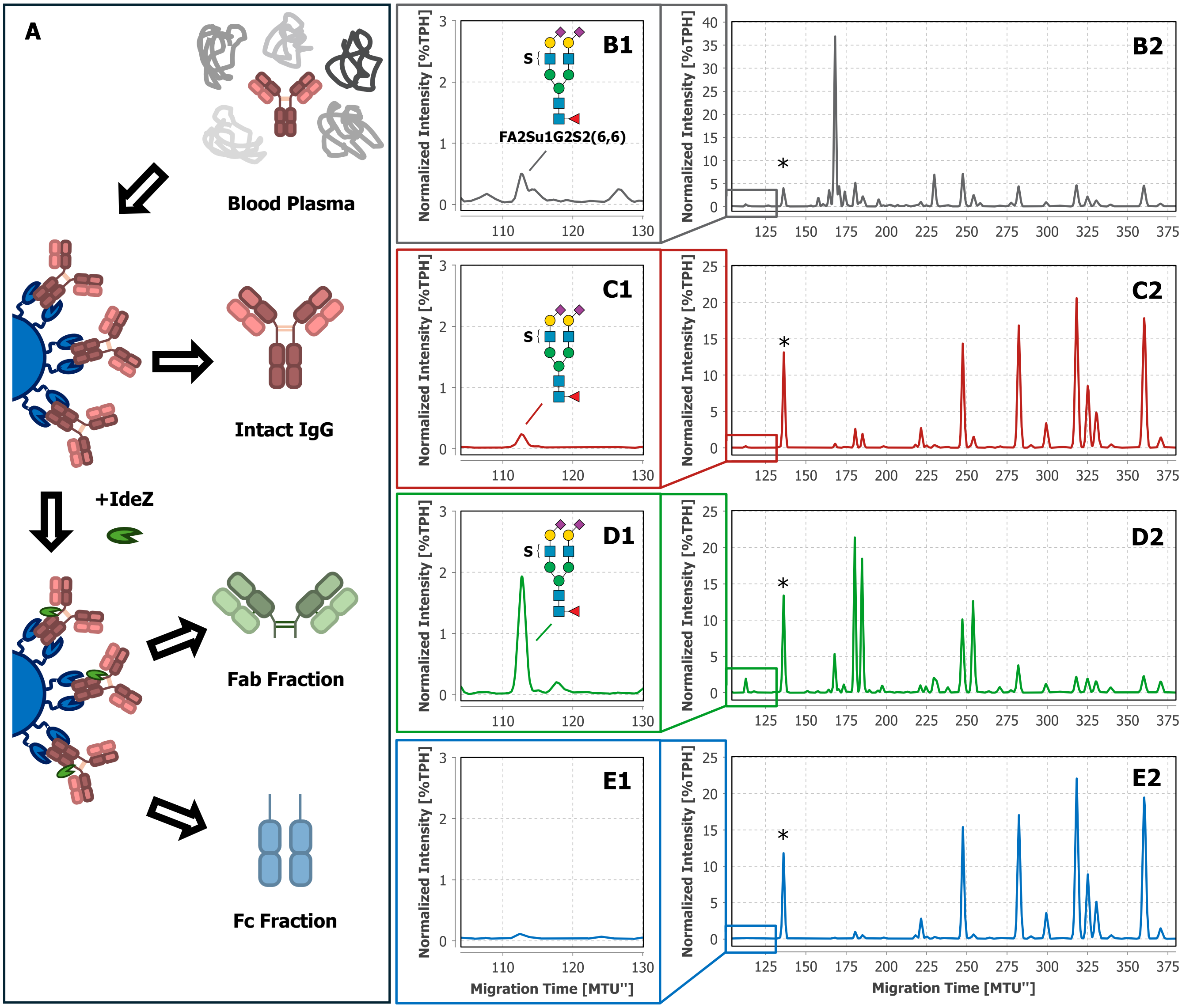

Supplementary Figure 2: Tracking FA2Su1G2S2(6,6) in *N*-glycan fingerprints of blood plasma, intact IgG, Fab, and Fc from donor 3. The workflow starts with blood plasma as a complex glycoprotein mixture (**A**). From this mixture, either intact IgG was selectively captured by affinity chromatography and eluted, or IgG was selectively captured, cleaved on-bead by IdeZ, and separated into Fab and Fc containing fractions. In the xCGE-LIF-based *N*-glycan fingerprints of blood plasma (**B1, B2**), intact IgG (**C1, C2**), and the Fab fraction (**D1, D2**) a FA2Su1G2S2(6,6) peak is found at ~112 MTU'', but not in the fingerprint of the Fc fraction (**E1, E2**). * Migration time alignment standard. Blue squares: GlcNAc. Red triangles: fucose. Green circles: mannose. Yellow circles: galactose. Purple diamonds: Neu5Ac (α2,6-linked when tilted to the right). S indicates a sulfate group.

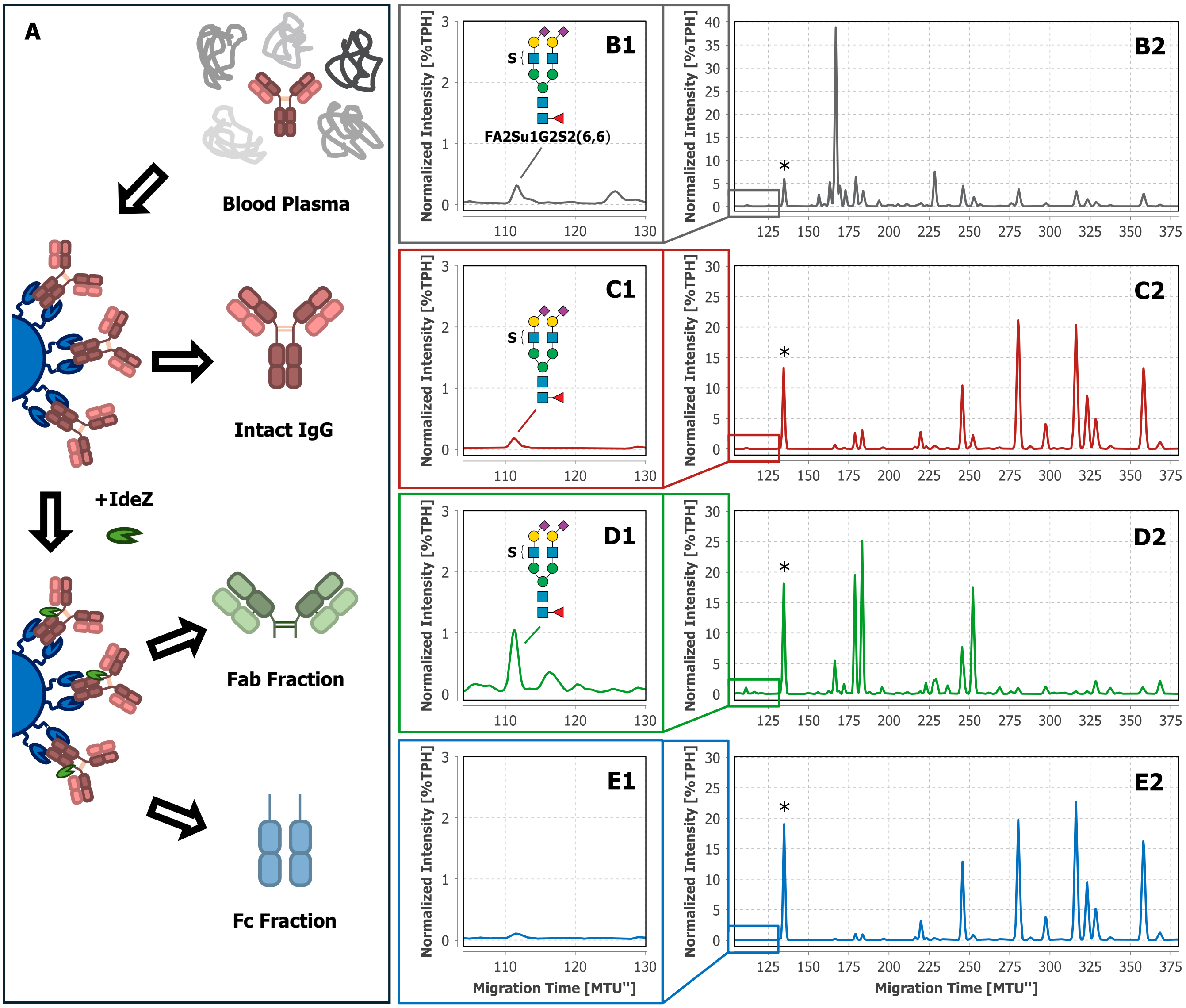

Supplementary Figure 3: Tracking FA2Su1G2S2(6,6) in *N*-glycan fingerprints of frozen normal control plasma batch 0009-52FCP and intact IgG, Fab, and Fc derived thereof. The workflow starts with VisuCon™-F frozen normal control plasma batch 0009-52FCP (Affinity Biologicals Inc., Ancaster, Canada) as a complex glycoprotein mixture (**A**) that was pooled from at least 20 donors. From this mixture, either intact IgG was selectively captured by affinity chromatography and eluted, or IgG was selectively captured, cleaved on-bead by IdeZ, and separated into Fab and Fc containing fractions. In the xCGE-LIF-based *N*-glycan fingerprints of the blood plasma pool (**B1, B2**), intact IgG (**C1, C2**), and the Fab fraction (**D1, D2**) a FA2Su1G2S2(6,6) peak is found at ~112 MTU", but not in the fingerprint of the Fc fraction (**E1, E2**). * Migration time alignment standard. Blue squares: GlcNAc. Red triangles: fucose. Green circles: mannose. Yellow circles: galactose. Purple diamonds: Neu5Ac (α2,6-linked when tilted to the right). S indicates a sulfate group.

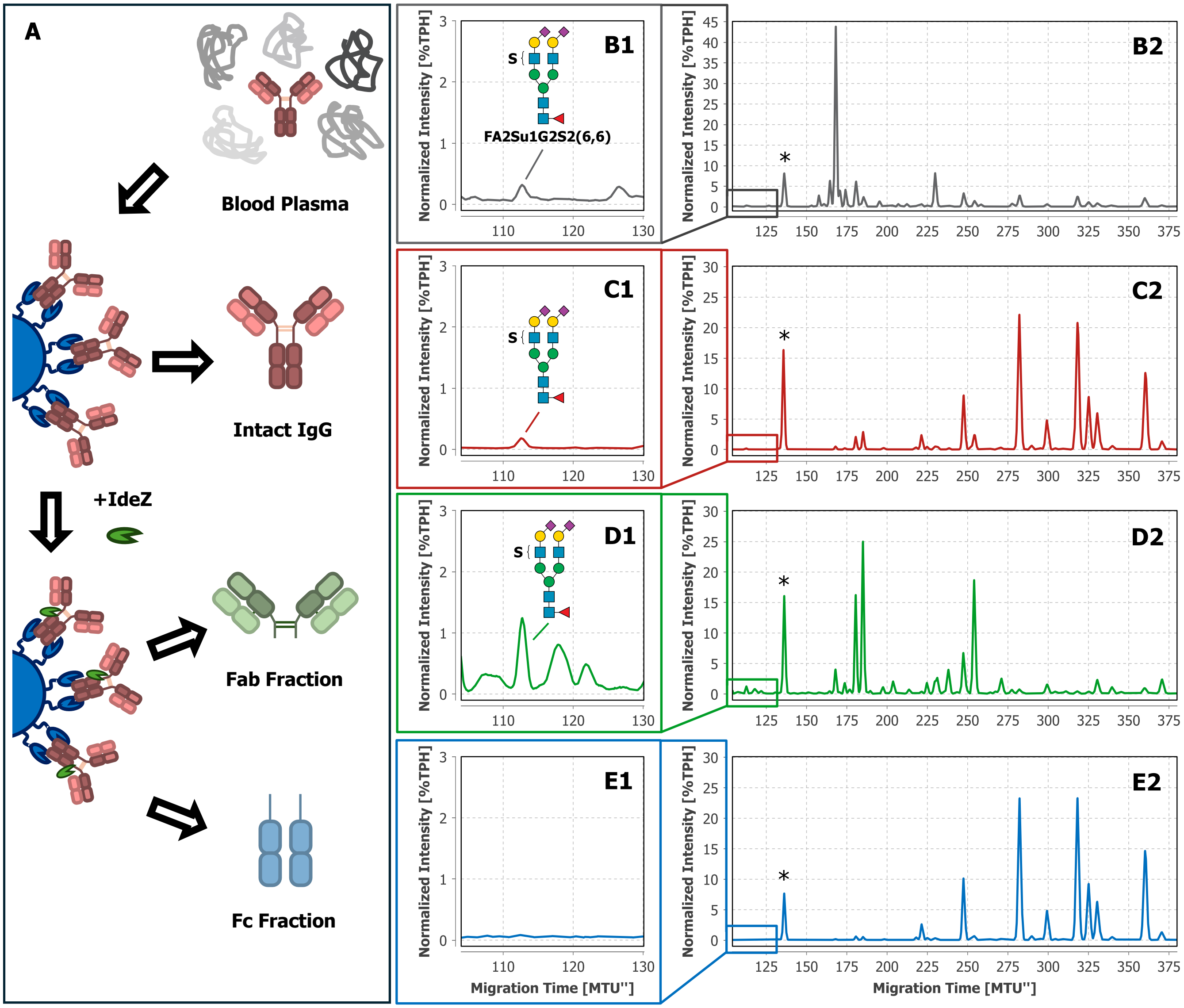

Supplementary Figure 4: Tracking FA2Su1G2S2(6,6) in *N*-glycan fingerprints of frozen normal control plasma batch 0012-52FCP and intact IgG, Fab, and Fc derived thereof. The workflow starts with VisuCon™-F frozen normal control plasma batch 0012-52FCP (Affinity Biologicals Inc., Ancaster, Canada) as a complex glycoprotein mixture (**A**) that was pooled from at least 20 donors. From this mixture, either intact IgG was selectively captured by affinity chromatography and eluted, or IgG was selectively captured, cleaved on-bead by IdeZ, and separated into Fab and Fc containing fractions. In the xCGE-LIF-based *N*-glycan fingerprints of the blood plasma pool (**B1, B2**), intact IgG (**C1, C2**), and the Fab fraction (**D1, D2**) a FA2Su1G2S2(6,6) peak is found at ~112 MTU", but not in the fingerprint of the Fc fraction (**E1, E2**). * Migration time alignment standard. Blue squares: GlcNAc. Red triangles: fucose. Green circles: mannose. Yellow circles: galactose. Purple diamonds: Neu5Ac (α2,6-linked when tilted to the right). S indicates a sulfate group.

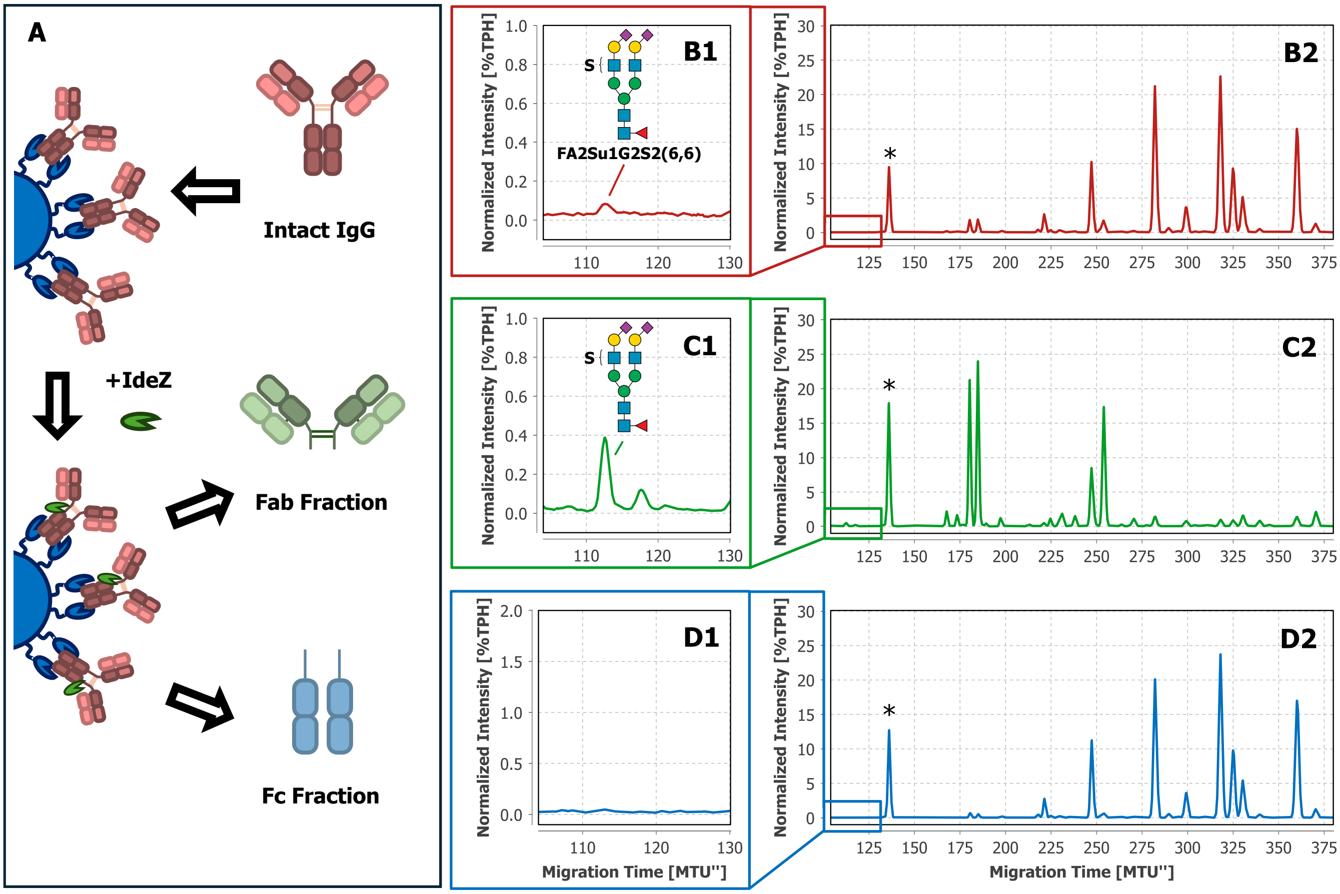

Supplementary Figure 5: Tracking FA2Su1G2S2(6,6) in the *N*-glycan fingerprints of commercial IgG batch IG1802-R22 and Fab and Fc derived thereof. The workflow (**A**) starts with a bought human IgG preparation (Athens Research & Technology, Georgia, USA). IgG was purified again by selective capturing using affinity chromatography, or selectively captured, cleaved on-bead by IdeZ, and separated into Fab and Fc containing fractions. In the xCGE-LIF-based *N*-glycan fingerprints (i.e., aligned electropherograms) of intact IgG (**B1, B2**) and the Fab fraction (**C1, C2**) a FA2Su1G2S2(6,6) peak is found at ~112 MTU'', but not in the fingerprint of the Fc fraction (**D1, D2**). * Migration time alignment standard. Blue squares: GlcNAc. Red triangles: fucose. Green circles: mannose. Yellow circles: galactose. Purple diamonds: Neu5Ac (α2,6-linked when tilted to the right). S indicates a sulfate group.

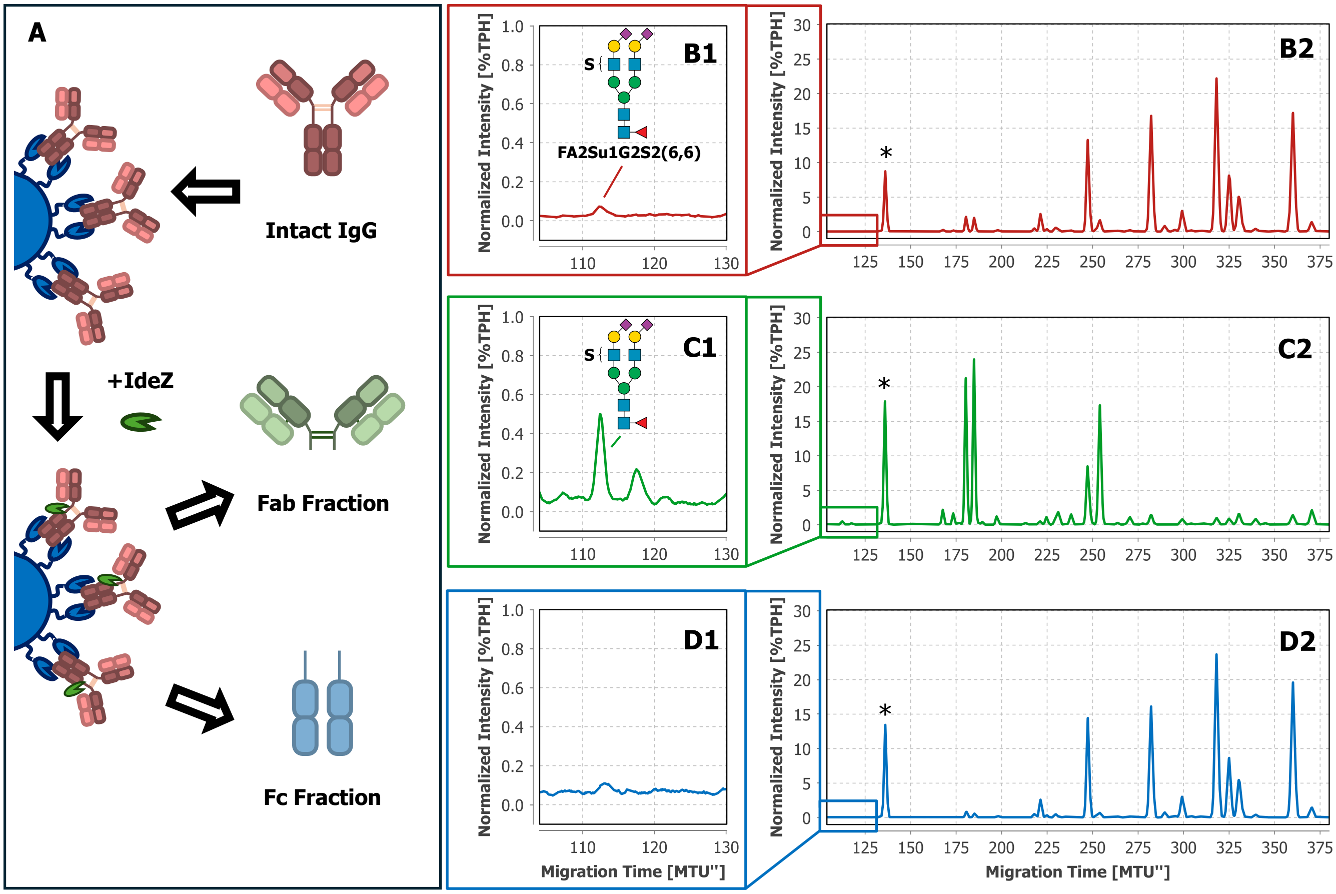

Supplementary Figure 6: Tracking FA2Su1G2S2(6,6) in the *N*-glycan fingerprints of commercial IgG batch IG2017-01 and Fab and Fc derived thereof. The workflow (**A**) starts with a bought human IgG preparation (Athens Research & Technology, Georgia, USA). IgG was purified again by selective capturing using affinity chromatography, or selectively captured, cleaved on-bead by IdeZ, and separated into Fab and Fc containing fractions. In the xCGE-LIF-based *N*-glycan fingerprints (i.e., aligned electropherograms) of intact IgG (**B1, B2**) and the Fab fraction (**C1, C2**) a FA2Su1G2S2(6,6) peak is found at ~112 MTU'', but not in the fingerprint of the Fc fraction (**D1, D2**). * Migration time alignment standard. Blue squares: GlcNAc. Red triangles: fucose. Green circles: mannose. Yellow circles: galactose. Purple diamonds: Neu5Ac (α2,6-linked when tilted to the right). S indicates a sulfate group.

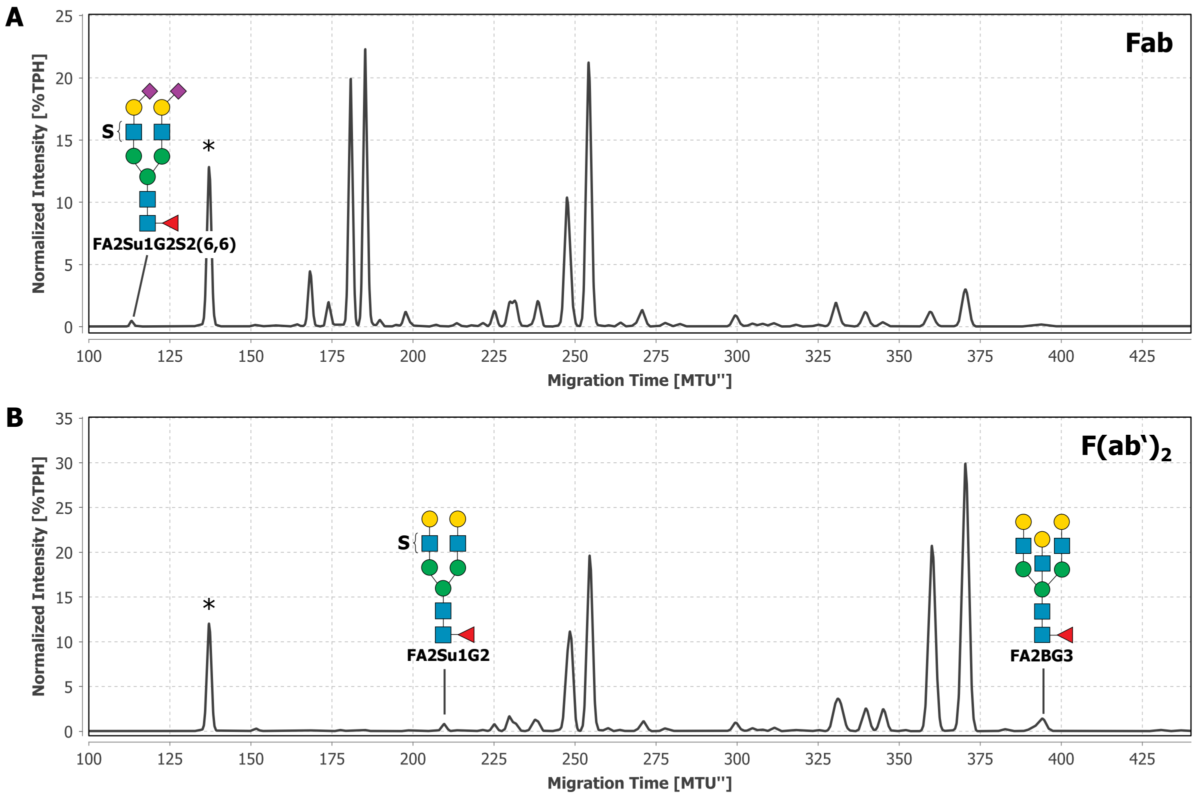

Supplementary Figure 7: Sulfated *N*-glycans in the *N*-glycan fingerprints of commercial Fab and F(ab')$_2$ samples. *N*-glycans of a Fab and a F(ab')$_2$ preparation (Athens Research & Technology, Georgia, USA) were analyzed without prior sample preparation like, e.g., a repurification. *N*-glycans of the Fab (**A**) are still sialylated, so that FA2Su1G2S2(6,6) at ~112 MTU'' can be identified. *N*-glycans of the F(ab')$_2$ preparation (**B**) show a significant loss of sialic acids, even though the two samples were handled identically. This might happen due to inappropriate conditions during the sample preparation, storage, or shipment, before the samples were received. Therefore, FA2Su1G2 at ~209 MTU'' is detected. Also, an initially unidentified peak at ~395 MTU'' was detected, that was identified as FA2BG3, a *N*-glycan with a bisecting *N*-acetyllactosamin (LacNAc, **Suppl. Chapter 6**). * Migration time alignment standard. Blue squares: GlcNAc. Red triangles: fucose. Green circles: mannose. Yellow circles: galactose. Purple diamonds: Neu5Ac (α2,6-linked when tilted to the right). S indicates a sulfate group.

2 Estimating the Fab glycosylation frequency from the FA2Su1G2S2(6,6) peak

xCGE-LIF-based *N*-glycan analysis is a powerful tool to analyze *N*-glycans after release from the respective glycoprotein. But a minor downside of this powerful analysis technique is that information related to the protein portion is usually lost. This means that no information can be directly obtained about macroheterogeneity, which is the presence or absence (or therefore, degree) of *N*-glycosylation at a given glycosylation site. Information about microheterogeneity, i.e. the diversity of *N*-glycan structures at a glycosylation site, can only be obtained if the analyzed glycoprotein has a single glycosylation site. However, the presented set-up of sample preparation and analysis allows the estimation of the Fab *N*-glycosylation frequency. This will represent only an average value, because a Fab might not only acquire one, but maybe two, three or more glycosylation sites.

Assuming that the quantity of FA2Su1G2S2(6,6) found on intact IgG is equal to the sum of FA2Su1G2S2(6,6) quantities found on Fab and Fc, we start with the equation:

$$TPH_{\mathrm{FA2Su1G2S2(6,6)};IgG} \times \left(f_{Fc} + f_{Fab}\right) = TPH_{\mathrm{FA2Su1G2S2(6,6)};Fab} \times f_{Fab} + TPH_{\mathrm{FA2Su1G2S2(6,6)};Fc} \times f_{Fc} \quad \textbf{(1)}$$

Where

$TPH_{\mathrm{FA2Su1G2S2(6,6)}}$ describes the determined abundance of the FA2Su1G2S2(6,6) peak of a certain sample (i.e., intact IgG, Fab or Fc), expressed in % of total peak height.

$$TPH_{\mathrm{FA2Su1G2S2(6,6)};IgG} = 0.24\ \%TPH$$

$$TPH_{\mathrm{FA2Su1G2S2(6,6)};Fab} = 1.82\ \%TPH$$

$$TPH_{\mathrm{FA2Su1G2S2(6,6)};Fc} = 0.00\ \%TPH$$

f Describes the glycosylation frequency of the Fab or Fc in %

$$f_{Fc} = 100\ \%\ \text{(assumed for all samples [1,2])}$$

Implementing the values for $TPH_{\mathrm{FA2Su1G2S2(6,6)},Fc}$ simplifies equation (1):

$$TPH_{\mathrm{FA2Su1G2S2(6,6)};IgG} \times (f_{Fc} + f_{Fab}) = TPH_{\mathrm{FA2Su1G2S2(6,6)};Fab} \times f_{Fab} \quad \textbf{(2)}$$

Rearranging the equation for f_{Fab} results in

$$f_{Fab} = \frac{TPH_{\mathrm{FA2Su1G2S2(6,6)};IgG}}{TPH_{\mathrm{FA2Su1G2S2(6,6)};Fab} - TPH_{\mathrm{FA2Su1G2S2(6,6)};IgG}} \times f_{Fc} \quad \textbf{(3)}$$

Inserting the values gives:

$$f_{Fab} = \frac{0.24\ \%TPH}{1.82\ \%TPH - 0.24\ \%TPH} \times 100\ \% \qquad \textbf{(4)}$$

$$f_{Fab} \approx 15.2\ \% \qquad \textbf{(5)}$$

Inserting the determined values for all analyzed samples, Fab *N*-glycosylation frequencies between 14.2%TPH and 25.8%TPH were estimated (**Suppl. Table 1**)

Supplementary Table 1:Fab glycosylation frequencies of all samples analyzed during this work. Using the above-described approach, the glycosylation frequencies of the following samples were estimated:

Sample	$TPH_{FA2Su1G2S2(6,6);IgG}$ [%TPH]	$TPH_{FA2Su1G2S2(6,6);Fab}$ [%TPH]	$TPH_{FA2Su1G2S2(6,6);Fc}$ [%TPH]	f_{Fab} [%TPH]
Donor 1 (ID: ND105; LOT: 673760816)	0.24	1.82	0	15.2
Donor 2 (ID: ND96; LOT: 667210816)	0.34	2.51	0	15.7
Donor 3 (ID: ND116; LOT: 667600816)	0.24	1.93	0	14.2
Frozen Normal Control Plasma (VisuCon™-F, Batch 0009-52FCP)	0.18	1.06	0	20.5
Frozen Normal Control Plasma (VisuCon™-F, Batch 0012-52FCP)	0.18	1.24	0	17.0
Intact IgG (Athens Research & Technology, Batch IG1802-R22)	0.08	0.39	0	25.8
Intact IgG (Athens Research & Technology, Batch IG2017-01)	0.07	0.5	0	16.3

3 Identification of glycoprotein impurities in intact IgG, the Fab and the Fc fraction

Intact IgG, Fab and Fc in-solution samples obtained from the blood plasma of donor 1 were analyzed by LC-MS/MS-based proteomics to assess the presence of (glyco-)protein impurities. Sample preparation was performed as described by Kolodziej et al. [3] and analyses were performed on a hybrid dual pressure linear ion trap/orbitrap mass spectrometer (LTQ Orbitrap Velos Pro, Thermo Scientific, San Jose, CA, USA) equipped with an Ultimate 3000-nLC Ultra HPLC (Thermo Scientific, San Jose, CA, USA) and a 200 cm µPAC™ RP C18-csA column (PharmaFluidics, Ghent, Belgium). Data processing and protein identification were performed with *de novo* sequencing algorithms of PEAKS Studio 8.0 (Bioinformatics Solutions Inc., Waterloo, Canada), using the human SwissProt database. After exclusion of ubiquitous and commonly identified skin-derived proteins like keratin, the top 10 hits of each sample are presented in the following tables. In all descriptions, the redundant entry " OS=Homo sapiens OX=9606" was removed for better readability.

Supplementary Table 2:Top 10 protein hits from the intact IgG obtained from blood plasma of donor 1.

Accession	-10lgP	Coverage (%)	#Peptides	#Unique	Avg. Mass	Description
P0DOX5|IGG1_HUMAN	38941	60	44	18	49329	Immunoglobulin gamma-1 heavy chain PE=1 SV=2
P01860|IGHG3_HUMAN	32064	59	29	7	41287	Immunoglobulin heavy constant gamma 3 GN=IGHG3 PE=1 SV=2
P01861|IGHG4_HUMAN	31454	66	25	2	35941	Immunoglobulin heavy constant gamma 4 GN=IGHG4 PE=1 SV=1
P01859|IGHG2_HUMAN	30885	74	28	7	35901	Immunoglobulin heavy constant gamma 2 GN=IGHG2 PE=1 SV=2
P02768|ALBU_HUMAN	28173	46	23	23	69367	Albumin GN=ALB PE=1 SV=2
P0DOY2|IGLC2_HUMAN	27965	86	13	3	11294	Immunoglobulin lambda constant 2 GN=IGLC2 PE=1 SV=1
P0DOY3|IGLC3_HUMAN	27965	86	13	3	11266	Immunoglobulin lambda constant 3 GN=IGLC3 PE=1 SV=1
P01024|CO3_HUMAN	26197	12	17	17	187147	Complement C3 GN=C3 PE=1 SV=2
P0C0L5|CO4B_HUMAN	24708	11	16	16	192750	Complement C4-B GN=C4B PE=1 SV=2
P01619|KV320_HUMAN	23273	63	9	3	12557	Immunoglobulin kappa variable 3-20 GN=IGKV3-20 PE=1 SV=2

Supplementary Table 3:Top 10 protein hits from the Fab fraction obtained from blood plasma of donor 1.

Accession	-10lgP	Coverage (%)	#Peptides	#Unique	Avg. Mass	Description
P0DOX5|IGG1_HUMAN	29319	51	27	14	49329	Immunoglobulin gamma-1 heavy chain PE=1 SV=2
P01024|CO3_HUMAN	28995	18	26	26	187147	Complement C3 GN=C3 PE=1 SV=2
P02768|ALBU_HUMAN	28991	53	29	29	69367	Albumin GN=ALB PE=1 SV=2
P01861|IGHG4_HUMAN	25723	55	16	3	35941	Immunoglobulin heavy constant gamma 4 GN=IGHG4 PE=1 SV=1
P01860|IGHG3_HUMAN	25213	39	15	3	41287	Immunoglobulin heavy constant gamma 3 GN=IGHG3 PE=1 SV=2
P0DOY2|IGLC2_HUMAN	24176	68	10	2	11294	Immunoglobulin lambda constant 2 GN=IGLC2 PE=1 SV=1
P0DOY3|IGLC3_HUMAN	24176	68	10	2	11266	Immunoglobulin lambda constant 3 GN=IGLC3 PE=1 SV=1
P01859|IGHG2_HUMAN	24072	46	15	3	35901	Immunoglobulin heavy constant gamma 2 GN=IGHG2 PE=1 SV=2
P01619|KV320_HUMAN	24030	55	8	4	12557	Immunoglobulin kappa variable 3-20 GN=IGKV3-20 PE=1 SV=2
P0DOX8|IGL1_HUMAN	23470	36	10	2	22830	Immunoglobulin lambda-1 light chain PE=1 SV=1

Supplementary Table 4:Top 10 protein hits from the Fc fraction obtained from blood plasma of donor 1.

Accession	-10lgP	Coverage (%)	#Peptides	#Unique	Avg. Mass	Description
P0DOX5|IGG1_HUMAN	37522	57	52	19	49329	Immunoglobulin gamma-1 heavy chain PE=1 SV=2
P01860|IGHG3_HUMAN	34225	49	32	8	41287	Immunoglobulin heavy constant gamma 3 GN=IGHG3 PE=1 SV=2
P01861|IGHG4_HUMAN	33300	58	40	7	35941	Immunoglobulin heavy constant gamma 4 GN=IGHG4 PE=1 SV=1
P01859|IGHG2_HUMAN	32568	56	38	10	35901	Immunoglobulin heavy constant gamma 2 GN=IGHG2 PE=1 SV=2
P0DOX7|IGK_HUMAN	27713	57	17	14	23379	Immunoglobulin kappa light chain PE=1 SV=1
P02768|ALBU_HUMAN	22621	26	15	15	69367	Albumin GN=ALB PE=1 SV=2
P0C0L5|CO4B_HUMAN	20435	8	10	10	192750	Complement C4-B GN=C4B PE=1 SV=2
P01024|CO3_HUMAN	18825	6	9	9	187147	Complement C3 GN=C3 PE=1 SV=2
P01871|IGHM_HUMAN	17384	16	6	6	49440	Immunoglobulin heavy constant mu GN=IGHM PE=1 SV=4
P0DOX2|IGA2_HUMAN	15981	13	7	4	48934	Immunoglobulin alpha-2 heavy chain PE=1 SV=2

4 Identification of proteins in bands from non-reducing SDS-PAGE

Intact IgG, Fab and Fc samples obtained from the blood plasma of donor 1 were analyzed by non-reducing SDS-PAGE as described before by Hennig et al. [4]. Tryptic peptides were produced from cut out protein bands as described by Kolodziej et al. [3] and analyzed similarly to peptides derived from samples in solution. After exclusion of ubiquitous and commonly identified skin-derived proteins like keratin, the up to top 10 hits are presented in the following tables. In all descriptions, the redundant entry " OS=Homo sapiens OX=9606" was removed for better readability.

Supplementary Table 5:Top 10 protein hits from band 4-1

Accession	-10lgP	Coverage (%)	#Peptides	#Unique	Avg. Mass	Description
P0DOX5|IGG1_HUMAN	35493	61	42	18	49329	Immunoglobulin gamma-1 heavy chain PE=1 SV=2
P01861|IGHG4_HUMAN	32748	68	34	8	35941	Immunoglobulin heavy constant gamma 4 GN=IGHG4 PE=1 SV=1
P0DOX7|IGK_HUMAN	32252	60	21	16	23379	Immunoglobulin kappa light chain PE=1 SV=1
P01859|IGHG2_HUMAN	30820	61	37	12	35901	Immunoglobulin heavy constant gamma 2 GN=IGHG2 PE=1 SV=2
P01860|IGHG3_HUMAN	30132	51	23	4	41287	Immunoglobulin heavy constant gamma 3 GN=IGHG3 PE=1 SV=2
P0DOY2|IGLC2_HUMAN	22646	81	9	2	11294	Immunoglobulin lambda constant 2 GN=IGLC2 PE=1 SV=1
P0DOY3|IGLC3_HUMAN	22646	81	9	2	11266	Immunoglobulin lambda constant 3 GN=IGLC3 PE=1 SV=1
B9A064|IGLL5_HUMAN	22255	41	9	3	23063	Immunoglobulin lambda-like polypeptide 5 GN=IGLL5 PE=2 SV=2
P0DOX8|IGL1_HUMAN	22255	40	9	3	22830	Immunoglobulin lambda-1 light chain PE=1 SV=1
P01619|KV320_HUMAN	19899	56	7	2	12557	Immunoglobulin kappa variable 3-20 GN=IGKV3-20 PE=1 SV=2

Supplementary Table 6:Top 10 protein hits from band 4-2

Accession	-10lgP	Coverage (%)	#Peptides	#Unique	Avg. Mass	Description
P0DOX5|IGG1_HUMAN	38119	63	48	17	49329	Immunoglobulin gamma-1 heavy chain PE=1 SV=2
P0DOX7|IGK_HUMAN	36409	54	19	4	23379	Immunoglobulin kappa light chain PE=1 SV=1
P01861|IGHG4_HUMAN	36124	71	37	6	35941	Immunoglobulin heavy constant gamma 4 GN=IGHG4 PE=1 SV=1
P01860|IGHG3_HUMAN	36096	62	35	8	41287	Immunoglobulin heavy constant gamma 3 GN=IGHG3 PE=1 SV=2
P01859|IGHG2_HUMAN	35120	69	36	7	35901	Immunoglobulin heavy constant gamma 2 GN=IGHG2 PE=1 SV=2
P01619|KV320_HUMAN	23381	49	9	5	12557	Immunoglobulin kappa variable 3-20 GN=IGKV3-20 PE=1 SV=2
P0DOX2|IGA2_HUMAN	20099	11	4	2	48934	Immunoglobulin alpha-2 heavy chain PE=1 SV=2
P0DOY2|IGLC2_HUMAN	20083	75	7	2	11294	Immunoglobulin lambda constant 2 GN=IGLC2 PE=1 SV=1
P0DOY3|IGLC3_HUMAN	20083	75	7	2	11266	Immunoglobulin lambda constant 3 GN=IGLC3 PE=1 SV=1
B9A064|IGLL5_HUMAN	19760	38	8	3	23063	Immunoglobulin lambda-like polypeptide 5 GN=IGLL5 PE=2 SV=2

Supplementary Table 7:Top 10 protein hits from band 4-3

Accession	-10lgP	Coverage (%)	#Peptides	#Unique	Avg. Mass	Description
P0DOX5|IGG1_HUMAN	38525	65	52	19	49329	Immunoglobulin gamma-1 heavy chain PE=1 SV=2
P01861|IGHG4_HUMAN	35227	64	33	3	35941	Immunoglobulin heavy constant gamma 4 GN=IGHG4 PE=1 SV=1
P01860|IGHG3_HUMAN	33794	53	31	3	41287	Immunoglobulin heavy constant gamma 3 GN=IGHG3 PE=1 SV=2
P01859|IGHG2_HUMAN	32729	61	32	7	35901	Immunoglobulin heavy constant gamma 2 GN=IGHG2 PE=1 SV=2
P0DOX7|IGK_HUMAN	30442	51	12	10	23379	Immunoglobulin kappa light chain PE=1 SV=1
P02768|ALBU_HUMAN	22048	20	12	12	69367	Albumin GN=ALB PE=1 SV=2
B9A064|IGLL5_HUMAN	17737	29	5	2	23063	Immunoglobulin lambda-like polypeptide 5 GN=IGLL5 PE=2 SV=2
P0DOX8|IGL1_HUMAN	17737	29	5	2	22830	Immunoglobulin lambda-1 light chain PE=1 SV=1
P01780|HV307_HUMAN	16689	39	6	2	12943	Immunoglobulin heavy variable 3-7 GN=IGHV3-7 PE=1 SV=2
P0DOX2|IGA2_HUMAN	16467	9	4	2	48934	Immunoglobulin alpha-2 heavy chain PE=1 SV=2

Supplementary Table 8:Top 10 protein hits from band 4-4

Accession	-10lgP	Coverage (%)	#Peptides	#Unique	Avg. Mass	Description
P0DOX5|IGG1_HUMAN	29410	47	26	10	49329	Immunoglobulin gamma-1 heavy chain PE=1 SV=2
P01861|IGHG4_HUMAN	27414	53	19	3	35941	Immunoglobulin heavy constant gamma 4 GN=IGHG4 PE=1 SV=1
P01859|IGHG2_HUMAN	25233	48	18	3	35901	Immunoglobulin heavy constant gamma 2 GN=IGHG2 PE=1 SV=2
P01860|IGHG3_HUMAN	23952	37	15	2	41287	Immunoglobulin heavy constant gamma 3 GN=IGHG3 PE=1 SV=2
P02768|ALBU_HUMAN	23090	21	10	10	69367	Albumin GN=ALB PE=1 SV=2
B9A064|IGLL5_HUMAN	17045	25	5	2	23063	Immunoglobulin lambda-like polypeptide 5 GN=IGLL5 PE=2 SV=2
P0DOX8|IGL1_HUMAN	17045	25	5	2	22830	Immunoglobulin lambda-1 light chain PE=1 SV=1
A0A0B4J1Y9|HV372_HUMAN	15989	36	4	2	13203	Immunoglobulin heavy variable 3-72 GN=IGHV3-72 PE=3 SV=1
P80748|LV321_HUMAN	14607	42	4	3	12446	Immunoglobulin lambda variable 3-21 GN=IGLV3-21 PE=1 SV=2
P01876|IGHA1_HUMAN	14071	14	4	3	37655	Immunoglobulin heavy constant alpha 1 GN=IGHA1 PE=1 SV=2

Supplementary Table 9:Top 10 protein hits from band 4-5

Accession	-10lgP	Coverage (%)	#Peptides	#Unique	Avg. Mass	Description
P0DOX5|IGG1_HUMAN	35336	49	36	12	49329	Immunoglobulin gamma-1 heavy chain PE=1 SV=2
P01861|IGHG4_HUMAN	34163	63	31	5	35941	Immunoglobulin heavy constant gamma 4 GN=IGHG4 PE=1 SV=1
P01859|IGHG2_HUMAN	30829	51	25	6	35901	Immunoglobulin heavy constant gamma 2 GN=IGHG2 PE=1 SV=2
P0DOX7|IGK_HUMAN	22234	41	7	6	23379	Immunoglobulin kappa light chain PE=1 SV=1
P0DOX8|IGL1_HUMAN	17793	30	6	2	22830	Immunoglobulin lambda-1 light chain PE=1 SV=1
P0DOX2|IGA2_HUMAN	16317	8	4	2	48934	Immunoglobulin alpha-2 heavy chain PE=1 SV=2
P01780|HV307_HUMAN	16034	39	7	2	12943	Immunoglobulin heavy variable 3-7 GN=IGHV3-7 PE=1 SV=2
P01825|HV459_HUMAN	16014	36	5	2	12936	Immunoglobulin heavy variable 4-59 GN=IGHV4-59 PE=1 SV=2
A0A0C4DH41|HV461_HUMAN	16014	36	5	2	13066	Immunoglobulin heavy variable 4-61 GN=IGHV4-61 PE=3 SV=1
A0A0B4J2H0|HV69D_HUMAN	14653	26	3	2	12660	Immunoglobulin heavy variable 1-69D GN=IGHV1-69D PE=1 SV=1

Supplementary Table 10: Top 10 protein hits from band 4-6

Accession	-10lgP	Coverage (%)	#Peptides	#Unique	Avg. Mass	Description
P01619|KV320_HUMAN	23987	56	8	3	12557	Immunoglobulin kappa variable 3-20 GN=IGKV3-20 PE=1 SV=2
P0DOX5|IGG1_HUMAN	19710	17	5	4	49329	Immunoglobulin gamma-1 heavy chain PE=1 SV=2
A0A075B6S9|KV137_HUMAN	19612	29	8	6	12688	Probable non-functional immunoglobulinn kappa variable 1-37 GN=IGKV1-37 PE=1 SV=7
P0DSN7|KVD37_HUMAN	19612	29	8	6	12688	Probable non-functional immunoglobulinn kappa variable 1D-37 GN=IGKV1D-37 PE=1 SV=1
P04430|KV116_HUMAN	19436	44	6	2	12618	Immunoglobulin kappa variable 1-16 GN=IGKV1-16 PE=1 SV=2
P06312|KV401_HUMAN	18212	37	7	7	13380	Immunoglobulin kappa variable 4-1 GN=IGKV4-1 PE=1 SV=1
P02647|APOA1_HUMAN	17550	24	6	6	30778	Apolipoprotein A-I GN=APOA1 PE=1 SV=1
P01714|LV319_HUMAN	17334	44	4	4	12042	Immunoglobulin lambda variable 3-19 GN=IGLV3-19 PE=1 SV=2
A0A0C4DH69|KV109_HUMAN	17257	42	5	2	12715	Immunoglobulin kappa variable 1-9 GN=IGKV1-9 PE=3 SV=1
P01615|KVD28_HUMAN	17137	51	5	2	12957	Immunoglobulin kappa variable 2D-28 GN=IGKV2D-28 PE=1 SV=2

Supplementary Table 11: Top 10 protein hits from band 5-1

Accession	-10lgP	Coverage (%)	#Peptides	#Unique	Avg. Mass	Description
P0DOX7|IGK_HUMAN	36926	65	26	2	23379	Immunoglobulin kappa light chain PE=1 SV=1
P0DOX5|IGG1_HUMAN	27432	28	19	10	49329	Immunoglobulin gamma-1 heavy chain PE=1 SV=2
P01859|IGHG2_HUMAN	25028	31	14	3	35901	Immunoglobulin heavy constant gamma 2 GN=IGHG2 PE=1 SV=2
P01860|IGHG3_HUMAN	24353	27	13	3	41287	Immunoglobulin heavy constant gamma 3 GN=IGHG3 PE=1 SV=2
P01861|IGHG4_HUMAN	24016	29	13	2	35941	Immunoglobulin heavy constant gamma 4 GN=IGHG4 PE=1 SV=1
A0A0C4DH73|KV112_HUMAN	19350	29	5	3	12645	Immunoglobulin kappa variable 1-12 GN=IGKV1-12 PE=3 SV=1
P01611|KVD12_HUMAN	19350	29	5	3	12620	Immunoglobulin kappa variable 1D-12 GN=IGKV1D-12 PE=1 SV=2
A0A0C4DH38|HV551_HUMAN	19077	57	6	2	12675	Immunoglobulin heavy variable 5-51 GN=IGHV5-51 PE=3 SV=1
P06312|KV401_HUMAN	18789	45	6	6	13380	Immunoglobulin kappa variable 4-1 GN=IGKV4-1 PE=1 SV=1
P01602|KV105_HUMAN	18258	38	5	2	12782	Immunoglobulin kappa variable 1-5 GN=IGKV1-5 PE=1 SV=2

Supplementary Table 12: Top 10 protein hits from band 5-2

Accession	-10lgP	Coverage (%)	#Peptides	#Unique	Avg. Mass	Description
P01860|IGHG3_HUMAN	20476	24	10	2	41287	Immunoglobulin heavy constant gamma 3 GN=IGHG3 PE=1 SV=2
P0DOX5|IGG1_HUMAN	20460	24	11	3	49329	Immunoglobulin gamma-1 heavy chain PE=1 SV=2
P01619|KV320_HUMAN	18874	46	5	2	12557	Immunoglobulin kappa variable 3-20 GN=IGKV3-20 PE=1 SV=2
P02768|ALBU_HUMAN	18612	15	9	9	69367	Albumin GN=ALB PE=1 SV=2
P0DOX2|IGA2_HUMAN	17695	10	6	2	48934	Immunoglobulin alpha-2 heavy chain PE=1 SV=2
P80748|LV321_HUMAN	16056	42	4	3	12446	Immunoglobulin lambda variable 3-21 GN=IGLV3-21 PE=1 SV=2
P06312|KV401_HUMAN	16012	30	4	4	13380	Immunoglobulin kappa variable 4-1 GN=IGKV4-1 PE=1 SV=1
P04430|KV116_HUMAN	14992	41	4	2	12618	Immunoglobulin kappa variable 1-16 GN=IGKV1-16 PE=1 SV=2
A0A0C4DH42|HV366_HUMAN	14533	32	5	2	12698	Immunoglobulin heavy variable 3-66 GN=IGHV3-66 PE=3 SV=1
P01594|KV133_HUMAN	14493	29	4	2	12848	Immunoglobulin kappa variable 1-33 GN=IGKV1-33 PE=1 SV=2

Supplementary Table 13: Top 10 protein hits from band 5-3

Accession	-10lgP	Coverage (%)	#Peptides	#Unique	Avg. Mass	Description
P0DOX7|IGK_HUMAN	33406	53	16	14	23379	Immunoglobulin kappa light chain PE=1 SV=1
P02768|ALBU_HUMAN	27199	34	20	20	69367	Albumin GN=ALB PE=1 SV=2
P0DOX5|IGG1_HUMAN	27140	42	19	9	49329	Immunoglobulin gamma-1 heavy chain PE=1 SV=2
P01861|IGHG4_HUMAN	23196	35	11	2	35941	Immunoglobulin heavy constant gamma 4 GN=IGHG4 PE=1 SV=1
P01860|IGHG3_HUMAN	23169	23	11	2	41287	Immunoglobulin heavy constant gamma 3 GN=IGHG3 PE=1 SV=2
P01619|KV320_HUMAN	19255	37	4	3	12557	Immunoglobulin kappa variable 3-20 GN=IGKV3-20 PE=1 SV=2
P06312|KV401_HUMAN	18857	35	5	5	13380	Immunoglobulin kappa variable 4-1 GN=IGKV4-1 PE=1 SV=1
A0A075B6S5|KV127_HUMAN	18670	38	6	3	12712	Immunoglobulin kappa variable 1-27 GN=IGKV1-27 PE=3 SV=1
P01825|HV459_HUMAN	18456	34	5	2	12936	Immunoglobulin heavy variable 4-59 GN=IGHV4-59 PE=1 SV=2
A0A0C4DH41|HV461_HUMAN	18456	33	5	2	13066	Immunoglobulin heavy variable 4-61 GN=IGHV4-61 PE=3 SV=1

Supplementary Table 14: Top 10 protein hits from band 5-4

Accession	-10lgP	Coverage (%)	#Peptides	#Unique	Avg. Mass	Description
P02768|ALBU_HUMAN	31635	55	39	39	69367	Albumin GN=ALB PE=1 SV=2
P0DOX7|IGK_HUMAN	21415	41	7	6	23379	Immunoglobulin kappa light chain PE=1 SV=1
P0DOX5|IGG1_HUMAN	18972	30	12	3	49329	Immunoglobulin gamma-1 heavy chain PE=1 SV=2
P01009|A1AT_HUMAN	17327	26	10	10	46737	Alpha-1-antitrypsin GN=SERPINA1 PE=1 SV=3
P0DOY2|IGLC2_HUMAN	14926	46	3	3	11294	Immunoglobulin lambda constant 2 GN=IGLC2 PE=1 SV=1
P0CF74|IGLC6_HUMAN	14926	46	3	3	11277	Immunoglobulin lambda constant 6 GN=IGLC6 PE=1 SV=1
P0DOY3|IGLC3_HUMAN	14926	46	3	3	11266	Immunoglobulin lambda constant 3 GN=IGLC3 PE=1 SV=1
P01876|IGHA1_HUMAN	13106	11	4	2	37655	Immunoglobulin heavy constant alpha 1 GN=IGHA1 PE=1 SV=2
P01594|KV133_HUMAN	10630	29	2	2	12848	Immunoglobulin kappa variable 1-33 GN=IGKV1-33 PE=1 SV=2
P01593|KVD33_HUMAN	10630	29	2	2	12848	Immunoglobulin kappa variable 1D-33 GN=IGKV1D-33 PE=1 SV=2

Supplementary Table 15: Top 10 protein hits from band 5-5

Accession	-10lgP	Coverage (%)	#Peptides	#Unique	Avg. Mass	Description
P01834|IGKC_HUMAN	32276	80	18	2	11765	Immunoglobulin kappa constant GN=IGKC PE=1 SV=2
P0DOY3|IGLC3_HUMAN	25735	81	8	2	11266	Immunoglobulin lambda constant 3 GN=IGLC3 PE=1 SV=1
P0DOX5|IGG1_HUMAN	25529	38	18	8	49329	Immunoglobulin gamma-1 heavy chain PE=1 SV=2
P01764|HV323_HUMAN	20313	61	11	2	12582	Immunoglobulin heavy variable 3-23 GN=IGHV3-23 PE=1 SV=2
P04433|KV311_HUMAN	19582	53	6	2	12575	Immunoglobulin kappa variable 3-11 GN=IGKV3-11 PE=1 SV=1
P01861|IGHG4_HUMAN	19330	34	12	2	35941	Immunoglobulin heavy constant gamma 4 GN=IGHG4 PE=1 SV=1
P01859|IGHG2_HUMAN	19118	35	13	4	35901	Immunoglobulin heavy constant gamma 2 GN=IGHG2 PE=1 SV=2
A0A0C4DH42|HV366_HUMAN	18965	52	10	2	12698	Immunoglobulin heavy variable 3-66 GN=IGHV3-66 PE=3 SV=1
A0A0C4DH33|HV124_HUMAN	18815	54	4	3	12824	Immunoglobulin heavy variable 1-24 GN=IGHV1-24 PE=3 SV=1
P01624|KV315_HUMAN	18608	42	7	4	12496	Immunoglobulin kappa variable 3-15 GN=IGKV3-15 PE=1 SV=2

Supplementary Table 16: Top 10 protein hits from band 5-6

Accession	-10lgP	Coverage (%)	#Peptides	#Unique	Avg. Mass	Description
P0DOX7|IGK_HUMAN	40412	54	33	2	23379	Immunoglobulin kappa light chain PE=1 SV=1
P01834|IGKC_HUMAN	39022	91	30	2	11765	Immunoglobulin kappa constant GN=IGKC PE=1 SV=2
P0DOX8|IGL1_HUMAN	27542	45	17	3	22830	Immunoglobulin lambda-1 light chain PE=1 SV=1
P0DOX5|IGG1_HUMAN	25256	35	16	8	49329	Immunoglobulin gamma-1 heavy chain PE=1 SV=2
A0A075B6K5|LV39_HUMAN	20514	49	6	3	12332	Immunoglobulin lambda variable 3-9 GN=IGLV3-9 PE=3 SV=1
P01718|LV327_HUMAN	19628	42	7	3	12165	Immunoglobulin lambda variable 3-27 GN=IGLV3-27 PE=1 SV=2
P01717|LV325_HUMAN	19594	52	9	5	12011	Immunoglobulin lambda variable 3-25 GN=IGLV3-25 PE=1 SV=2
P06312|KV401_HUMAN	19121	56	7	6	13380	Immunoglobulin kappa variable 4-1 GN=IGKV4-1 PE=1 SV=1
P01594|KV133_HUMAN	18690	39	5	2	12848	Immunoglobulin kappa variable 1-33 GN=IGKV1-33 PE=1 SV=2
P01593|KVD33_HUMAN	18690	39	5	2	12848	Immunoglobulin kappa variable 1D-33 GN=IGKV1D-33 PE=1 SV=2

Supplementary Table 17: Top 10 protein hits from band 6-1

Accession	-10lgP	Coverage (%)	#Peptides	#Unique	Avg. Mass	Description
P0DOX5|IGG1_HUMAN	29608	40	19	4	49329	Immunoglobulin gamma-1 heavy chain PE=1 SV=2
P01861|IGHG4_HUMAN	29352	52	18	2	35941	Immunoglobulin heavy constant gamma 4 GN=IGHG4 PE=1 SV=1
P0DOX7|IGK_HUMAN	28077	49	15	13	23379	Immunoglobulin kappa light chain PE=1 SV=1
P01859|IGHG2_HUMAN	26738	56	20	4	35901	Immunoglobulin heavy constant gamma 2 GN=IGHG2 PE=1 SV=2
P01619|KV320_HUMAN	18708	52	5	2	12557	Immunoglobulin kappa variable 3-20 GN=IGKV3-20 PE=1 SV=2
P02768|ALBU_HUMAN	16656	11	7	7	69367	Albumin GN=ALB PE=1 SV=2
P80748|LV321_HUMAN	14610	32	3	2	12446	Immunoglobulin lambda variable 3-21 GN=IGLV3-21 PE=1 SV=2
P01717|LV325_HUMAN	13416	27	3	2	12011	Immunoglobulin lambda variable 3-25 GN=IGLV3-25 PE=1 SV=2
P01700|LV147_HUMAN	12305	25	4	4	12284	Immunoglobulin lambda variable 1-47 GN=IGLV1-47 PE=1 SV=2
A0A0B4J1V0|HV315_HUMAN	12046	20	2	2	12926	Immunoglobulin heavy variable 3-15 GN=IGHV3-15 PE=3 SV=1

Supplementary Table 18: Seven protein hits from band 6-2

Accession	-10lgP	Coverage (%)	#Peptides	#Unique	Avg. Mass	Description
P01857|IGHG1_HUMAN	41093	64	72	17	36106	Immunoglobulin heavy constant gamma 1 GN=IGHG1 PE=1 SV=1
P0DOX5|IGG1_HUMAN	41093	47	72	17	49329	Immunoglobulin gamma-1 heavy chain PE=1 SV=2
P01859|IGHG2_HUMAN	37092	53	57	16	35901	Immunoglobulin heavy constant gamma 2 GN=IGHG2 PE=1 SV=2
P01861|IGHG4_HUMAN	36548	52	51	8	35941	Immunoglobulin heavy constant gamma 4 GN=IGHG4 PE=1 SV=1
P01860|IGHG3_HUMAN	33359	46	43	4	41287	Immunoglobulin heavy constant gamma 3 GN=IGHG3 PE=1 SV=2
P01834|IGKC_HUMAN	19269	44	3	3	11765	Immunoglobulin kappa constant GN=IGKC PE=1 SV=2
P0DOX7|IGK_HUMAN	19269	22	3	3	23379	Immunoglobulin kappa light chain PE=1 SV=1

5 Exoglycosidase digests and EDGE-profiling of blood plasma, intact IgG, Fc and IgA

Blood plasma, intact IgG, IgG-Fab and IgG-Fc of donor 1, as well as IgA (Athens Research & Technology Georgia, USA) were digested with α2-3,6,8,9 neuraminidase A (SiaA) and β(1-3,4)-galactosidase (GALase) according to the manufacturer's instructions (New England Biolabs Inc., Ipswich, USA). Subsequently, the samples were subjected to epitope-directed glycan enrichment (EDGE-)profiling, using the previously described sulfatase F1-ORF13 [5,6]. This enzyme binds to 6-*O*-sulfated *N*-acetylglucosamine (GlcNAc) when in its apo-sulfatase state, lacking Ca^{2+} ions as a cofactor, without cleaving off the sulfate group off. Using this property in a filter-assisted EDGE-profiling workflow, the non-sulfated *N*-glycans are depleted and found in the flow-through, while the sulfated *N*-glycans are highly enriched in the elution fraction.

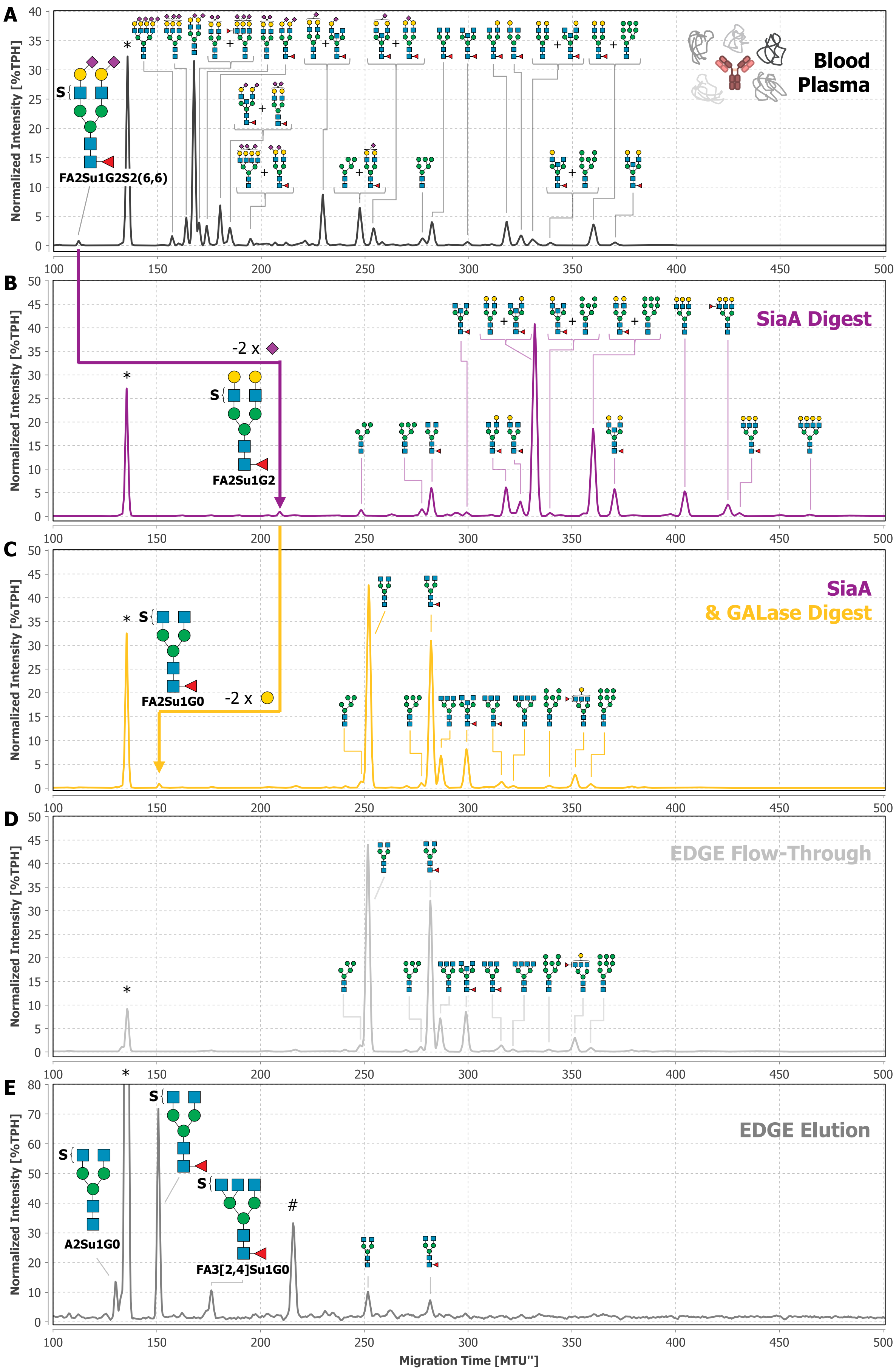

A
B
C
D
E
Normalized Intensity [%TPH]
Migration Time [MTU'']
FA2Su1G2S2(6,6)
Blood Plasma
SiaA Digest
-2 x
FA2Su1G2
SiaA & GALase Digest
FA2Su1G0
EDGE Flow-Through
EDGE Elution
A2Su1G0
FA3[2,4]Su1G0

Supplementary Figure 8: Exoglycosidase digests and EDGE-profiling of blood plasma-derived *N*-glycans. Blood plasma-derived *N*-glycans from donor 1 (**A**) were digested with SiaA (**B**). Upon digestion with GALase (**C**), 6-*O*-sulfated and non-sulfated GlcNAcs are exposed as the terminal sugar moieties. Epitope-directed glycan enrichment (EDGE-)profiling was used to separate non-sulfated *N*-glycans (**D**) from sulfated *N*-glycans (**E**). This workflow also identified and proved the presence of FA2Su1G2S2(6,6) (peak at ~112 MTU") in blood plasma. Due to the enrichment of sulfated *N*-glycans in the EDGE-elution, A2Su1G0 (peak at ~131 MTU") and FA3[2,4]Su1G0 (peak at ~176 MTU") were detected, too. * Migration time alignment standard. # unknown impurity introduced by enzyme solution (see explanation in **Suppl. Fig 12**). Blue squares: GlcNAc. Red triangles: fucose. Green circles: mannose. Yellow circles: galactose. Purple diamonds: Neu5Ac (α2,6-linked when tilted to the right, α2,3-linked when tilted to the left). S indicates a sulfate group. *N*-glycans that comigrate in a single peak are indicated by brackets and "+".

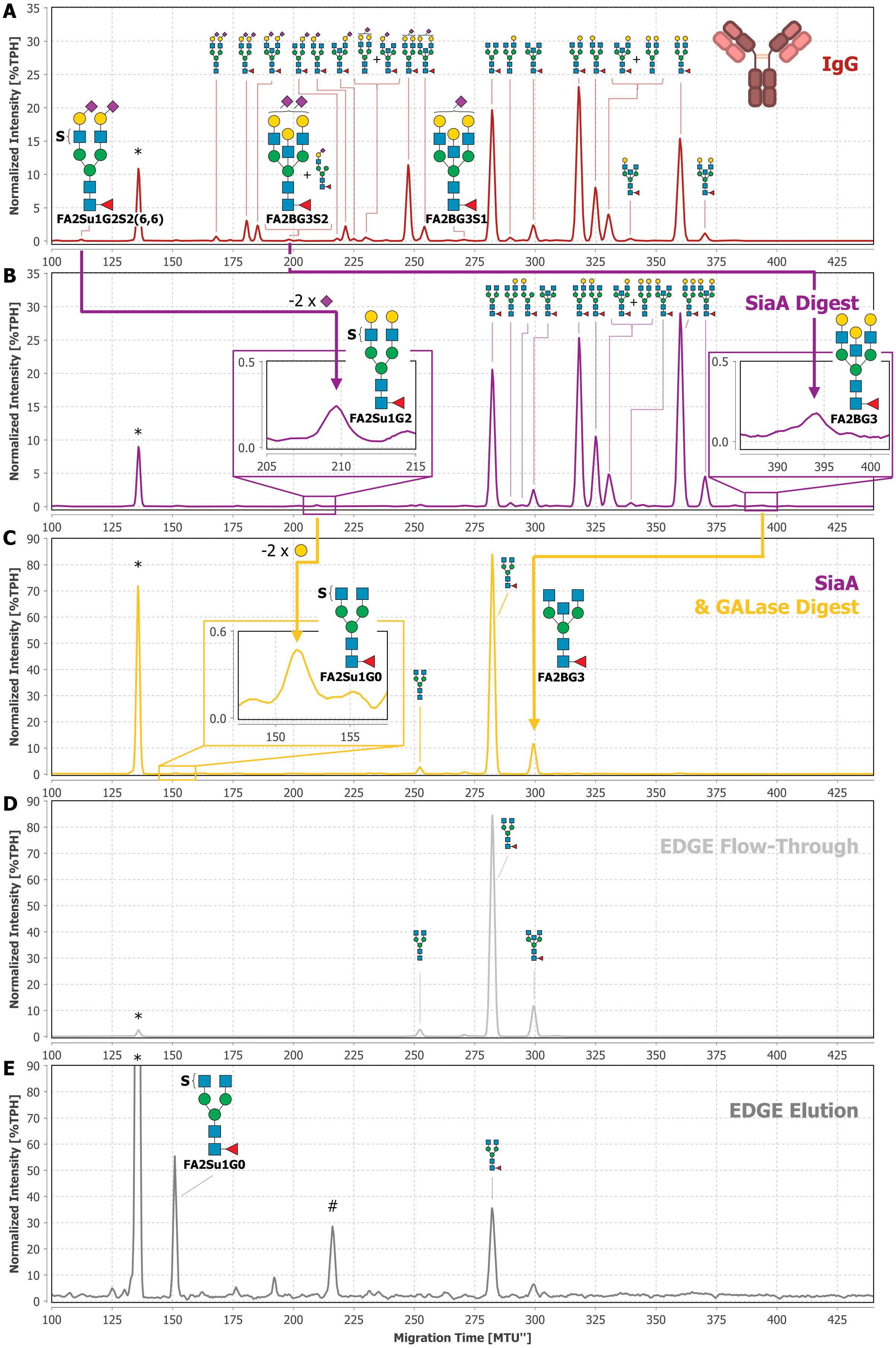
A
B
C
D
E
IgG
SiaA Digest
SiaA
& GALase Digest
EDGE Flow-Through
EDGE Elution
FA2Su1G2S2(6,6)
FA2BG3S2
FA2BG3S1
FA2Su1G2
FA2BG3
FA2Su1G0
-2 x
Normalized Intensity [%TPH]
Migration Time [MTU'']

Supplementary Figure 9: Exoglycosidase digests and EDGE-profiling of intact IgG-derived *N*-glycans. *N*-glycans derived from intact IgG of donor 1 (**A**) were digested with SiaA (**B**). Upon digestion with GALase (**C**), 6-*O*-sulfated and non-sulfated GlcNAcs are exposed as the terminal sugar moieties. Epitope-directed glycan enrichment (EDGE-)profiling was used to separate non-sulfated *N*-glycans (**D**) from sulfated *N*-glycans (**E**). FA2Su1G2S2(6,6) (peak at ~112 MTU") could also be identified and proven on IgG by this workflow. Also, FA2BG3S2 (peak at ~197 MTU"), FA2BG3S1 (peak at ~271 MTU") and FA2BG3 (peak at ~395 MTU") could be identified among the intact IgG-derived *N*-glycans before and after SiaA digest, respectively. * Migration time alignment standard. # unknown impurity introduced by enzyme solution (see explanation in **Suppl. Fig 12**). Blue squares: GlcNAc. Red triangles: fucose. Green circles: mannose. Yellow circles: galactose. Purple diamonds: Neu5Ac (α2,6-linked when tilted to the right). S indicates a sulfate group. *N*-glycans that comigrate in a single peak are indicated by brackets and “+”.

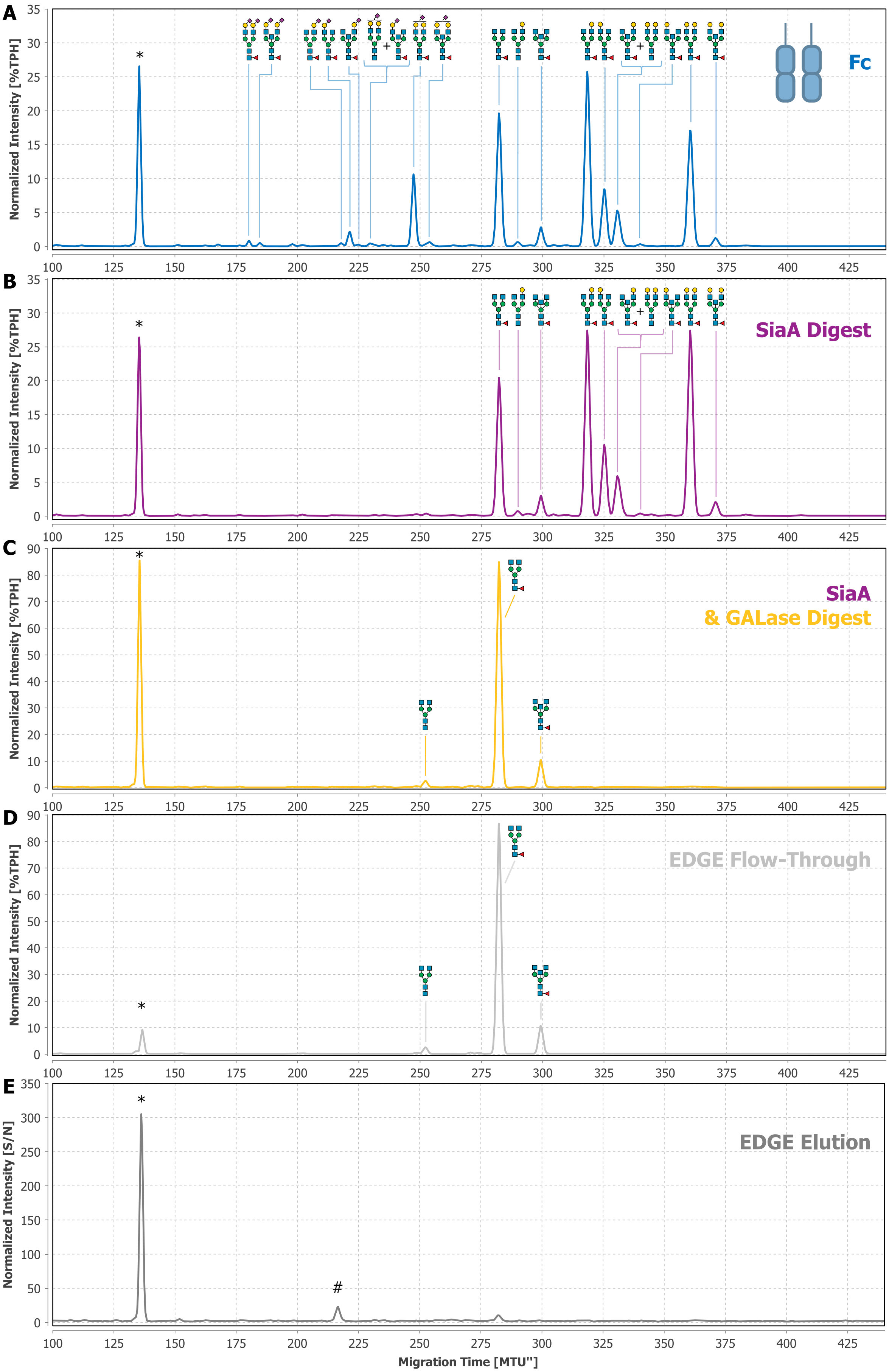
A
B
C
D
E
Fc
SiaA Digest
SiaA
& GALase Digest
EDGE Flow-Through
EDGE Elution
Normalized Intensity [%TPH]
Normalized Intensity [S/N]
Migration Time [MTU'']

Supplementary Figure 10: Exoglycosidase digests and EDGE-profiling of Fc-derived *N*-glycans. *N*-glycans derived from Fc of donor 1 (**A**) were digested with SiaA (**B**) and GALase (**C**), No peaks corresponding to *N*-glycans with sulfation or bisecting LacNAc can be observed. Hence, only *N*-glycans with non-sulfated GlcNAcs as the terminal sugar moieties are produced. These are readily found in the flowthrough fraction of the epitope-directed glycan enrichment (EDGE-)profiling (**D**). Accordingly, also no sulfated *N*-glycans are enriched in the elution fraction of EDGE-profiling (**E**). * Migration time alignment standard. # unknown impurity introduced by enzyme solution (see explanation in **Suppl. Fig 12**). Blue squares: GlcNAc. Red triangles: fucose. Green circles: mannose. Yellow circles: galactose. Purple diamonds: Neu5Ac (α2,6-linked when tilted to the right). S indicates a sulfate group. *N*-glycans that comigrate in a single peak are indicated by brackets and "+".

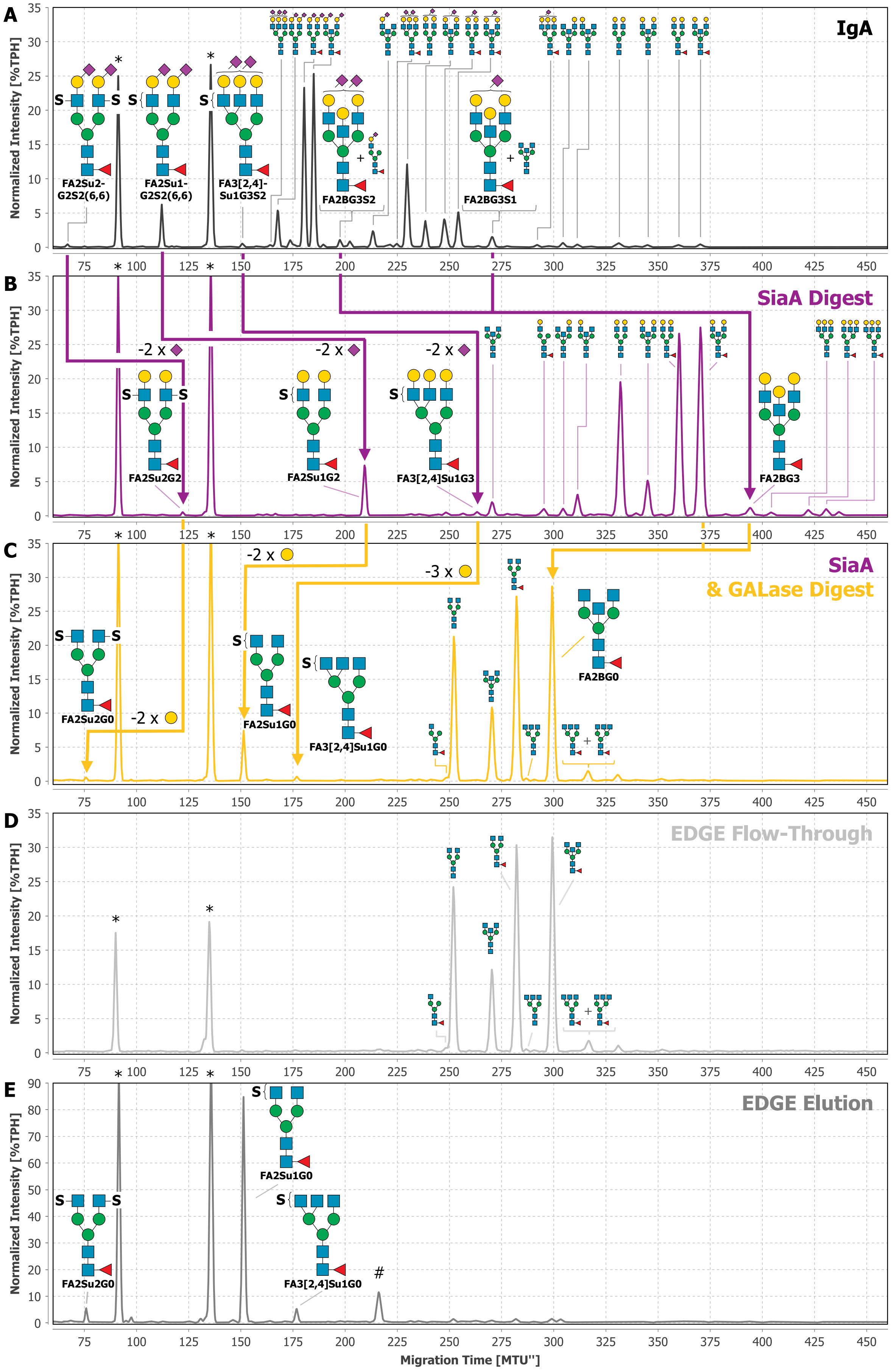
A
B
C
D
E
IgA
SiaA Digest
SiaA
& GALase Digest
EDGE Flow-Through
EDGE Elution
Normalized Intensity [%TPH]
Migration Time [MTU'']
FA2Su2-G2S2(6,6)
FA2Su1-G2S2(6,6)
FA3[2,4]-Su1G3S2
FA2BG3S2
FA2BG3S1
FA2Su2G2
FA2Su1G2
FA3[2,4]Su1G3
FA2BG3
-2 x
-3 x
FA2Su2G0
FA2Su1G0
FA3[2,4]Su1G0
FA2BG0
#

Supplementary Figure 11: Exoglycosidase digests and EDGE-profiling of IgA-derived *N*-glycans. *N*-glycans derived from IgA (Athens Research & Technology, Georgia, USA, **A**) were digested with SiaA (**B**). Thus, the three sulfated *N*-glycans FA2Su2G2S2(6,6) (peak at ~67 MTU"), FA2Su1G2S2(6,6) (peak at ~112 MTU") and FA3[2,4]Su1G3S2 (peak at ~151 MTU") each lose two sialic acids (purple arrows). In the SiaA digest, also the *N*-glycans FA2BG3S2 (peak at ~197 MTU") and FA2BG3S1 (peak at ~271 MTU") with a bisecting LacNAc lose one or two sialic acids, respectively, so that a FA2BG3 peak appears at ~395 MTU". Upon digestion with GALase (**C**), terminal galactoses are cleaved off (yellow arrows), and 6-*O*-sulfated and non-sulfated GlcNAcs are exposed as the terminal sugar moieties. Epitope-directed glycan enrichment (EDGE-)profiling was used to separate non-sulfated *N*-glycans in the flow-through (**D**) from sulfated *N*-glycans in the elution (**E**). * Migration time alignment standard. # Unknown impurity introduced by enzyme solution (see explanation in **Suppl. Figure 12**). Blue squares: GlcNAc. Red triangles: fucose. Green circles: mannose. Yellow circles: galactose. Purple diamonds: Neu5Ac (α2,6-linked when tilted to the right, α2,3-linked when tilted to the left). S indicates a sulfate group. *N*-glycans that comigrate in a single peak are indicated by brackets and "+".

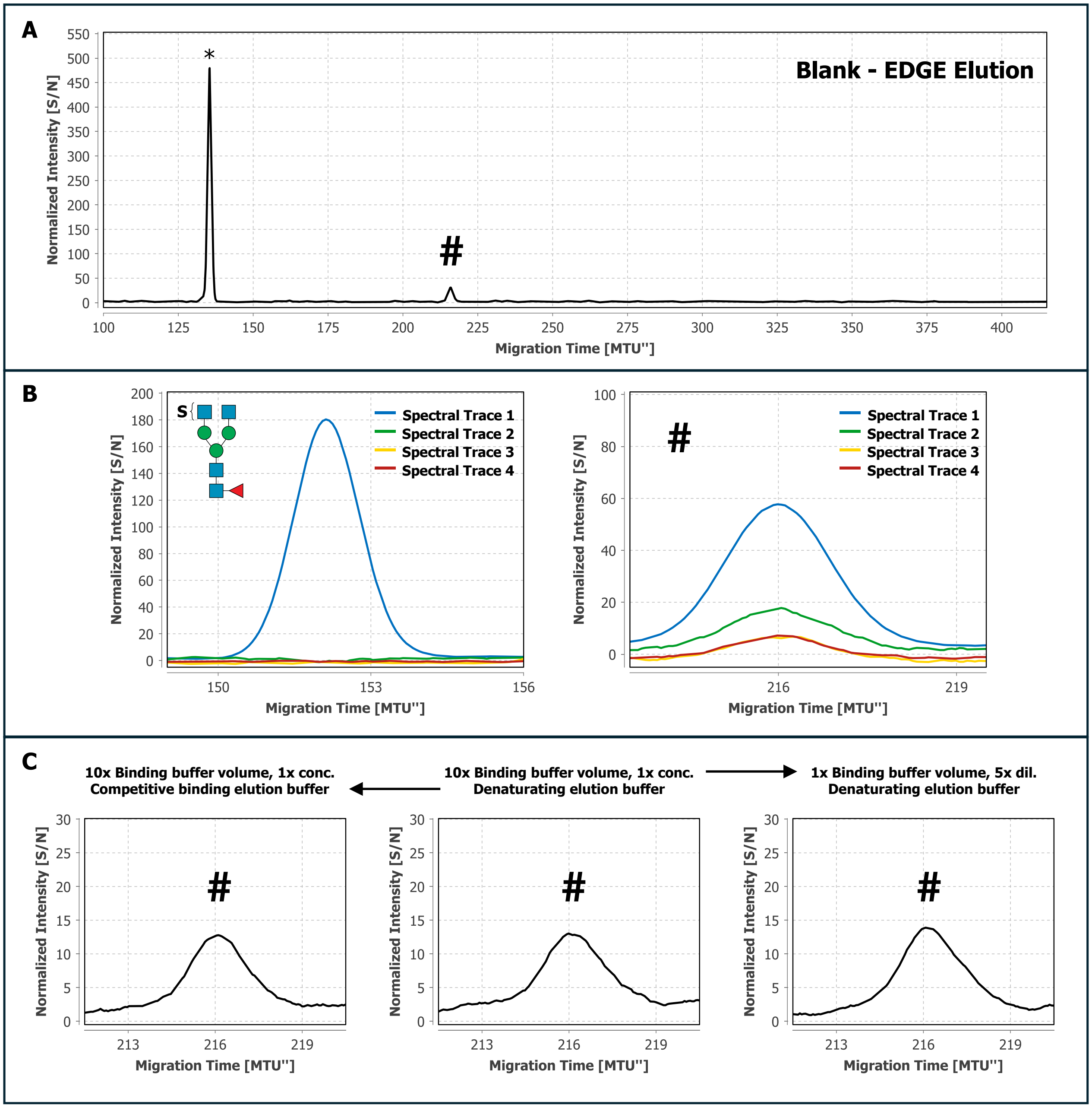

Supplementary Figure 12: Identification of an unknown peak appearing in EDGE-profiling as a non-glycan impurity. During EDGE-profiling of all samples, an additional peak appeared at ~216 MTU'' and was marked with an #. This peak also appeared in the EDGE-elution of a blank sample (**A**), in which ultra-pure water was used instead of a sample containing APTS-labeled *N*-glycans. During xCGE-LIF analysis, fluorescent signals are recorded in different spectral traces. Exemplarily, an overlay of spectral traces (**B**) of the EDGE-Elution of donor 1's Fab (**Figure 6** in the main manuscript) is shown. On a glyXboxCE™ system (glyXera GmbH, Magdeburg, Germany), APTS-labeled *N*-glycans are detected in spectral trace 1 (blue graphs). At a signal-to-noise normalized intensity of ~180 S/N, the FA2Su1G0 peak produces no crosstalk into the other spectral traces. In the same measurement, the unknown impurity peak causes signals in the other spectral traces, although its intensity of ~60 S/N is only 1/3 of the FA2Su1G0 peak. Comparison of different binding buffer amounts and elution conditions (**C**) showed no differences in the intensity of the impurity peak. In the left graph, the denaturing elution buffer (based on DTT and SDS)

was substituted with an elution buffer containing 6-*O*-sulfated GlcNAc that works based on competitive binding. The right graph shows an approach in which the amount of binding buffer components was reduced by using a 10-fold lower volume of 5-fold diluted binding buffer. Thus, the components of the binding buffer and elution buffer can be excluded as the origin of the impurity peak. All the aforementioned data indicates that the peak does not arise from an APTS-labeled *N*-glycan but originates from the enzyme solution as the only constant in the performed experiments. * Migration time alignment standard. Blue squares: GlcNAc. Red triangles: fucose. Green circles: mannose. S indicates a sulfate group.

6 Exoglycosidase digests to verify the identification of FA2BG3

Throughout our analyses, we observed a peak at approximately ~395 MTU" after desialylation of the intact IgG-, Fab- and IgA-derived *N*-glycans (**Suppl. Figure 9**, **Figure 5** and **Suppl. Figure 11**, respectively). This peak can also be seen in the F(ab')$_2$ sample, that was already considerably desialylated on delivery (Athens Research & Technology Georgia, USA, **Suppl. Figure 7B**). The migration time of this peak matched no *N*-glycans typically found in humans or that would fit *N*-glycans identified before SiaA and after GALase digests. Therefore, this peak was thoroughly investigated using additional exoglycosidases, namely β-*N*-acetylglucosaminidase S (GlcNAcase), α1-2,4,6 fucosidase O (FucO), and α1-3,4 fucosidase (all New England Biolabs Inc., Ipswich, USA). Also, we excluded the possibility that the peak is constituted by a hybrid type glycan by GlcNAcase or α1-2,3,6 mannosidase (MANase) digests (New England Biolabs Inc., Ipswich, USA) of *N*-glycans after SiaA and GALase digests.

After evaluation of all these exoglycosidase digests, we identified the peak as a core-fucosylated, fully galactosylated, diantennary *N*-glycan, also containing a galactosylated bisecting GlcNAc, i.e., a bisecting LacNAc. As this structure has not yet been described in the nomenclature system used here, this *N*-glycan was named FA2BG3.

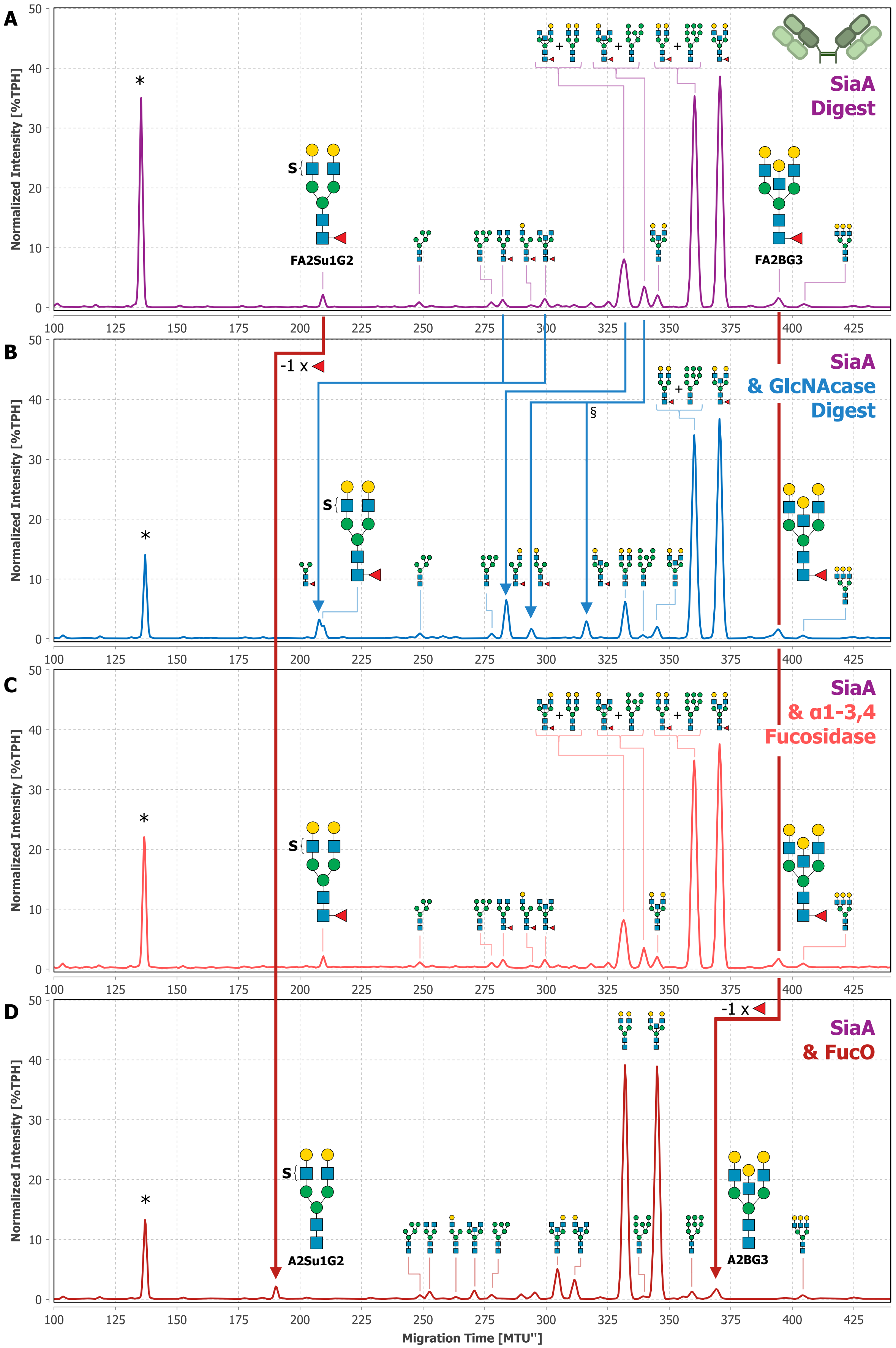
A
B
C
D
Normalized Intensity [%TPH]
Migration Time [MTU'']
SiaA
Digest
SiaA
& GlcNAcase
Digest
SiaA
& α1-3,4
Fucosidase
SiaA
& FucO
FA2Su1G2
FA2BG3
A2Su1G2
A2BG3
-1 x
§
*

Supplementary Figure 13: Additional exoglycosidase digests of desialylated Fab-derived *N*-glycans. Fab-derived *N*-glycans of donor 1 after SiaA digest (**A**) were additionally digested with GlcNAcase (**B**), α1-3,4 fucosidase (**C**), or FucO (**D**). The intriguing peak at ~395MTU" does not shift in the GlcNAcase or α1-3,4 fucosidase digest, but shifts to ~369 MTU" upon FucO digest, indicating the loss of a core fucose resulting in the structure A2BG3. * Migration time alignment standard. § Partial GlcNAcase digest: The bisecting GlcNAc is more difficult to cleave off if the 3'-arm is galactosylated. Blue squares: GlcNAc. Red triangles: fucose. Green circles: mannose. Yellow circles: galactose. S indicates a sulfate group. *N*-glycans that comigrate in a single peak are indicated by brackets and "+".

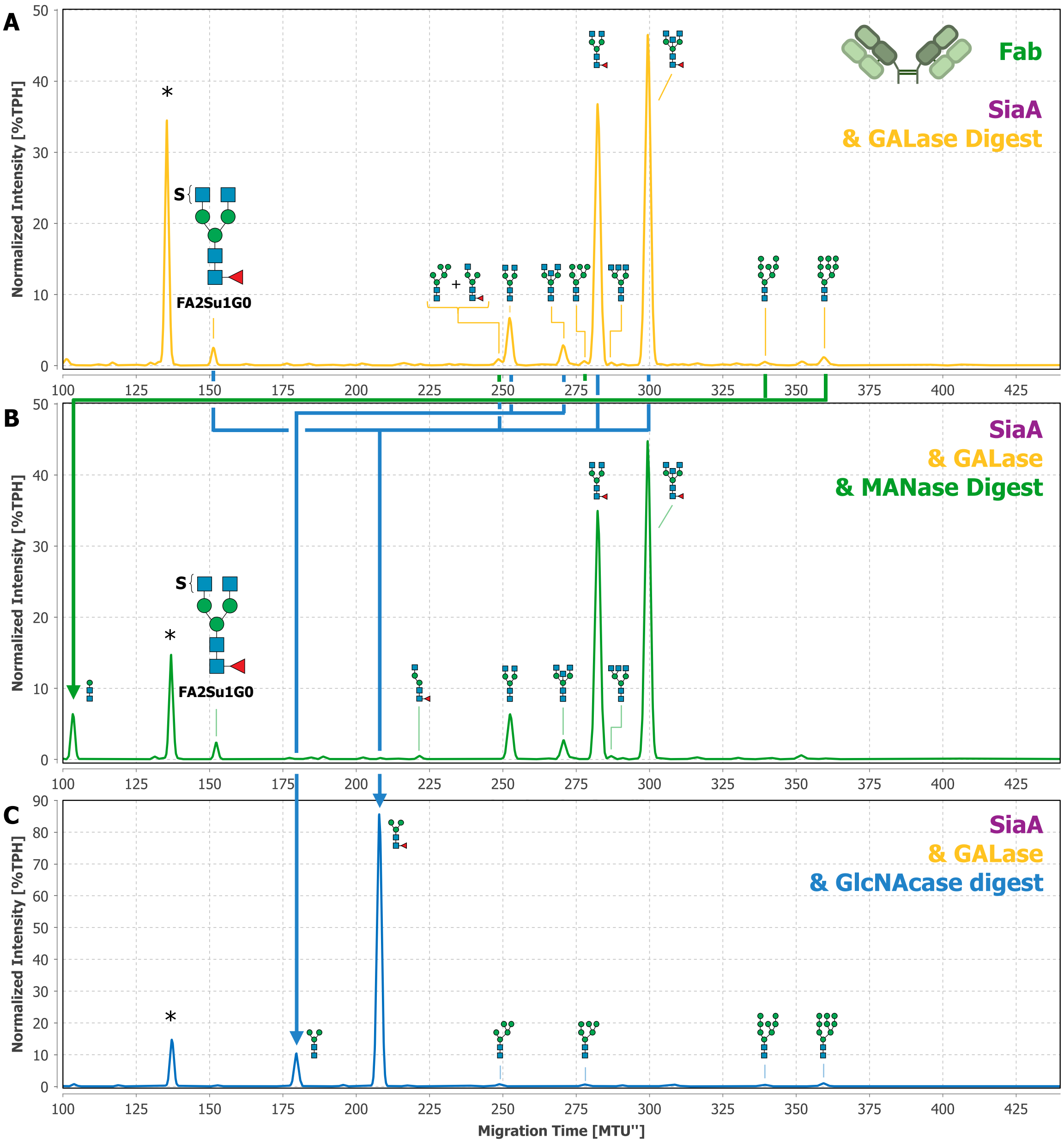

Supplementary Figure 14: Additional MANase and GlcNAcase digests of Fab-derived *N*-glycans after SiaA and GALase digests. Fab-derived *N*-glycans of donor 1 after SiaA and GALase digests (**A**) were additionally digested with MANase (**B**) or GlcNAcase (**C**). In the MANase digest, only minor amounts of Man2-FA1[3]G0 (peak at ~221 MTU'') as a final digestion product of core fucosylated hybrid-type *N*-glycans are found. These can be explained by FA1[3]G1S1(6) that was identified in the untreated Fab-derived *N*-glycans, inconveniently comigrating in a peak with FA2BG3S2 at ~197 MTU'' (**Figure 5**). After GlcNAcase digest, only Man3, FMan3, and oligomannose-type *N*-glycans are detected. Therefore, a large, core fucosylated hybrid-type *N*-glycan can be ruled out as a possible constituent of the peak at ~395 MTU''. * Migration time alignment standard. Blue squares: GlcNAc. Red triangles: fucose. Green circles: mannose. S indicates a sulfate group. *N*-glycans that comigrate in a single peak are indicated by brackets and "+".

7 Relative quantification of Fab-derived *N*-glycans

As a comprehensive overview the relative quantification data of *N*-glycans derived from the Fab fragments are provided below in **Suppl. Table 19**. Fab fragments were obtained by affinity chromatography-based capturing of human IgG from blood plasma or intact IgG samples and subsequent proteolytic fragmentation with IdeZ.

Supplementary Table 19: Relative quantification data of Fab-derived *N*-glycans. All peaks that are picked using the glycoanalysis software glyXtoolCE™ (glyXera GmbH, Magdeburg, Germany) in at least one of the produced Fab samples are listed consecutively in order of their migration times. *N*-glycan data is provided for Fab samples derived from Donor 1 (ID: ND105; LOT: 673760816), Donor 2 (ID: ND96; LOT: 667210816), Donor 3 (ID: ND116; LOT: 667600816), from the two Frozen Normal Control Plasma samples (VisuCon™-F, Batches 0009-52FCP and 0012-52FCP), and from the two commercial intact IgG samples (Athens Research & Technology, Batches IG1802-R22 and IG2017-01). Peak annotations are based on initial migration time matching and subsequent exoglycosidase digests (SiaA, GALase, MANase, GlcNAcase, and FucO). Peak heights were normalized to the total peak height of all picked peaks, resulting in % of the total peak height (%TPH). n/a indicates that a peak was not present in the respective sample or was not picked (e.g., because it was <LOQ). Blue squares: GlcNAc. Red triangles: fucose. Green circles: mannose. Yellow circles: galactose. Purple diamonds: Neu5Ac (α2,6-linked when tilted upwards, α2,3-linked when tilted downwards). S indicates a sulfate group. *N*-glycans that comigrate in a single peak are listed in the same row with their respective glycan structures, names and GlyTouCan IDs.

Glycan Structure	Glycan Name	GlyTouCan ID	Normalized Signal Intensity [%TPH] on Fabs of						
			Donor 1	Donor 2	Donor 3	Control Plasma 0009-52FCP	Control Plasma 0012-52FCP	Intact IgG IG1802-R22	Intact IgG IG2017-01
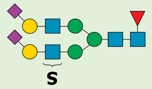	**FA2Su1G2S2(6,6)**	**G17413FR**	**1.82**	**2.51**	**1.93**	**1.06**	**1.24**	**0.39**	**0.50**
	**?**		**0.35**	**0.17**	**0.21**	**0.35**	**0.79**	**n/a**	**n/a**
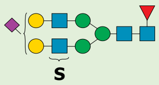	**FA2Su1G2S1(6)**	**G79162YU**	**0.42**	**0.34**	**0.25**	**n/a**	**n/a**	**n/a**	**n/a**
	**?**								

Glycan Structure	Glycan Name	GlyTouCan ID	Normalized Signal Intensity [%TPH] on Fabs of Donor 1	Donor 2	Donor 3	Control Plasma 0009-52FCP	Control Plasma 0012-52FCP	Intact IgG IG1802-R22	Intact IgG IG2017-01
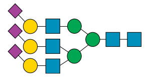	A3[2,4]G3S3(6,6,6)	G19444JC	n/a	0.16	0.24	0.29	n/a	n/a	n/a
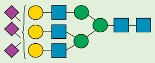	A3[2,4]G3S3(3,6,6)	G47023TK	0.48	0.48	0.43	0.55	n/a	n/a	n/a
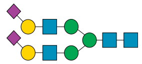	A2G2S2(6,6)	G84467IZ	4.56	3.68	5.41	5.39	3.99	1.96	2.25
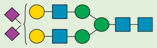<br>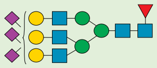	A2G2S1(6)S1(3)<br>FA3[2,4]G3S3(3,6,6)	G27668QQ<br>G83275PK	0.39	n/a	0.50	0.49	n/a	n/a	n/a
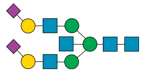	A2BG2S2(6,6)	G50779LX	1.29	1.06	1.17	1.60	1.78	1.61	1.72
	?		n/a	0.15	n/a	n/a	n/a	n/a	n/a
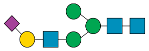	A1[3]G1S1(6)	G06209KS	n/a	n/a	0.19	n/a	0.72	0.20	0.29
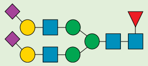	FA2G2S2(6,6)	G56749GV	23.27	25.65	21.59	19.27	16.11	20.53	21.83
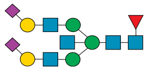	FA2BG2S2(6,6)	G14127XU	20.20	20.23	18.68	24.75	24.80	24.79	24.68

Glycan Structure	Glycan Name	GlyTouCan ID	Normalized Signal Intensity [%TPH] on Fabs of						
			Donor 1	Donor 2	Donor 3	Control Plasma 0009-52FCP	Control Plasma 0012-52FCP	Intact IgG IG1802-R22	Intact IgG IG2017-01
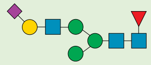	FA1[6]G1S1(3)	G30109UP	0.31	0.43	0.38	0.33	n/a	0.46	0.44
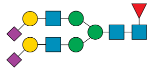	FA2G2S2(3,3)	G17689DH	n/a	0.24	0.30	n/a	n/a	n/a	n/a
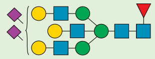	FA2BG3S2	G40475CA	1.02	0.91	0.99	1.11	1.10	1.22	1.27
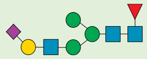	FA1[3]G1S1(6)	G84852GW							
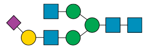	A2G1[3]S1(6)	G04791QM	n/a	n/a	0.15	n/a	n/a	n/a	n/a
	?		n/a	n/a	n/a	n/a	2.03	n/a	n/a
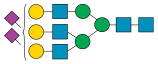	A3[2,4]G3S2(6,6)	G30879ZB	n/a	n/a	n/a	n/a	n/a	0.13	n/a
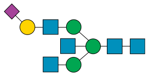	A2BG1[6]S1(6)	G78255XL							
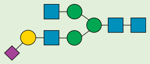	A2G1[3]S1(3)	G16759KR	n/a	0.13	0.15	0.31	0.71	0.26	0.28
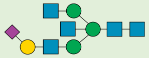	A2BG1[3]S1(6)	G22795QB							

Glycan Structure	Glycan Name	GlyTouCan ID	Normalized Signal Intensity [%TPH] on Fabs of						
			Donor 1	Donor 2	Donor 3	Control Plasma 0009-52FCP	Control Plasma 0012-52FCP	Intact IgG IG1802-R22	Intact IgG IG2017-01
	?		n/a	n/a	0.14	n/a	n/a	n/a	n/a
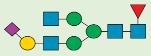	FA2G1[3]S1(6)	G43694RQ	0.37	0.54	1.04	0.44	n/a	0.46	0.53
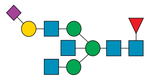	FA2BG1[6]S1(6)	G17848HM	1.37	1.01	0.90	1.78	1.83	1.33	1.19
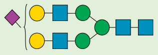	A2G2S1(6)	G87433AX	1.73	1.59	2.14	2.20	2.00	0.90	1.17
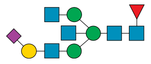	FA2BG1[3]S1(6)	G67942CH	2.05	1.45	1.61	2.38	2.59	2.20	1.98
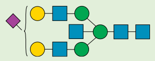	A2BG2S1(6)	G71380NP	0.89	0.87	0.77	1.37	3.90	1.57	1.60
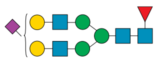<br>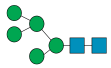	FA2G2S1(6)<br>Man5	G81413UE<br>G55220VL	10.80	11.96	10.33	7.55	6.65	8.94	9.12
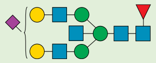	FA2BG2S1(6)	G86632ZU	16.75	15.32	12.94	17.11	18.41	19.91	18.50
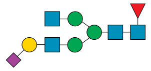	FA2G1[3]S1(3)	G68937TM	n/a	n/a	0.18	n/a	n/a	n/a	n/a

Glycan Structure	Glycan Name	GlyTouCan ID	Normalized Signal Intensity [%TPH] on Fabs of						
			Donor 1	Donor 2	Donor 3	Control Plasma 0009-52FCP	Control Plasma 0012-52FCP	Intact IgG IG1802-R22	Intact IgG IG2017-01
	?		n/a	0.19	n/a	n/a	n/a	n/a	n/a
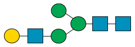	A1[3]G1	G69411IG	0.35	0.42	0.22	0.22	n/a	0.31	0.30
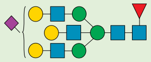	FA2BG3S1	G17927PV	0.96	0.93	0.81	1.06	2.47	1.28	1.21
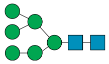	Man6	G80966KZ	0.55	0.29	0.37	0.43	n/a	0.28	n/a
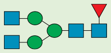	FA2G0	G80858MF	0.87	1.29	3.88	1.00	0.74	1.19	1.54
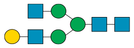	A2G1[3]	G66937TJ	n/a	n/a	0.03	n/a	n/a	n/a	n/a
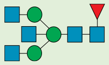	FA2BG0	G30159WR	0.97	0.52	1.24	1.17	1.53	0.88	0.86
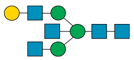	A2BG1[6]	G11535IB	n/a	n/a	n/a	0.23	n/a	0.21	n/a
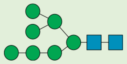	Man7[D1]	G68668TB	n/a	n/a	0.15	n/a	n/a	0.19	n/a

Glycan Structure	Glycan Name	GlyTouCan ID	Normalized Signal Intensity [%TPH] on Fabs of						
			Donor 1	Donor 2	Donor 3	Control Plasma 0009-52FCP	Control Plasma 0012-52FCP	Intact IgG IG1802-R22	Intact IgG IG2017-01
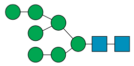	**Man7[D3]**	**G83161QT**	**n/a**	**n/a**	**0.14**	**0.22**	**n/a**	**0.20**	**n/a**
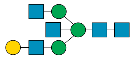	**A2BG1[3]**	**G72718TT**							
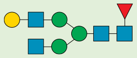	**FA2G1[6]**	**G27919IH**	**0.52**	**0.72**	**2.26**	**0.45**	**n/a**	**0.71**	**1.05**
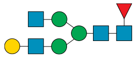	**FA2G1[3]**	**G58667NI**	**0.43**	**0.83**	**1.99**	**0.47**	**n/a**	**0.72**	**0.97**
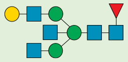	**FA2BG1[6]**	**G85767HW**	**2.19**	**1.40**	**1.62**	**2.05**	**2.26**	**1.86**	**1.73**
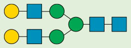	**A2G2**	**G36191CD**							
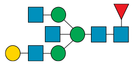	**FA2BG1[3]**	**G71013KY**	**0.98**	**0.75**	**0.75**	**1.00**	**1.10**	**1.03**	**0.90**
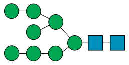	**Man8[D1D3]**	**G40702WU**							
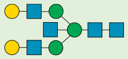	**A2BG2**	**G52934AK**	**n/a**	**0.14**	**n/a**	**0.22**	**n/a**	**0.25**	**0.25**

<table>
<tr><th rowspan="2">Glycan Structure</th><th rowspan="2">Glycan Name</th><th rowspan="2">GlyTouCan ID</th><th colspan="7">Normalized Signal Intensity [%TPH] on Fabs of</th></tr>
<tr><th>Donor 1</th><th>Donor 2</th><th>Donor 3</th><th>Control Plasma 0009-52FCP</th><th>Control Plasma 0012-52FCP</th><th>Intact IgG IG1802-R22</th><th>Intact IgG IG2017-01</th></tr>
<tr><td>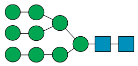</td><td>Man9</td><td>G60230HH</td><td rowspan="2">1.57</td><td rowspan="2">1.31</td><td rowspan="2">2.35</td><td rowspan="2">1.07</td><td rowspan="2">0.93</td><td rowspan="2">1.34</td><td rowspan="2">1.52</td></tr>
<tr><td>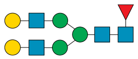</td><td>FA2G2</td><td>G78059CC</td></tr>
<tr><td>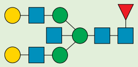</td><td>FA2BG2</td><td>G25520XG</td><td>2.55</td><td>2.18</td><td>1.58</td><td>2.07</td><td>2.33</td><td>2.55</td><td>2.32</td></tr>
<tr><td>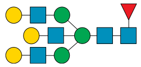</td><td>FA2BG3</td><td>G39408YK</td><td>n/a</td><td>0.13</td><td>n/a</td><td>n/a</td><td>n/a</td><td>0.15</td><td>n/a</td></tr>
<tr><td>Sum</td><td></td><td></td><td>100.00</td><td>100.00</td><td>100.00</td><td>100.00</td><td>100.00</td><td>100.00</td><td>100.00</td></tr>
</table>

8 HILIC-UPLC-FLD analysis of human IgG-derived *N*-glycans

Human IgG-derived *N*-glycans were analyzed by hydrophilic interaction liquid chromatography (HILIC) ultra-high performance liquid chromatography (UPLC) with fluorescence detection (FLD) (HILIC-UPLC-FLD) similar to a protocol described previously [7]. Briefly, *N*-glycans were labeled for analysis with 2-aminobenzamide (2-AB) as published by Ruhaak and coworkers [8]. Excess 2-AB label, reducing agent, salts and impurities were removed from the samples using cellulose HILIC-SPE [9]. 2-AB-labeled *N*-glycans were analyzed by HILIC-UPLC-FLD similar to a Waters application note [10]. Briefly, fluorescently labeled *N*-glycans were separated by HILIC-UPLC on a Shimadzu Nexera X2 system (Shimadzu Deutschland GmbH, Duisburg, Germany), equipped with a Waters Acquity BEH Glycan column (particle size: 1.7 µm; column size: 150 mm length × 2.1 mm inner diameter). The effluent was monitored by fluorescence detection at an excitation wavelength of 330 nm and an emission wavelength of 420 nm. The column was held initially at 30 % mobile phase A (50 mM ammonium formate, pH 4.4) and 70 % mobile phase B (100 % ACN). Then, the previously described gradient [10] was applied: 0 min 70 % solvent B at 0.561 mL/min; 1.47 min 70 % solvent B; 24.81 min 53 % solvent B; 25.5 min 30 % solvent B at 0.4 mL/min; 29.5 min 30 % solvent B; 30.0 min 70 % solvent B at 0.561 mL/min; 40.0 min 70 % solvent B. For analysis, 1 µL of aqueous sample was injected. The retention times of eluting glycan peaks are given in minutes.

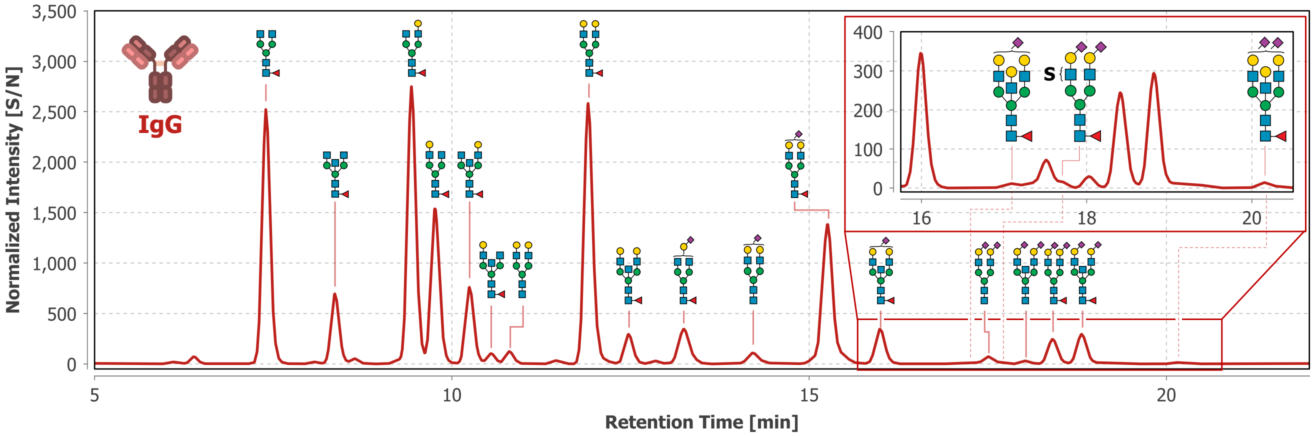

Supplementary Figure 15: HILIC-UPLC-FLD chromatogram of IgG-derived *N*-glycans Human IgG-derived *N*-glycans were also labeled with 2-AB analyzed by HILIC-UPLC-FLD. Also with this method, we found three low abundant peaks to which we could assign FA2BG3S1, FA2Su1G2S2(6,6) and FA2BG3S2. Blue squares: GlcNAc. Red triangles: fucose. Green circles: mannose. Yellow circles: galactose. Purple diamonds: Neu5Ac (α2,6-linked when tilted to the right). S indicates a sulfate group.

9 Remarks on the applicability of EDGE-profiling to unreleased *N*-glycans

In addition to the EDGE-profiling of released and APTS-labeled *N*-glycans, we have tested a vast array of pre-treatments using the (apo-)sulfatase before the release of *N*-glycans. These tests included mostly IgA as a sample from which sulfated glycopeptides with conserved glycosylation sites could be obtained, but also IgG.

The used sulfatase recognizes only terminal 6-*O*-sulfated GlcNAc, thus desialylation and degalactosylation of the unreleased *N*-glycans are prerequisite for all tested approaches. For the desialylation, the enzymatic approach using sialidase was compared to mild acid hydrolysis.

Furthermore, the EDGE-profiling was tested on tryptic glycopeptides, as well as on smaller glycopeptides produced by trypsin in combination with proteinase K. Glycopeptides were purified and/or enriched before the pretreatment with the sulfatase by Cotton-Tip HILIC, reversed phase chromatography with a C18 resin, or lectin-based enrichment of glycopeptides (using the *N*-glycopeptide binding protein, known as Fbs1-GYR lectin, New England Biolabs Inc., Ipswich).

None of these efforts resulted in the detection of sulfated glycopeptides in glycoproteomic analyses. In contrast, after subsequent release of *N*-glycans from glycopeptides, APTS-labeling and xCGE-LIF-based *N*-glycan analysis, we were able to detect sulfated *N*-glycans (data not shown). However, the enrichment effect was not very big, indicating considerably unspecific binding on the glycopeptide (or -protein) level, and the generated data was not of a quality that is suitable for publication.

10 Supplementary references

[1] Stavenhagen, K., Plomp, R., Wuhrer, M. *Anal Chem* 2015, *87*, 11691–11699.

[2] Chandler, K. B., Mehta, N., Leon, D. R., Suscovich, T. J., Alter, G., Costello, C. E. *Molecular and Cellular Proteomics* 2019, *18*, 686–703.

[3] Kolodziej, A., Smalla, K.-H., Richter, S., Engler, A., Pielot, R., Dieterich, D. C., Tischmeyer, W., Naumann, M., Kähne, T. *Journal of Visualized Experiments* 2016.

[4] Hennig, R., Rapp, E., Kottler, R., Cajic, S., Borowiak, M., Reichl, U. 2015, pp. 123–143.

[5] Chuzel, L. Application of functional metagenomics to the field of glycobiology. [PhD thesis]., Otto-von-Guericke-Universität Magdeburg, Magdeburg, 2021, Available online at: https://d-nb.info/1249017475/04

[6] Chuzel, L., Fossa, S. L., Boisvert, M. L., Cajic, S., Hennig, R., Ganatra, M. B., Reichl, U., Rapp, E., Taron, C. H. *Microb Cell Fact* 2021, *20*.

[7] Burock, R., Cajic, S., Hennig, R., Buettner, F. F. R., Reichl, U., Rapp, E. *Molecules* 2023, *28*, 1843.

[8] Ruhaak, L. R., Steenvoorden, E., Koeleman, C. A. M., Deelder, A. M., Wuhrer, M. *Proteomics* 2010, *10*, 2330–2336.

[9] Ruhaak, L. R., Huhn, C., Waterreus, W.-J., de Boer, A. R., Neusüss, C., Hokke, C. H., Deelder, A. M., Wuhrer, M. *Anal Chem* 2008, *80*, 6119–6126.

[10] Hilliard, M., Struwe, W., Adamczyk, B., Saldova, R., Yu, Y. Q., O'rourke, J., Carta, G., Rudd, P. *Development of a Glycan Database for Waters ACQUITY UPLC Systems*, 2012.
